# Genotype imputation and variability in polygenic risk score estimation

**DOI:** 10.1186/s13073-020-00801-x

**Published:** 2020-11-23

**Authors:** Shang-Fu Chen, Raquel Dias, Doug Evans, Elias L. Salfati, Shuchen Liu, Nathan E. Wineinger, Ali Torkamani

**Affiliations:** 1 Scripps Research Translational Institute, Scripps Research, La Jolla, CA 92037 USA; 2 Department of Integrative Structural and Computational Biology, Scripps Research, La Jolla, CA 92037 USA

**Keywords:** Genotype phasing, Genotype imputation, Polygenic risk score, PRS, Coronary artery disease, Polygenic score, Genetic risk score, Genome-wide score

## Abstract

**Background:**

Polygenic risk scores (PRSs) are a summarization of an individual’s genetic risk for a disease or trait. These scores are being generated in research and commercial settings to study how they may be used to guide healthcare decisions. PRSs should be updated as genetic knowledgebases improve; however, no guidelines exist for their generation or updating.

**Methods:**

Here, we characterize the variability introduced in PRS calculation by a common computational process used in their generation—genotype imputation. We evaluated PRS variability when performing genotype imputation using 3 different pre-phasing tools (Beagle, Eagle, SHAPEIT) and 2 different imputation tools (Beagle, Minimac4), relative to a WGS-based gold standard. Fourteen different PRSs spanning different disease architectures and PRS generation approaches were evaluated.

**Results:**

We find that genotype imputation can introduce variability in calculated PRSs at the individual level without any change to the underlying genetic model. The degree of variability introduced by genotype imputation differs across algorithms, where pre-phasing algorithms with stochastic elements introduce the greatest degree of score variability. In most cases, PRS variability due to imputation is minor (< 5 percentile rank change) and does not influence the interpretation of the score. PRS percentile fluctuations are also reduced in the more informative tails of the PRS distribution. However, in rare instances, PRS instability at the individual level can result in singular PRS calculations that differ substantially from a whole genome sequence-based gold standard score.

**Conclusions:**

Our study highlights some challenges in applying population genetics tools to individual-level genetic analysis including return of results. Rare individual-level variability events are masked by a high degree of overall score reproducibility at the population level. In order to avoid PRS result fluctuations during updates, we suggest that deterministic imputation processes or the average of multiple iterations of stochastic imputation processes be used to generate and deliver PRS results.

**Supplementary information:**

The online version contains supplementary material available at 10.1186/s13073-020-00801-x.

## Background

Polygenic risk scores (PRSs) are of increasing utility in early risk detection, risk stratification, therapy prioritization, and life-planning [1–3]. In practice, individualized PRS reports should be updated over time as the underlying genetic studies and resultant knowledgebases expand [4, 5]. This need to refresh PRSs is analogous to the need to update monogenic testing results as underlying knowledgebases of pathogenic variants evolve [6–8]. However, PRSs are continuous scores that typically use the entirety of an underlying knowledgebase for score calculation, whereas monogenic variant analysis and reporting involve the binary (or categorical) re-classification of a constrained set of rare variants present in any individual’s genome. As a result, the PRS update process may introduce fluctuations in any and possibly all individual-level scores, whereas updates to monogenic testing results typically involve the rarer re-clarification of ambiguous results (the re-classification of a variant of unknown significance). This major difference leads to special considerations for updating of PRSs.

Besides updates to genetic knowledgebases, the most commonly used basic computational approaches for PRS derivation can introduce variability in the resultant score. Currently, sparse genotyping approaches, like array-based SNP genotyping or low-pass whole genome sequencing (WGS), are the dominant assays used to obtain genetic data for PRS calculation. For example, tens of millions of individuals have pre-existing SNP array data acquired from direct-to-consumer genetic testing companies that can and is used to calculate PRSs. These technologies are inexpensive, a feature necessary for population-wide screening where PRSs would be most useful, suggesting sparse genotyping approaches will be the preferred mode of PRS deployment even as WGS costs decline. Sparse genotyping approaches require a genotype imputation step to infer the full set of genotypes included in PRS calculations. This imputation process has been demonstrated to produce scores that are highly correlated overall with PRSs derived from gold standard WGS data [9–12]. However, genotype imputation algorithms can include stochastic elements, which introduce variability in the inferred genotype dosages at the individual level. Here, we investigate the influence of this variability on the stability of individual PRS estimates for multiple genetic diseases with similar results. Results for coronary artery disease (CAD) are presented in the main text as our motivating example because of its emerging utility [13–15]. Results for multiple PRS derivation methods applied to other conditions of interest including atrial fibrillation, breast cancer, type 2 diabetes, glaucoma, and Alzheimer’s disease are provided in the *Supplementary Files*.

Overall, we find that genotype imputation introduces minor fluctuations in PRSs. While small fluctuations in a PRS may not materially influence risk estimates and resultant clinical/personal conclusions, these fluctuations may influence the perceived stability and/or confidence in these estimates. Moreover, the range of these fluctuations varies depending upon the imputation algorithms used, and in rare instances, these fluctuations can result in a score change exceeding 20 percentile points despite no change in the underlying genetic data. These rare large-scale fluctuations are not obvious when PRS reproducibility is evaluated at the population level but can lead to significant differences in the perception and interpretation of PRSs at the individual level. In other rare instances, score fluctuations result in the re-classification of individuals across risk tiers as determined by relative population rank thresholds. These rare score fluctuations would occur in population screening settings where thousands, if not millions, of individuals would receive PRS results and longitudinal updates. Here, we characterize PRS variability at the individual level and suggest the use of imputation algorithms with more deterministic behavior given that overall imputation accuracy is comparable across all algorithms.

## Methods

**Fig. 1:**
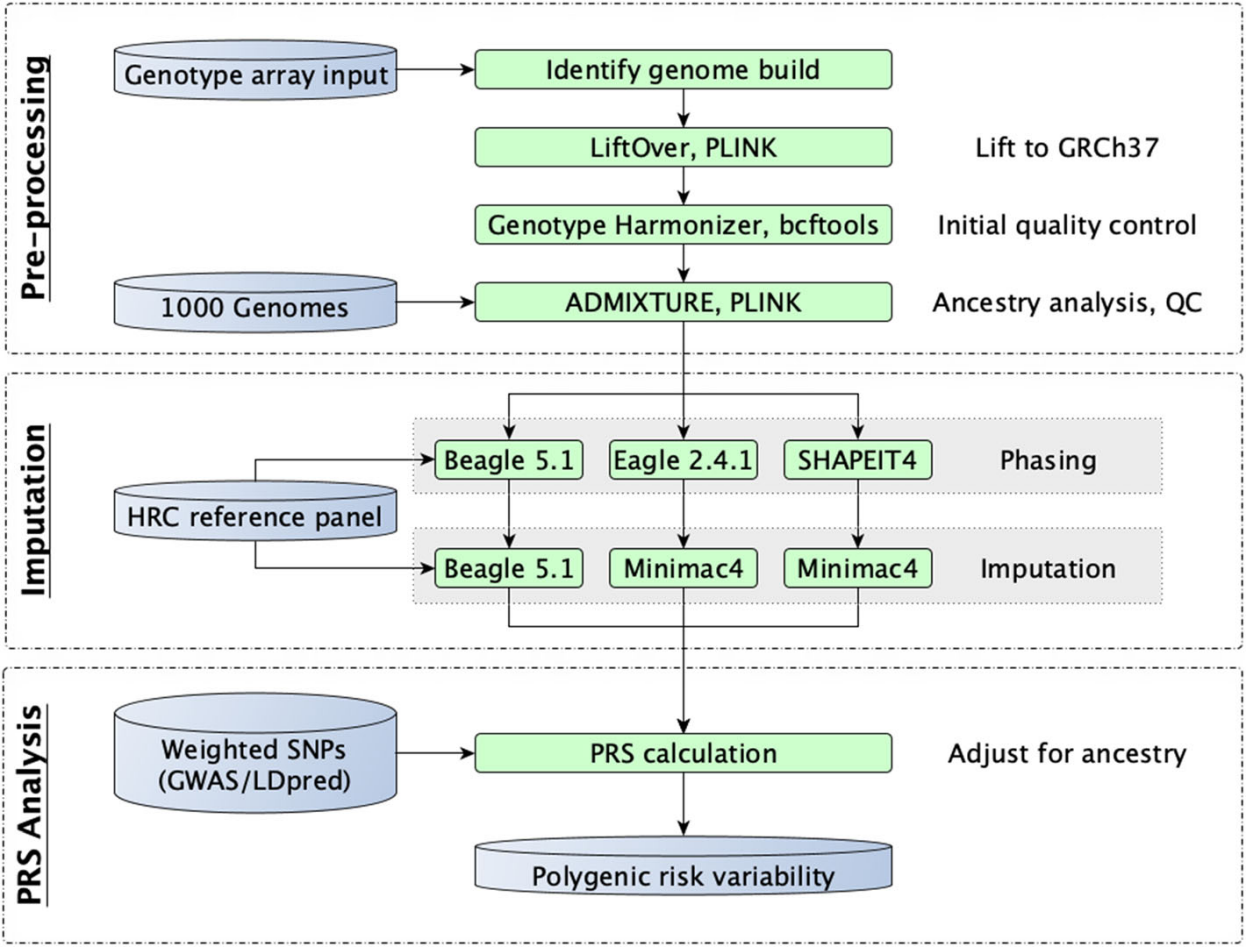
Study overview. A schematic overview of our polygenic risk computational process. Genetic data was standardized (pre-processing), underwent imputation using three different common genotype imputation processes (imputation), and PRS analysis (PRS analysis)

An overview of the computational process we executed is presented in Fig. 1.

### Genetic dataset and pre-processing

A total of 3574 individuals from the Atherosclerosis Risk in Communities (ARIC) [16] study with both array-based SNP genotype data and WGS data available were initially retrieved from National Center for Biotechnology Information dbGAP server (phs000280.v6.p1). Samples were genotyped on Genome-Wide Human SNP Array 6.0 chip with genotypes called using Birdseed calling algorithm [17] with samples and SNPs filtered using standard call rate, sex mismatch, and Hardy-Weinberg equilibrium thresholds as described previously [18]. To isolate algorithmic issues from of data format issues, we standardized all genotype data to forward strand GRCh37 orientation as is generated by variant calling from WGS data. Strand orientation was standardized using Strand Home (https://www.well.ox.ac.uk/~wrayner/strand/) strand and build files. Multi-allelic and duplicate variants were excluded using bcftools v1.9 with view (flags: -M 2 -m 2) and norm (flags: -d both) functions [19]. A total of 39,087 variants were excluded in this step. Any strand flips or unresolvable ambiguous variants were detected and corrected or removed using Genotype Harmonizer v1.4.20 with 1000 Genomes Phase3 v5 (1000G) providing the expected linkage disequilibrium (LD) relationships (flags: --callRateFilter 0.90 –keep). One hundred ninety-seven variants were corrected, and 53,109 variants were removed in this step. The remaining 70,715 strand ambiguous variants were eliminated with bcftools with +fixref plugin (flags: -m flip -d). A final round of filtering was applied to remaining indels, variants with missing alternative allele information, and multi-allelic variants using bcftools. Sixty variants were removed in this final step. A total of 678,849 variants of 841,820 original variants were retained for imputation.

For gold standard WGS-based variant calls, freeze 3 WGS variant calls were acquired from the Trans-Omics for Precision Medicine program. Variant calls were generated using the GATK best-practices v3.2-2 pipeline from TruSeq PCR-free libraries generated and sequenced at a number of different sequencing sites. For more details of the computational pipelines producing variant calls, see https://www.nhlbiwgs.org/sequencing-and-data-processing-methods-freeze3a.

### Ancestry estimation

For ancestry estimation, an independent set of SNPs was defined by LD pruning with PLINK 1.9 (flags: --indep-pairwise 100 10 0.05); see www.cog-genomics.org/plink/1.9/ [20]. For each individual, we estimated the %-contribution of each of the five 1000G continental superpopulations to their genetic ancestry using ADMIXTURE v1.3.0 (flags: --supervised 5) [21]. To isolate the influence of genetic ancestry and admixture on PRS variability, we included only individuals with > 95% European (EUR) or African (AFR) ancestry in our downstream analysis. This filter resulted in 1447 EUR ancestry individuals and 239 AFR ancestry individuals considered for the ancestry-specific PRS reproducibility analyses.

### Phasing and imputation

We performed phasing and imputation using three commonly used combinations of algorithms: (1) pre-phasing and imputation with Beagle 5.1 [22] (referred to as Beagle), (2) pre-phasing with Eagle v2.4.1 [23] and imputation with Minimac4 [24] (referred to as Eagle+Minimac), and (3) pre-phasing with SHAPEIT4 [25] and imputation with Minimac4 [24] (referred to as SHAPEIT+Minimac). Minimac and Beagle were run with default settings. For Eagle, we disabled imputation of partially missing genotypes (flags: --noImpMissing) and otherwise applied default settings. With SHAPEIT4, we applied default settings. We used the default genetic map files provided by SHAPEIT4 authors. A total of 37,995,438 variants were imputed six times per condition to ascertain PRS variability. We performed all phasing and imputation steps using the Haplotype Reference Consortium (HRC) as reference [26].

### PRS calculation and ancestry adjustment

PRS calculation was performed using the standard weighted allele-counting approach assuming independent effects across loci using the following equation:

$$ {\mathrm{PRS}}_i=\sum \limits_{j=1}^n{x}_{ij}{\beta}_j $$

where an individual’s (*i*) PRS is the sum of the effect allele dosages (0 ≤ *x*_*ij*_ ≤ 2) for each variant (*j*) included in the score multiplied by its marginal effect size (*β*_j_ - log odds ratio per dosage of effect allele). Percentile ranking was calculated within each matched ancestry members of the study cohort. Specifically, we calculated a 161 SNP score based on a recent analysis identifying 163 confident CAD risk loci [27] with weights based on the latest large-scale CAD meta-GWAS—the CARDIoGRAMplusC4D consortium [28] (PRS_CAD_). The genetic model for non-additive variants was determined as previously described [29, 30]. SNPs and weights are provided in Additional file 1: Table S1. Two variants were excluded from this list, one multi-allelic variant and one variant absent in HRC, resulting in a final 161 SNP score. One hundred twenty-three of these SNPs are not present in the SNP array data and were imputed. Of the 123 imputed SNPs, 105/123 (85.37%) were in LD with a genotyped SNP at *R*^2^ > 0.5, 77/123 (62.60%) at *R*^2^ > 0.8, and 62/123 (50.41%) at *R*^2^ > 0.9 in EUR populations, and for AFR populations 72/123 (58.54%) at *R*^2^ > 0.5, 38/123 (30.89%) at *R*^2^ > 0.8, and 34/123 (27.64%) at *R*^2^ > 0.9. In addition, we investigated the variability of two additional previously published PRS scores for CAD, as well as atrial fibrillation, type 2 diabetes, Alzheimer’s disease, glaucoma, and breast cancer, derived from a variety score construction methodologies as defined in the PGS Catalog (http://www.pgscatalog.org/browse/studies/). These additional PRS models ranged from dozens to millions of risk loci. The breakdown of the number of SNPs and those directly genotyped vs imputed SNPs is provided in Table 1. All statistical analyses were performed in R v3.5.1.

**Table 1:** The breakdown of typed vs imputed SNPs per PRS score

	Number of SNP				Reference
	Total	Found	Typed	Imputed	
**Coronary artery disease (CAD)**					
PRS_CAD_	163	161	38	123	[27]
metaGRS_CAD_	1,745,180	1,736,608	143,724	1,592,884	[31]
GPS_CAD_	6,630,150	6,238,460	567,297	5,671,163	[3]
**Type 2 diabetes (T2D)**					
PRS-GWAS_T2D_ (547)	558	547	66	481	[32]
PRS-GWAS_T2D_ (397)	403	397	29	368	[33]
PRS-GWAS_T2D_ (170487)	171,249	170,487	10,152	160,335	[33]
GPS_T2D_	6,917,436	6,482,889	570,779	5,912,110	[3]
**Breast cancer (BC)**					
PRS-GWAS_BC_ (239)	313	239	34	205	[34]
PRS-GWAS_BC_ (2935)	3820	2941	325	2616	[34]
GPS_BC_	5218	4457	374	4083	[3]
**Atrial fibrillation (Afib)**					
PRS-GWAS_Afib_	166	166	19	147	[35]
GPS_Afib_	6,730,541	6,302,924	570,913	5,732,011	[3]
**Alzheimer’s disease (AD)**					
PRS-GWAS_AD_	29	29	2	27	[36]
**Glaucoma**					
PRS-GWAS_Glaucoma_	2673	2657	224	2433	[37]

### Imputation accuracy calculation

For evaluating the imputation accuracy, we developed a flexible python script to calculate the coefficient of determination (*R*^2^) and other accuracy metrics not used here: *F*-score and imputation quality score [38]. The python script for calculating imputation accuracy is available at https://github.com/TorkamaniLab/imputation_accuracy_calculator.

## Results

We evaluated the variability in PRS scores due to 3 common imputation processes (Beagle, Eagle+Minimac, SHAPEIT+Minimac), using 3 different pre-phasing tools (Beagle, Eagle, SHAPEIT) and 2 different imputation tools (Beagle, Minimac4), relative to a WGS-based gold standard (Fig. 1). Here, we present the variability observed across the combined cohort of 1686 individuals of > 95% European ancestry (1447 individuals) or > 95% African ancestry (229 individuals). Plots separated by genetic ancestry are provided in *Supplemental Figures*—those results are consistent with the combined results presented here, with a greater degree of variability observed in the African ancestry sub-group.

**Fig. 2:**
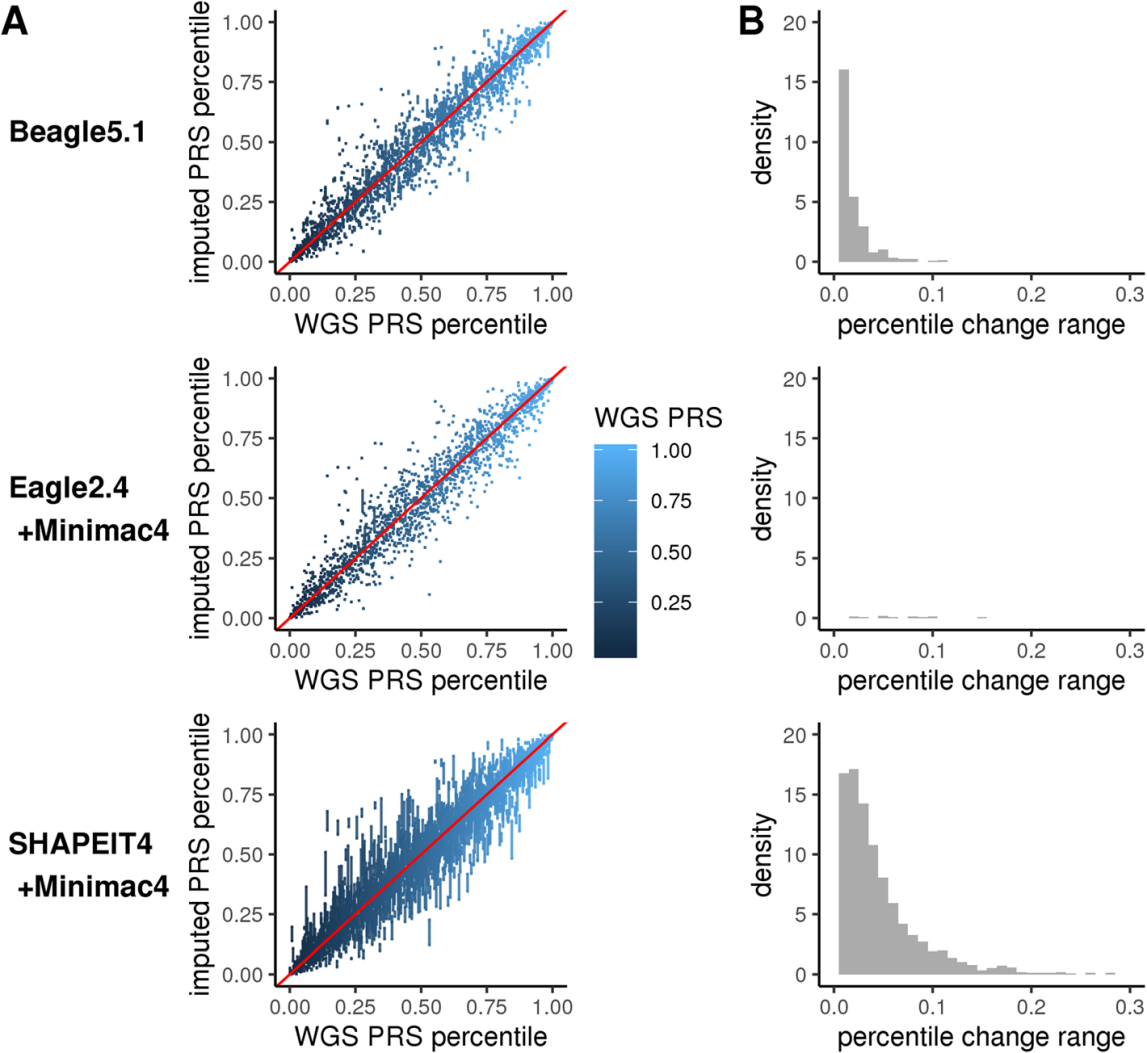
PRS_CAD_ reproducibility. The variability in PRS_CAD_ percentile values as determined by three different imputation processes. **a** Gold standard WGS-based PRS percentile (*x*-axis) vs six replicates of imputation-derived PRS percentiles (*y*-axis). Point darkness depicts WGS-based ranking. **b** Histogram of the absolute score deviations relative to the WGS-based standard. Bin for no change is not shown

**Fig. 3:**
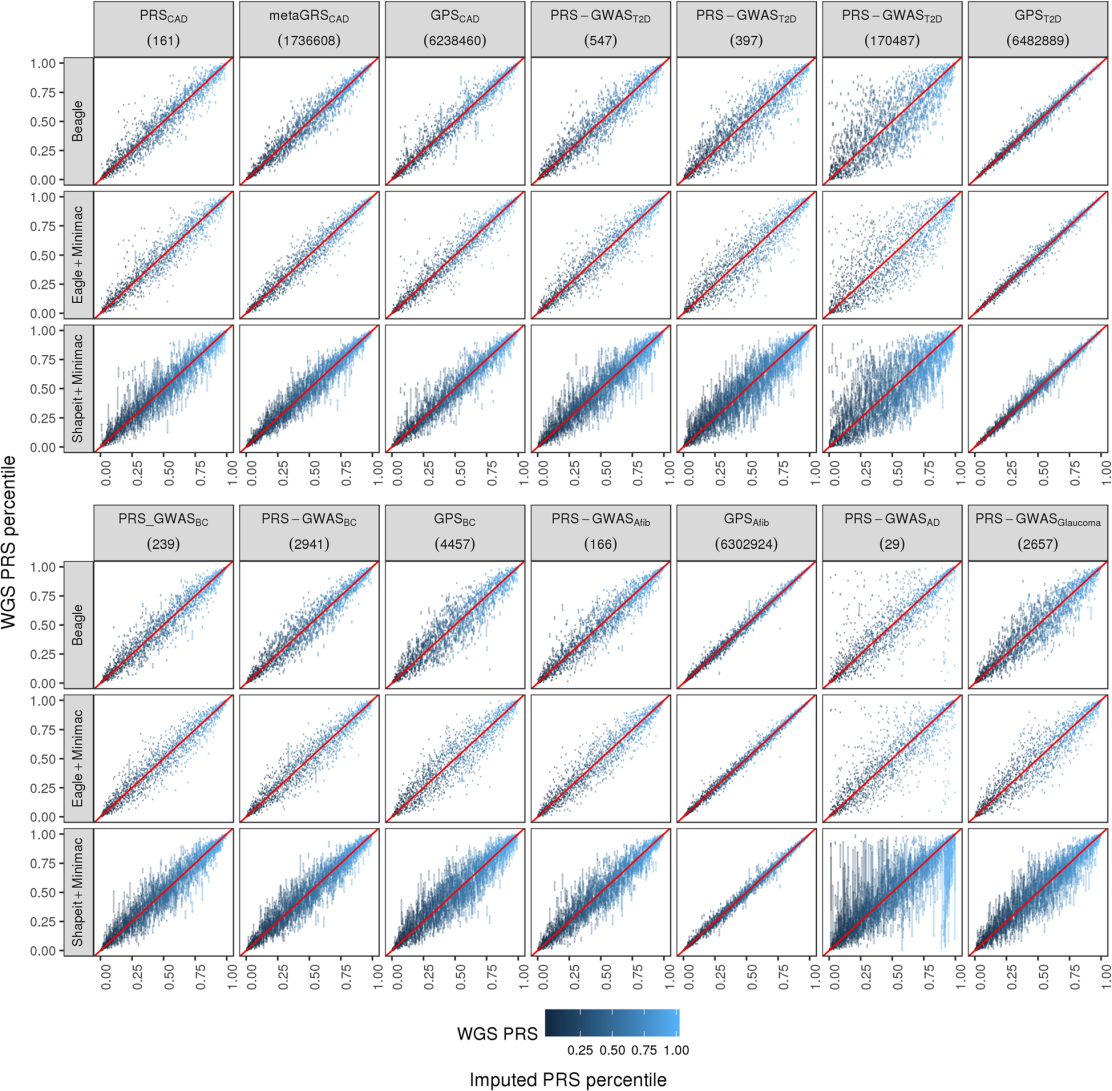
PRS reproducibility. The variability in PRS percentile values as determined by three different imputation processes for 14 different PRSs. Gold standard WGS-based PRS percentile (*x*-axis) vs six replicates of imputation-derived PRS percentiles (*y*-axis). Point darkness depicts WGS-based ranking

For each imputation process, we repeated the PRS calculation process six times starting from the pre-phasing step. Imputation-based PRSs were generally highly correlated with ground truth WGS-based PRSs (average *R*^2^ > 0.9), except for the Alzheimer’s disease PRS and two type 2 diabetes PRSs which consistently showed lower correlation with WGS-based ground truth (Fig. 2a, Additional file 1: Table S2, Fig. S1A-S13A). For each score, the overall level of score dispersion relative to the WGS-based gold standard is approximately equivalent across all three imputation processes when evaluated at the population level—being primarily associated with the genetic architecture being imputed. However, when we evaluate the range of PRS percentile values achieved across the different imputation methods at an individual level, clear differences in PRS stability emerge; SHAPEIT+Minimac leads to the most intra-individual variability, followed by Beagle and Eagle+Minimac (Fig. 2b, Additional file 2: Fig. S1B-S13B). In rare instances (~ 1% of the time), SHAPEIT+Minimac results in a range of > 20 percentile points between the highest and lowest PRS values achieved for an individual (Table 2, Additional file 1: Table S3). Beagle and Eagle+Minimac return relatively stable PRS values, with both methods very rarely (~ 0.1%) resulting in PRS values that change > 10 percentile points between the highest and lowest values achieved for an individual. This algorithm-level variability is observed regardless of the original approach used to derive the PRS and the number of SNPs included in the score—as similar variability is observed for three different CAD risk scores derived using very different strategies and including vastly different numbers of SNPs (Table 2), as well as across all diseases considered (Fig. 3). These results suggest that the pre-phasing step introduces the bulk of the stochasticity in imputation and PRS results. Variability is further increased in individuals of African ancestry (Table 2, Additional file 2: Fig. S14-S27) and is exacerbated for PRSs with worse overall concordance with the WGS-based gold standard.

**Table 2:** The distribution of CAD risk score percentile changes caused by three different imputation processes

Percentile change	Beagle			Eagle+Minimac			SHAPEIT+Minimac		
	EUR	AFR	All	EUR	AFR	All	EUR	AFR	All
**PRS**_**CAD**_ **(161)**									
≤ 1%tile	1247 (86.18%)	163 (68.2%)	1410 (83.63%)	1437 (99.31%)	236 (98.74%)	1673 (99.23%)	259 (17.9%)	5 (2.09%)	264 (15.66%)
> 1%tile	200 (13.82%)	76 (31.8%)	276 (16.37%)	10 (0.69%)	3 (1.26%)	13 (0.77%)	1188 (82.1%)	234 (97.91%)	1422 (84.34%)
> 5%tile	21 (1.45%)	5 (2.09%)	26 (1.54%)	7 (0.48%)	1 (0.42%)	8 (0.47%)	331 (22.87%)	168 (70.29%)	499 (29.6%)
> 10%tile	2 (0.14%)	0 (0%)	2 (0.12%)	1 (0.07%)	0 (0%)	1 (0.06%)	68 (4.7%)	87 (36.4%)	155 (9.19%)
> 20%tile	0 (0%)	0 (0%)	0 (0%)	0 (0%)	0 (0%)	0 (0%)	3 (0.21%)	10 (4.18%)	13 (0.77%)
**metaGRS**_**CAD**_ **(1736608)**									
≤ 1%tile	940 (64.96%)	106 (44.35%)	1046 (62.04%)	1418 (98%)	229 (95.82%)	1647 (97.69%)	153 (10.57%)	13 (5.44%)	166 (9.85%)
> 1%tile	507 (35.04%)	133 (55.65%)	640 (37.96%)	29 (2%)	10 (4.18%)	39 (2.31%)	1294 (89.43%)	226 (94.56%)	1520 (90.15%)
> 5%tile	12 (0.83%)	2 (0.84%)	14 (0.83%)	5 (0.35%)	1 (0.42%)	6 (0.36%)	277 (19.14%)	154 (64.44%)	431 (25.56%)
> 10%tile	2 (0.14%)	0 (0%)	2 (0.12%)	0 (0%)	0 (0%)	0 (0%)	8 (0.55%)	42 (17.57%)	50 (2.97%)
> 20%tile	0 (0%)	0 (0%)	0 (0%)	0 (0%)	0 (0%)	0 (0%)	0 (0%)	0 (0%)	0 (0%)
**GPS**_**CAD**_ **(6238460)**									
≤ 1%tile	1289 (89.08%)	191 (79.92%)	1480 (87.78%)	1429 (98.76%)	233 (97.49%)	1662 (98.58%)	328 (22.67%)	20 (8.37%)	348 (20.64%)
> 1%tile	158 (10.92%)	48 (20.08%)	206 (12.22%)	18 (1.24%)	6 (2.51%)	24 (1.42%)	1119 (77.33%)	219 (91.63%)	1338 (79.36%)
> 5%tile	26 (1.8%)	0 (0%)	26 (1.54%)	2 (0.14%)	0 (0%)	2 (0.12%)	239 (16.52%)	69 (28.87%)	308 (18.27%)
> 10%tile	12 (0.83%)	0 (0%)	12 (0.71%)	0 (0%)	0 (0%)	0 (0%)	56 (3.87%)	9 (3.77%)	65 (3.86%)
> 20%tile	2 (0.14%)	0 (0%)	2 (0.12%)	0 (0%)	0 (0%)	0 (0%)	6 (0.41%)	1 (0.42%)	7 (0.42%)

Columns indicate the number and percentage of subjects in the population with percentile change in their PRS. *EUR* European, *AFR* African

**Fig. 4:**
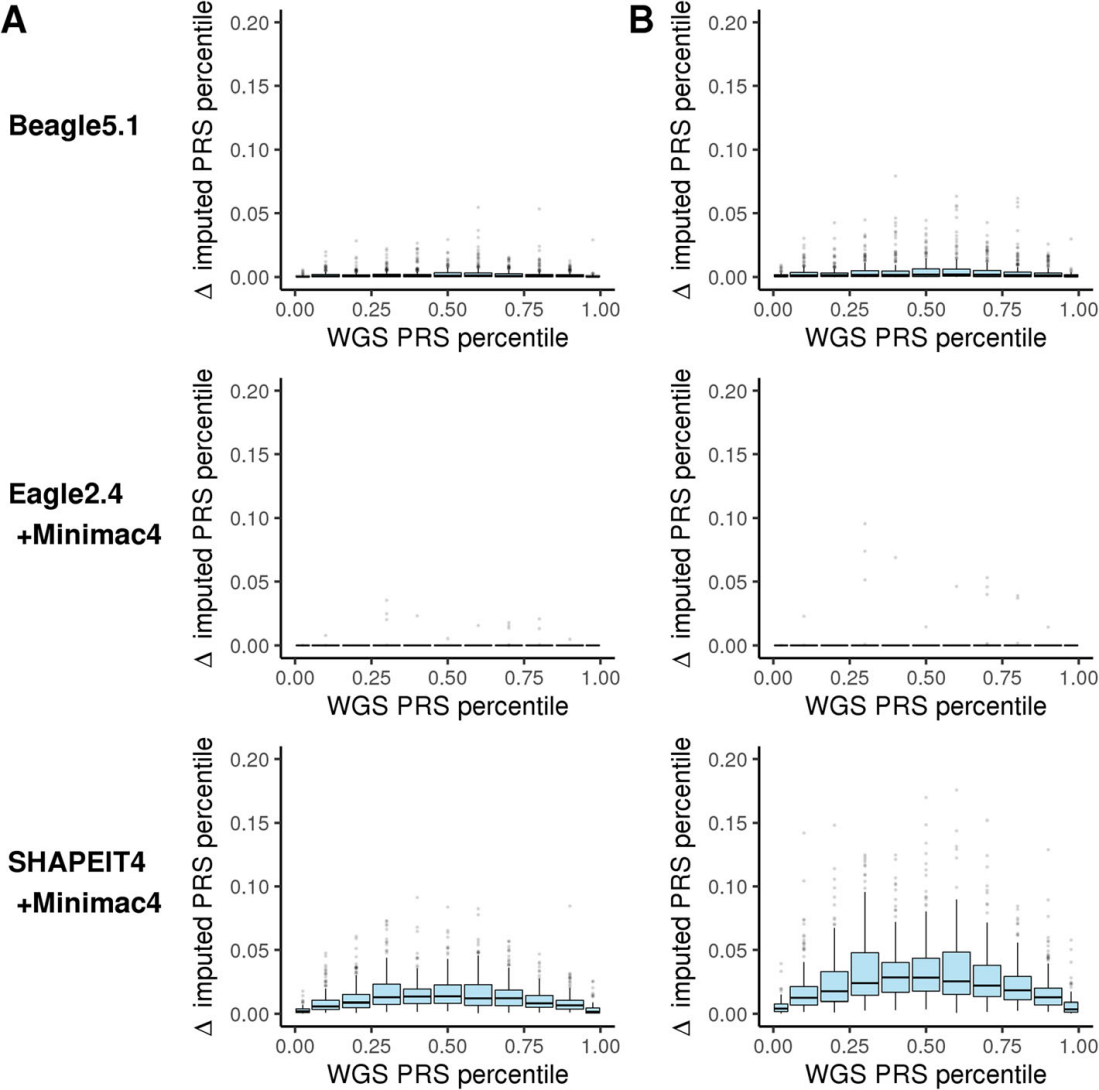
PRS_CAD_ variability as a function of PRS bin. The degree of variability in PRS percentile as a function of the expected WGS-based PRS tier across three different imputation processes. **a** Average absolute deviation per individual relative to their WGS-based gold standard. **b** Maximum absolute deviation per individual relative to the WGS-based gold standard. Box plots depict the interquartile range as is standard

Next, we determined which individuals are most likely to be affected by imputation-induced PRS score variability. As expected, the variability of the PRS percentile is greatest in the middle of the distribution and lowest at the tails of the distribution when quantified as the average absolute deviation (Fig. 4a, Additional file 1: Fig. S28A-S40A) and maximum absolute deviation (Fig. 4b, Additional file 1: Fig. S28B-S40B) in PRS percentile per person. This is true for both European and African ancestry individuals (Additional file 1: Fig. S41-S54). This is the expected behavior given raw score increments due to imputation variability would be uniform across the normal distribution of the raw PRS, but those equal-sized raw score increments result in larger percentile rank changes in the middle of the distribution vs the tails. Thus, the largest percentile rank changes occur in individuals where the utility implications are least influenced by that change (individuals who are overall at average risk and whose score fluctuates within the average risk range). While relatively large percentile changes may not change the utility conclusion for average risk individuals, those changes would likely lead to a perceived change in risk. In addition, we observe some significant variability, > 5 percentile changes in score, at the tails of the risk distribution. Smaller percentile changes at the extreme ends of the risk distribution can influence both the clinical implications as well as perceptions of risk [3]. Overall, using typical quintile thresholds for defining high (top 20%) vs intermediate (middle 60%) vs low (bottom 20%) risk individuals, we observe that run-to-run imputation variability can lead to differing utility conclusions in < 1% of individuals when using Beagle and Eagle+Minimac, whereas interpretation variability can exceed 5% of individuals for SHAPEIT+Minimac (Table 3, Additional file 1: Table S4). When evaluated using more refined risk tier thresholds for high-risk individuals, the rate of re-classification increases further as the distance between risk tier thresholds shrinks, in some cases exceeding re-classification of 20% of individuals placed in a more narrowly defined top/bottom 5% percentile threshold (Table 4, Additional file 1: Table S5).

**Table 3:** The rate of risk tier re-classification due to imputation variability using quintile-based cutoffs

Risk tier	Beagle			Eagle+Minimac			SHAPEIT+Minimac		
	< 20%tile	20–80%tile	> 80%tile	< 20%tile	20–80%tile	> 80%tile	< 20%tile	20–80%tile	> 80%tile
**PRS**_**CAD**_ **(161)**									
< 20%tile	19.89%	0.09%	0%	19.99%	0%	0%	19.27%	0.72%	0%
20–80%tile	0.04%	59.90%	0.09%	0%	60.01%	0.01%	0.48%	58.88%	0.64%
> 80%tile	0%	0.10%	19.90%	0%	0.01%	19.98%	0%	0.48%	19.52%
**metaGRS**_**CAD**_ **(1736608)**									
< 20%tile	19.88%	0.11%	0%	19.98%	0.01%	0%	19.47%	0.51%	0%
20–80%tile	0.29%	59.59%	0.13%	0.01%	60%	0%	0.51%	58.95%	0.54%
> 80%tile	0%	0.09%	19.92%	0%	0.01%	19.99%	0%	0.50%	19.50%
**GPS**_**CAD**_ **(6238460)**									
< 20%tile	19.85%	0.14%	0%	19.96%	0.02%	0%	19.57%	0.42%	0%
20–80%tile	0.08%	59.76%	0.17%	0.03%	60%	0%	0.59%	58.93%	0.48%
> 80%tile	0%	0.13%	19.88%	0%	0%	19.99%	0%	0.56%	19.44%

The rate of re-classification into low risk (< 20th percentile), intermediate risk (20–80th percentile), and high risk (> 80th percentile) due to phasing and imputation variability across all individuals included in this study (1447 EURs and 239 AFRs combined). Columns indicate the average individual-level imputation-based risk tier. Rows indicate the process-level imputation-based risk tier

**Table 4:** The rate of risk tier re-classification for high risk individuals due to phasing and imputation variability

Risk tier	Beagle	Eagle+Minimac	SHAPEIT+Minimac
**PRS**_**CAD**_ **(161)**			
< 5%tile	1.19% (− 1.13–3.51)	0% (0–0)	15.66% (7.84–23.48)
< 10%tile	2.96% (0.4–5.51)	0% (0–0)	10.12% (5.56–14.68)
< 15%tile	1.59% (0.04–3.13)	0% (0–0)	9.96% (6.26–13.67)
< 20%tile	0.89% (− 0.11–1.9)	0% (0–0)	9.91% (6.7–13.12)
> 80%tile	1.78% (0.37–3.19)	0.3% (− 0.28–0.88)	10% (6.81–13.19)
> 85%tile	1.98% (0.26–3.69)	0% (0–0)	12.7% (8.59–16.81)
> 90%tile	1.19% (− 0.45–2.83)	0% (0–0)	17.54% (11.84–23.24)
> 95%tile	4.71% (0.2–9.21)	0% (0–0)	15.85% (7.95–23.76)
**metaGRS**_**CAD**_ **(1736608)**			
< 5%tile	6.98% (1.59–12.36)	1.19% (− 1.13–3.51)	18.6% (10.38–26.83)
< 10%tile	2.37% (0.07–4.66)	0% (0–0)	6.67% (2.86–10.47)
< 15%tile	2.37% (0.5–4.25)	0.79% (− 0.3–1.88)	12.94% (8.82–17.06)
< 20%tile	3.82% (1.79–5.86)	0.3% (− 0.28–0.88)	8.9% (5.86–11.94)
> 80%tile	2.66% (0.95–4.38)	0% (0–0)	9.76% (6.6–12.93)
> 85%tile	4.72% (2.12–7.33)	0% (0–0)	12.11% (8.11–16.11)
> 90%tile	6.43% (2.76–10.11)	0% (0–0)	6.06% (2.42–9.7)
> 95%tile	2.38% (− 0.88–5.64)	0% (0–0)	9.52% (3.25–15.8)
**GPS**_**CAD**_ **(6238460)**			
< 5%tile	5.88% (0.88–10.88)	0% (0–0)	13.25% (5.96–20.55)
< 10%tile	2.38% (0.08–4.69)	0.59% (− 0.56–1.75)	7.78% (3.72–11.85)
< 15%tile	0.79% (− 0.3–1.88)	0% (0–0)	9.92% (6.23–13.61)
< 20%tile	1.49% (0.19–2.78)	0.3% (− 0.28–0.88)	10% (6.81–13.19)
> 80%tile	2.37% (0.75–3.99)	0% (0–0)	8.33% (5.38–11.29)
> 85%tile	1.97% (0.26–3.68)	0% (0–0)	6.48% (3.41–9.55)
> 90%tile	2.37% (0.07–4.66)	0% (0–0)	5.99% (2.39–9.59)
> 95%tile	1.18% (− 1.12–3.47)	0% (0–0)	10.59% (4.05–17.13)

The rate of individuals re-classified from high risk tiers due to imputation variability across all individuals included in this study (1447 EURs and 239 AFRs combined). An individual is considered re-classified if at least two imputation replicates produce a different risk tier than the WGS-based ground truth. Values in parenthesis present the 95% confidence interval

**Fig. 5:**
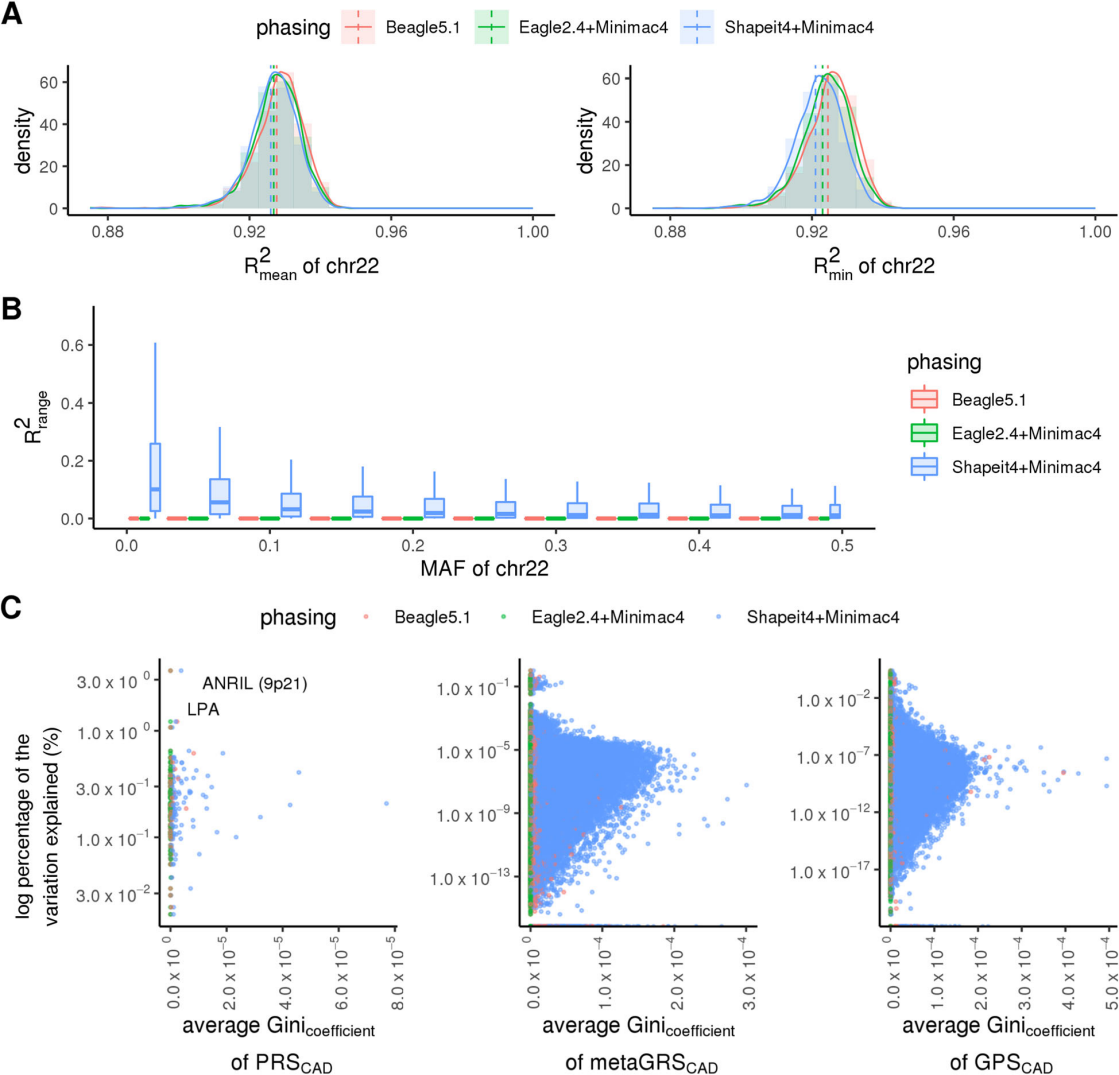
Imputation accuracy relative to WGS-based gold standard expressed as *R*^2^. **a** Distribution of *R*^2^ values per individual for chromosome 22—average *R*^2^ (left) and minimum *R*^2^ (right) for 6 replicate imputation runs. Dashed vertical lines indicate the median values. **b** Range of *R*^2^ values achieved per minor allele frequency (MAF) bin for chromosome 22. **c** Scatterplot of percent variation explained (log-scale) per SNP for CAD PRSs (left: PRS_CAD_, middle: metaGRS_CAD_, right: GPS_CAD_) vs imputation variability as defined by the Gini coefficient of SNP imputation results

Finally, we sought to determine whether imputation score variability was reflective of any differences in overall score accuracy at the genotype level relative to gold standard WGS-based genotypes. We compared the mean and minimum *R*^2^ values between imputed and gold standard genotype dosages per imputation process and replicate for all SNPs on chromosome 22 (Fig. 5a, b). Overall, the three approaches demonstrate equivalent average accuracy relative to WGS-based genotypes *R*^2^ = ~ 0.925 (Fig. 5a left). However, when restricting our view to the minimum accuracy achieved per individual by each process, a left shift in minimum accuracy is observed for SHAPEIT+Minimac (Fig. 5a right) (*p* value < 2e−16 by paired Wilcoxon). As expected, the range of accuracy achieved is larger for rare genetic variants though variants across the entire minor allele frequency spectrum contribute to this variability in accuracy (Fig. 5b). This individual-level imputation variability is masked by the approximately equivalent average accuracy when evaluated at a population level (Fig. 5a). To dissect the source of variability further, we examined the potential contribution of each SNP to the overall degree of score variability, which is related to the consistency of its imputation (captured by the Gini coefficient of imputation results, which ranges from 0 to 1 where a value of 0 corresponds to completely consistent imputation results) and its relative contribution to the overall score (captured by percent variability explained, which is further a function of the SNP weight and its allele frequency variance) (Fig. 5c). As expected, the vast majority of SNPs show low variability as captured by a low Gini coefficient. For those SNPs contributing to score variability, we observe both single SNP variability events (top left plot quadrant—high weight variants with moderate levels of imputation variability like *LPA* and *ANRIL* for CAD and *APOE* for Alzheimer’s disease) as well as the accumulation of errors across multiple SNPs (bottom right plot quadrant—several low weight variants with a higher degree of imputation variability). When applied across a large population, where rare computational events will accumulate in a small subset of individuals simply by random chance, these fluctuations can lead to dramatic PRS differences when the score is updated, even when no changes are made to the underlying PRS model or input genotype data.

## Discussion

Several initiatives are ongoing to evaluate the behavioral and health outcomes associated with PRS reporting [39–43]. PRSs will continue to be updated over time as GWASs expand in size, additional risk loci are identified, and more sophisticated score calculation and variant weighting schemes are introduced. While updating our MyGeneRank CAD score (https://mygenerank.scripps.edu/blog/post/mgr-new-update), we sought to identify a computational process that would lead to the greatest degree of PRS stability and accuracy in the face of a score that is expected to evolve over time. In most cases, the degree of score fluctuation resulting from imputation variability is small and does not change the interpretation of the result. Most individuals remain in the same broad risk tier, and the degree of score fluctuation is reduced in the tails of the distribution where disease risk implications are most useful. However, upon systematic investigation of the individual-level run-to-run variability of PRS score generation reported here, we observe rare instances where a score update can lead to a substantial fluctuation in PRS without any change to the underlying genetic model. The magnitude of these fluctuations is not obviously related to the method used to define the score nor the number of SNPs contained within a score. Our observations suggest that imputation variability across all components of a score contributes to score fluctuations at an individual level. Though clearly, SNPs with the highest weights contribute most strongly and consistently to score fluctuations under modest but generally accepted levels of imputation variability. In a population screening setting, both infrequent SNP variability events for singular high weight SNPs or the rare accumulation of run-to-run imputation fluctuations across low weight SNPs will return rare score results for some individuals. Given that some of the promise of PRSs in practice relates to prioritization of preventative behaviors, even minor fluctuations in score may reduce the perceived confidence of the score by the recipient—despite the fact that the overall risk implications are unchanged. Thus, we suggest that either deterministic imputation processes should be favored or stochastic imputation processes could be run multiple times in order to select the most common result. We expect score accuracy is equivalent under these two scenarios.

## Conclusions

Overall, given the dynamic and evolving nature of PRSs and their purported influence on health decision-making, we suggest that methods for the effective forecasting of score changes should be developed. Besides the score variability introduced by computational processes, PRSs also evolve over time depending upon the underlying genetic architecture and size of GWASs currently executed. A projection of the remaining risk loci and their likely effect sizes can be used to project the likely future state of an individual’s PRS [44] to provide them some idea of the uncertainty in their risk estimate and to set expectations for the level of score change that might be expected upon update. To accomplish this, all technical sources of score variability must be addressed and minimized in order to allow for an accurate projection of PRS uncertainty due to undiscovered risk loci. During our CAD score update, we found that both computational stochasticity and changes to the underlying genetic model contribute to PRS variability. Score variability due to imputation was expected to be a more prominent issue for PRSs with a small number of contributing variants (like our original 57 SNP MyGeneRank CAD score) and was expected to diminish in impact for PRSs composed of many SNPs, i.e., imputation variability should regress to the mean with high SNP counts. However, our results suggest that significant (and equivalent) score variability exists even in scores composed of thousands of SNPs, suggesting that imputation variability in the most highly weighted SNPs must be carefully addressed in all PRS deployments. Increased imputation variability in underrepresented populations will further exacerbate this issue and potentially contribute to health disparities as a result of PRS deployment [45]. This variability highlights a pressing need for expanded and diverse imputation reference panels. We expect score variability is further exacerbated for admixed individuals especially when considering the additional variability that might be introduced upon matching an admixed individual to a genetically matched reference population to derive relative score rankings. Thus, while updating genetic models themselves introduces the bulk of population-level score variability, it is important to control for computational variability so that score changes and projections due to improvements to PRS knowledgebases can be deployed and communicated with confidence.

## Supplementary Information

**Additional file 1:** **Supplementary Tables S1-S5.** Components of PRS_CAD_ (Table S1). Correlation of imputed-based PRSs and WGS-based ground truth (Table S2). The distribution of risk score percentile rank changes caused by three different imputation processes for other diseases (Table S3). The rate of re-classification due to imputation variability at different quintile-based risk tiers (Table S4). The rate of re-classification due to imputation variability for high-risk tiers (Table S5).

**Additional file 2:** **Supplemental Fig. S1-S13.** PRS Reproducibility Across Diseases and Score Derivation Methods. **Supplemental Fig. S14-S27.** PRS Reproducibility Across Diseases and Score Derivation Methods by Ancestry. **Supplemental Fig. S28-S40.** PRS Variability Across Diseases and Score Derivation Methods as a Function of Percentile Bin. **Supplemental Fig. S41-S54.** PRS Variability Across Diseases and Score Derivation Methods as a Function of Percentile Bin by Ancestry. **Supplemental Fig. S55.** SNP level variability by Score Impact Across Diseases and Score Derivation Methods.

## Data Availability

The data that support the findings of this study are available from dbGAP, but restrictions apply to the availability of these data, which were used under ethics approval for the current study, and so are not openly available to the public. Data are however available from the authors upon reasonable request and with permission of dbGAP. The ARIC cohort dataset used in this study is available via dbGAP under accession phs000280.v6.p1; https://www.ncbi.nlm.nih.gov/projects/gap/cgi-bin/study.cgi?study_id=phs000280.v6.p1 [46]. The python script for calculating imputation accuracy is available at https://github.com/TorkamaniLab/imputation_accuracy_calculator [47].

## Abbreviations

1000G: 1000 Genomes Phase3 v5
AFR: African
ARIC: Atherosclerosis Risk in Communities
CAD: Coronary artery disease
EUR: European
GWAS: Genome-wide association study
HRC: Haplotype Reference Consortium
LD: Linkage disequilibrium
PRS: Polygenic risk score
SNP: Single-nucleotide polymorphism
WGS: Whole genome sequencing
