# Supplementary material for: Genotype imputation and variability in polygenic risk score estimation
Source: Genome Med. 2020 Nov 23;12:100. doi: 10.1186/s13073-020-00801-x (PMC7682022; doi:10.1186/s13073-020-00801-x)
Supplement: Supplementary file 2 — Additional file 2: Supplemental Fig. S1-S13. PRS Reproducibility Across Diseases and Score Derivation Methods. Supplemental Fig. S14-S27. PRS Reproducibility Across Diseases and Score Derivation Methods by Ancestry. Supplemental Fig. S28-S40. PRS Variability Across Diseases and Score Derivation Methods as a Function of Percentile Bin. Supplemental Fig. S41-S54. PRS Variability Across Diseases and Score Derivation Methods as a Function of Percentile Bin by Ancestry. Supplemental Fig. S55. SNP level variability by Score Impact Across Diseases and Score Derivation Methods. [file 13073_2020_801_MOESM2_ESM.pdf]

Genotype Imputation and Variability in Polygenic Risk Score Estimation

Shang-Fu Chen[1,2], Raquel Dias[1,2], Doug Evans[1,2], Elias L. Salfati[1,2], Shuchen Liu[1,2], Nathan E Wineinger[1,2], Ali Torkamani[1,2]

[1] Scripps Research Translational Institute, La Jolla, California, 92037, USA

[2] Department of Integrative Structural and Computational Biology, Scripps Research, La Jolla, California, 92037, USA

Corresponding Author: atorkama@scripps.edu

Fig S1. metaGRS$_{CAD}$ Reproducibility.

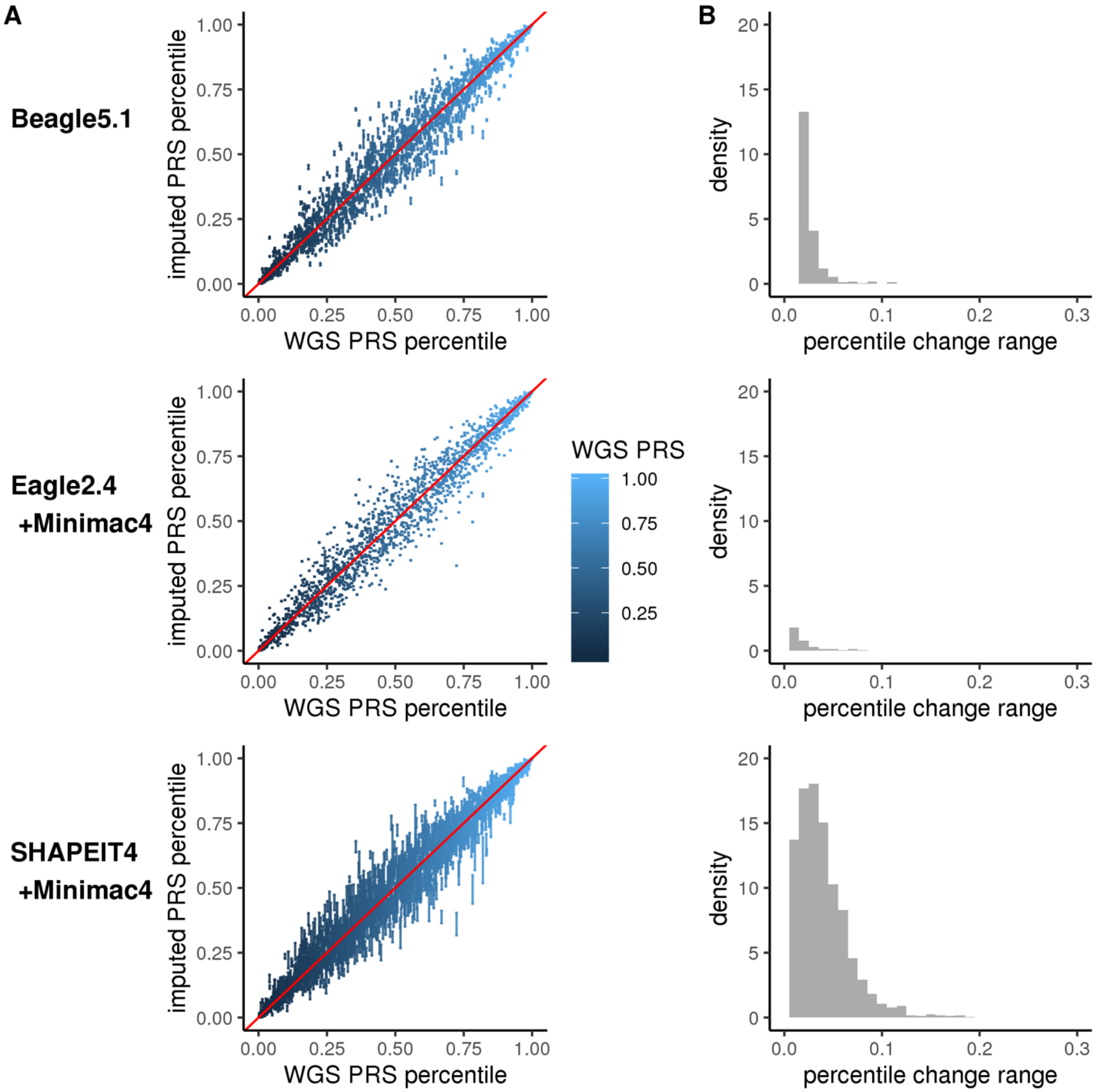

The variability in metaGRS$_{CAD}$ percentile values as determined by three different imputation processes. **A.** Gold standard WGS-based PRS percentile (x-axis) vs six replicates of imputation derived PRS percentiles (y-axis). Point darkness depicts point density for overplotting. **B.** Histogram of the absolute score deviations relative to the WGS-based standard. Note, bin for no change is not shown.

Fig S2. GPS_{CAD} Reproducibility.

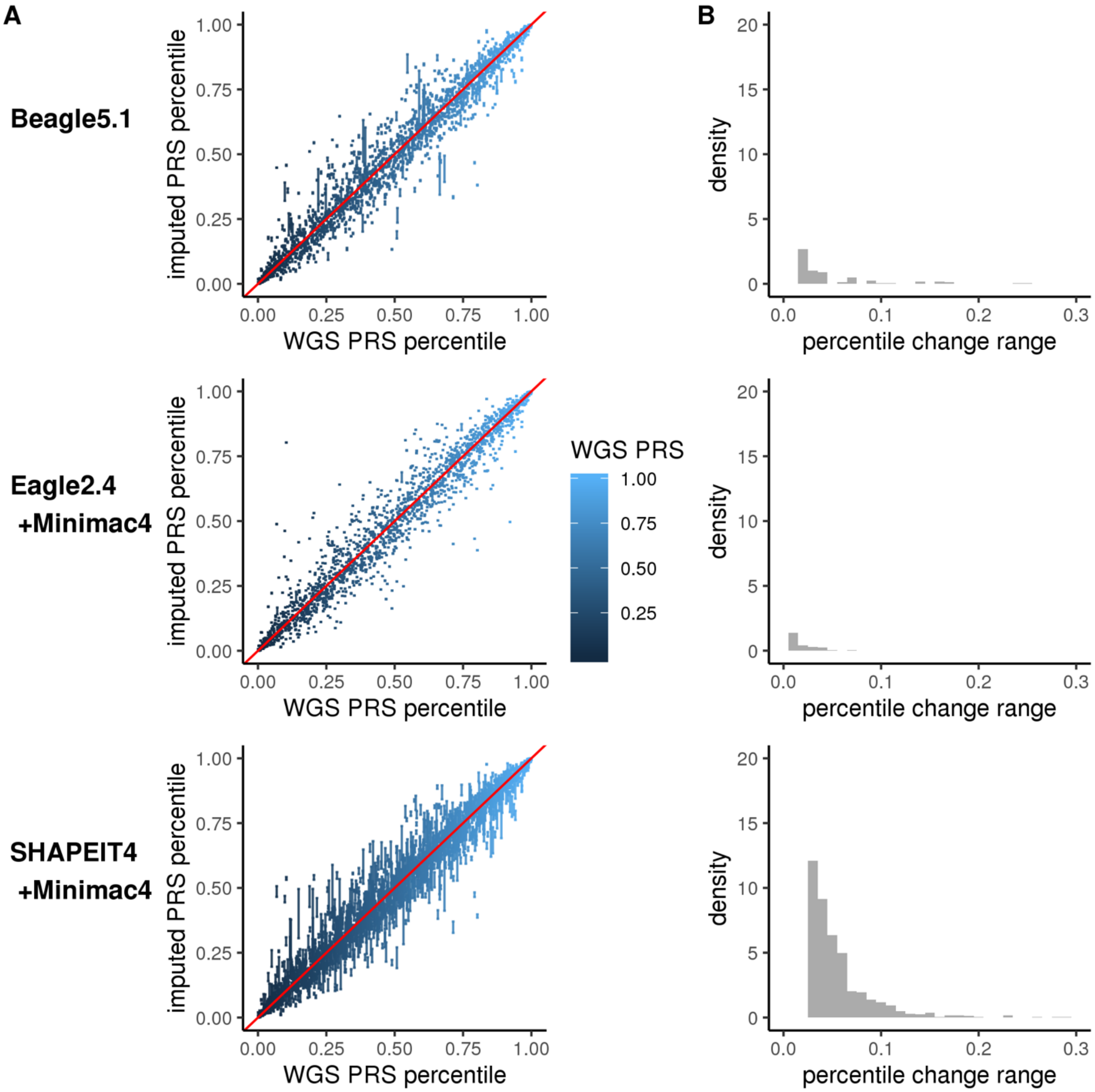

The variability in GPS_{CAD} percentile values as determined by three different imputation processes. **A.** Gold standard WGS-based PRS percentile (x-axis) vs six replicates of imputation derived PRS percentiles (y-axis). Point darkness depicts point density for overplotting. **B.** Histogram of the absolute score deviations relative to the WGS-based standard. Note, bin for no change is not shown.

Fig S3. PRS-GWAS$_{T2D}$ (547) Reproducibility.

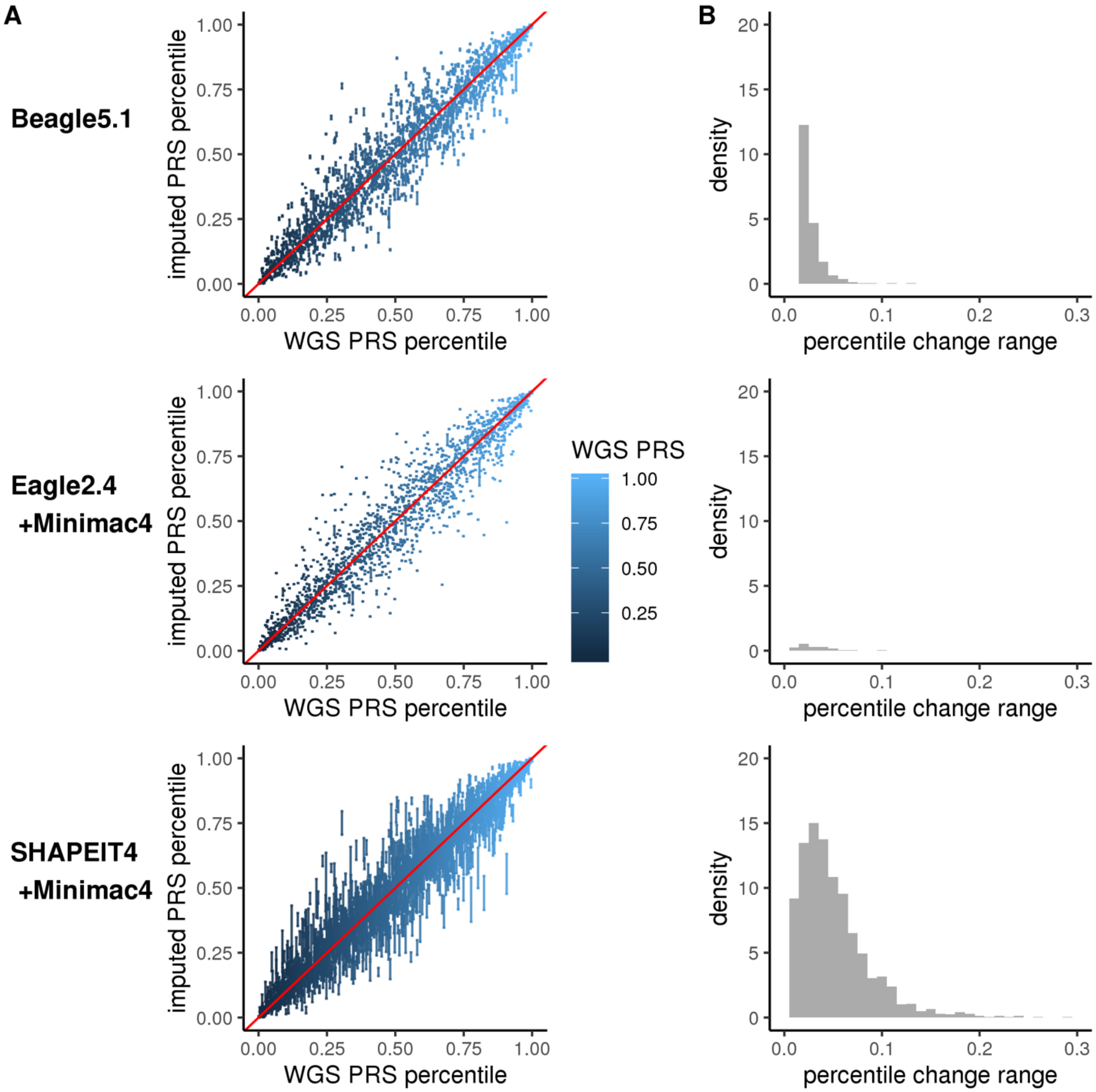

The variability in PRS-GWAS$_{T2D}$ (547) percentile values as determined by three different imputation processes. **A.** Gold standard WGS-based PRS percentile (x-axis) vs six replicates of imputation derived PRS percentiles (y-axis). Point darkness depicts point density for overplotting. **B.** Histogram of the absolute score deviations relative to the WGS-based standard. Note, bin for no change is not shown.

Fig S4. PRS-GWAS$_{T2D}$ (397) Reproducibility.

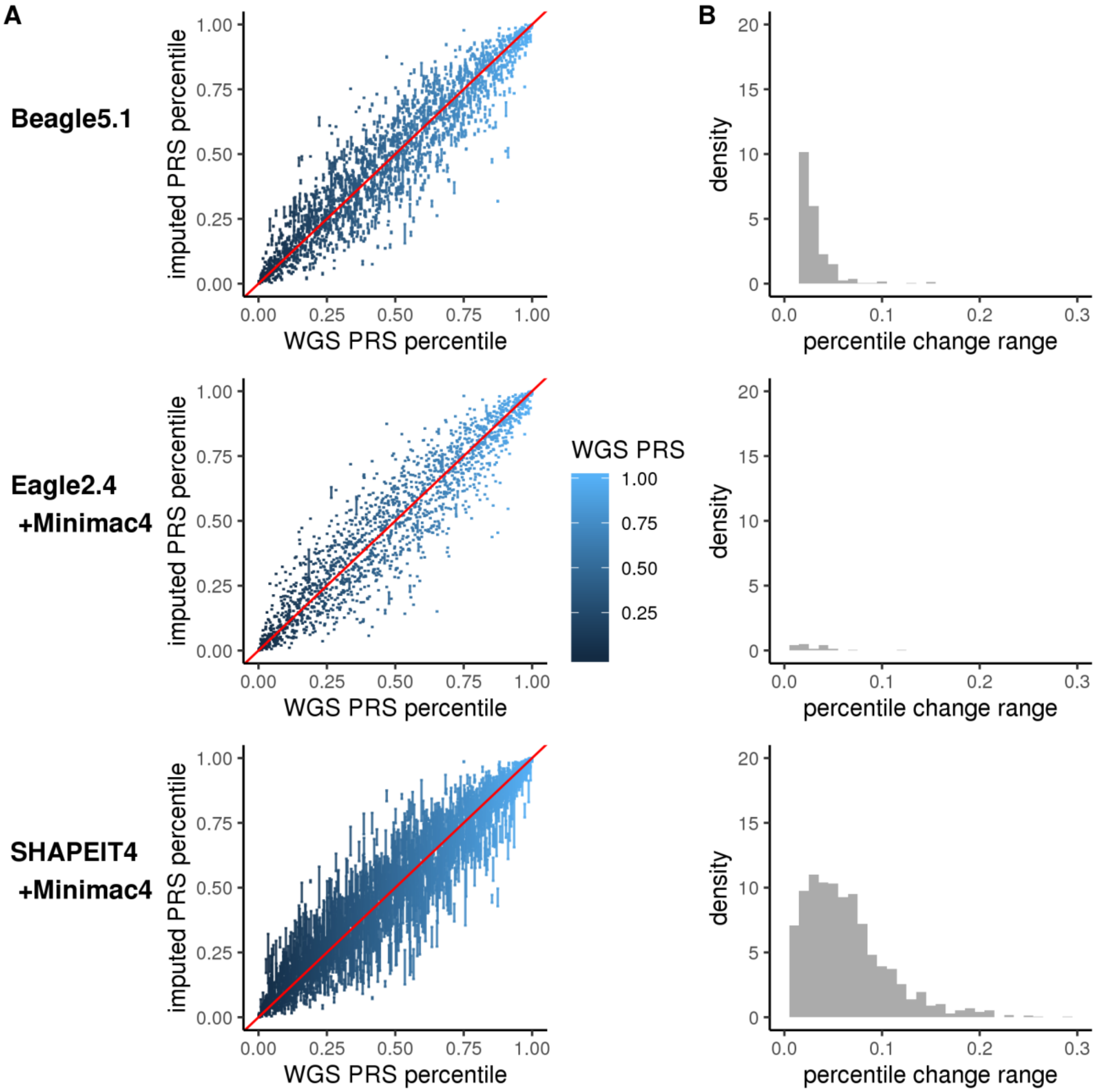

The variability in PRS-GWAS$_{T2D}$ (397) percentile values as determined by three different imputation processes. **A.** Gold standard WGS-based PRS percentile (x-axis) vs six replicates of imputation derived PRS percentiles (y-axis). Point darkness depicts point density for overplotting. **B.** Histogram of the absolute score deviations relative to the WGS-based standard. Note, bin for no change is not shown.

Fig S5. PRS-GWAS$_{T2D}$ (170487) Reproducibility.

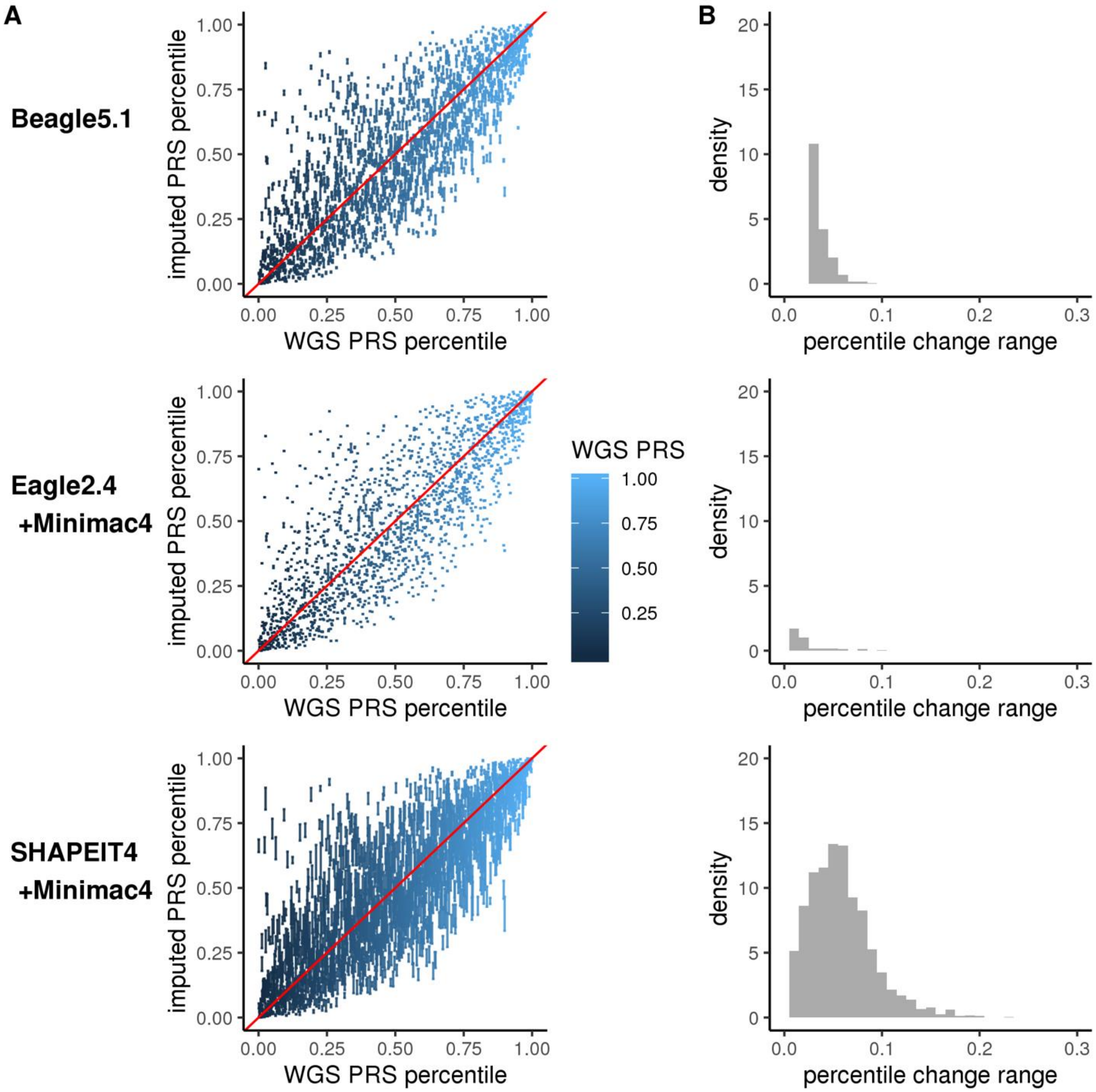

The variability in PRS-GWAS$_{T2D}$ (170487) percentile values as determined by three different imputation processes. **A.** Gold standard WGS-based PRS percentile (x-axis) vs six replicates of imputation derived PRS percentiles (y-axis). Point darkness depicts point density for overplotting. **B.** Histogram of the absolute score deviations relative to the WGS-based standard. Note, bin for no change is not shown.

Fig S6. GPS_{T2D} Reproducibility.

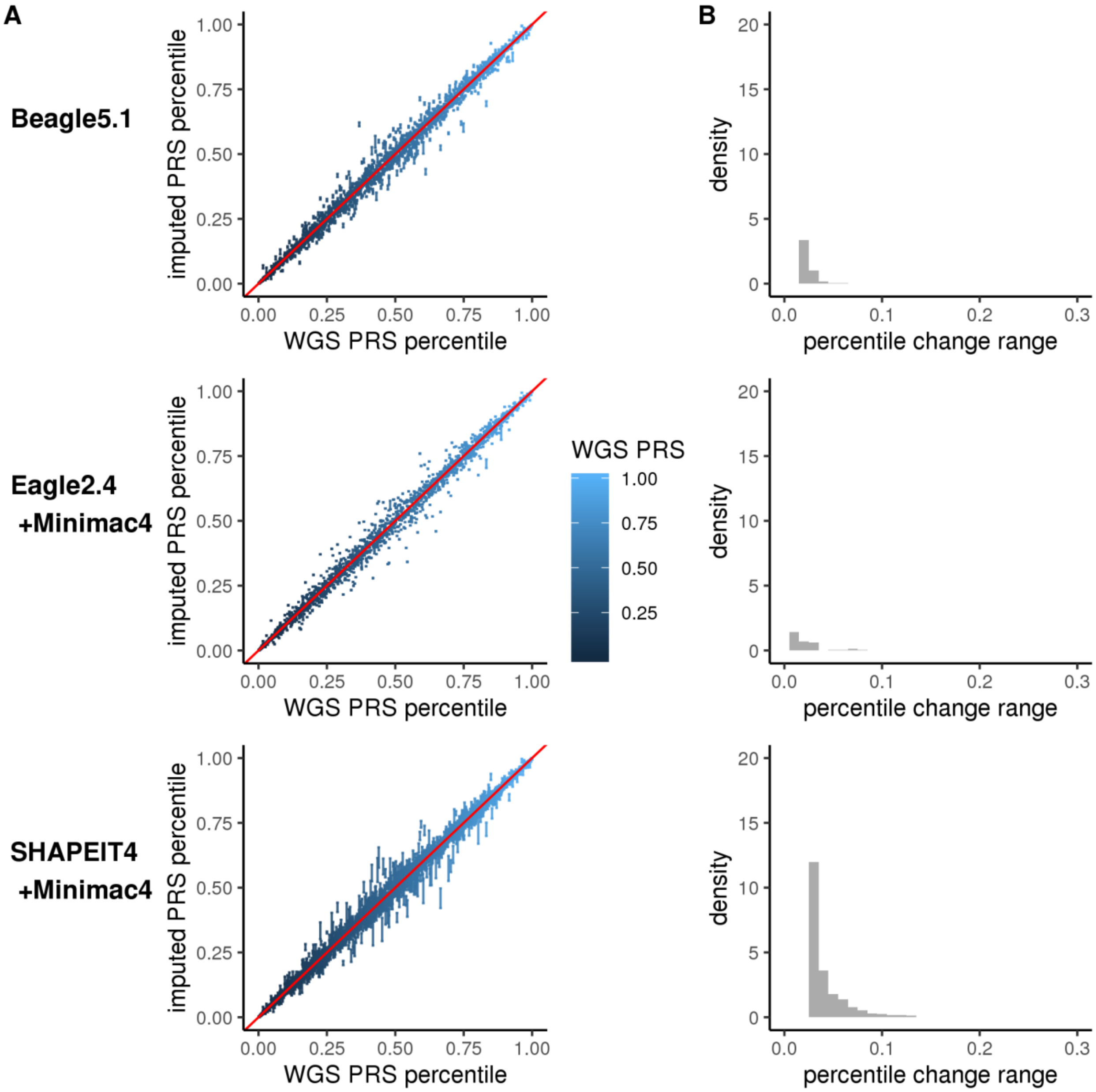

The variability in GPS_{T2D} percentile values as determined by three different imputation processes. **A.** Gold standard WGS-based PRS percentile (x-axis) vs six replicates of imputation derived PRS percentiles (y-axis). Point darkness depicts point density for overplotting. **B.** Histogram of the absolute score deviations relative to the WGS-based standard. Note, bin for no change is not shown.

Fig S7. PRS-GWAS$_{BC}$ (239) Reproducibility.

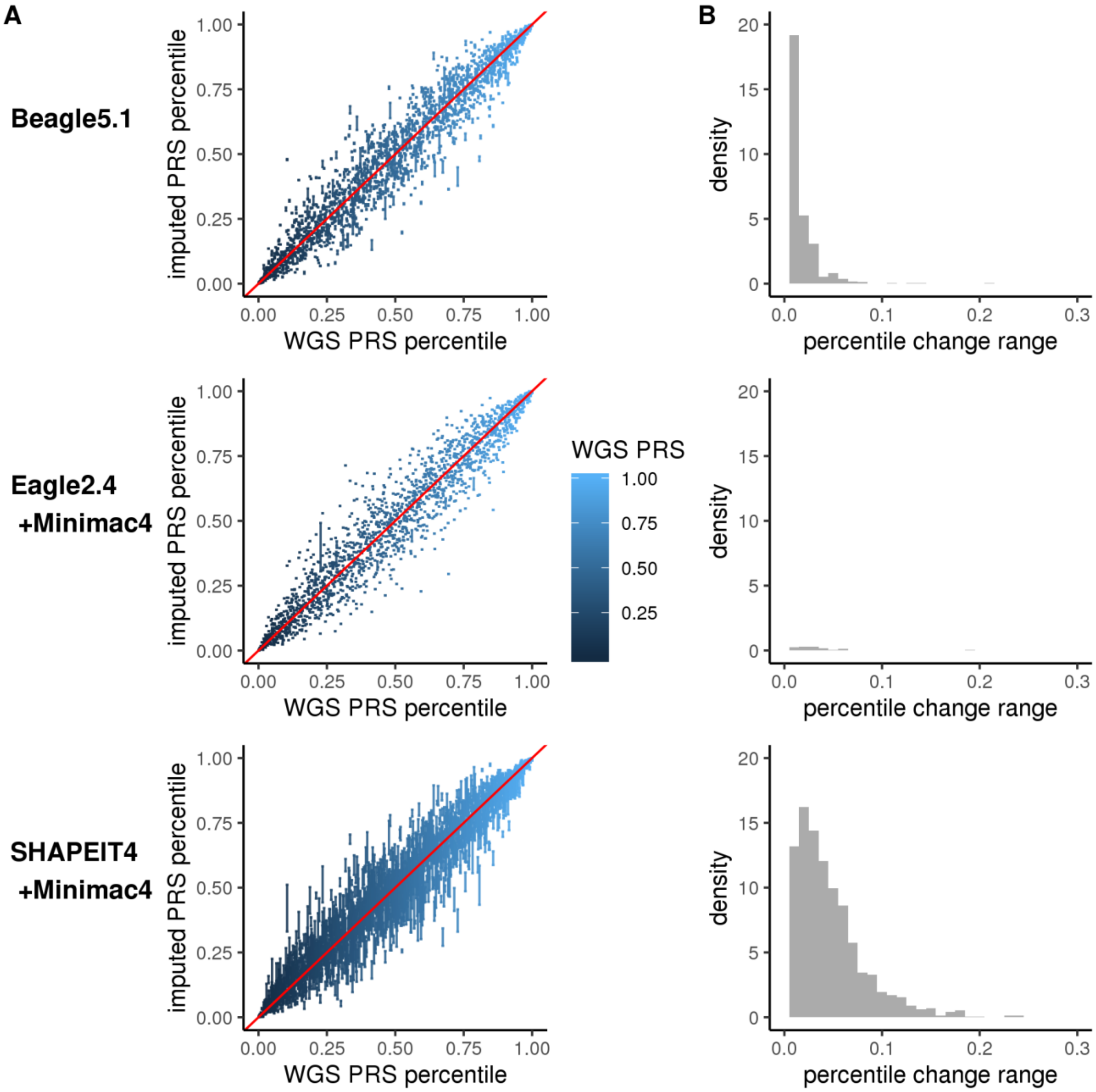

The variability in PRS-GWAS$_{BC}$ (239) percentile values as determined by three different imputation processes. **A.** Gold standard WGS-based PRS percentile (x-axis) vs six replicates of imputation derived PRS percentiles (y-axis). Point darkness depicts point density for overplotting. **B.** Histogram of the absolute score deviations relative to the WGS-based standard. Note, bin for no change is not shown.

Fig S8. PRS-GWAS$_{BC}$ (2935) Reproducibility.

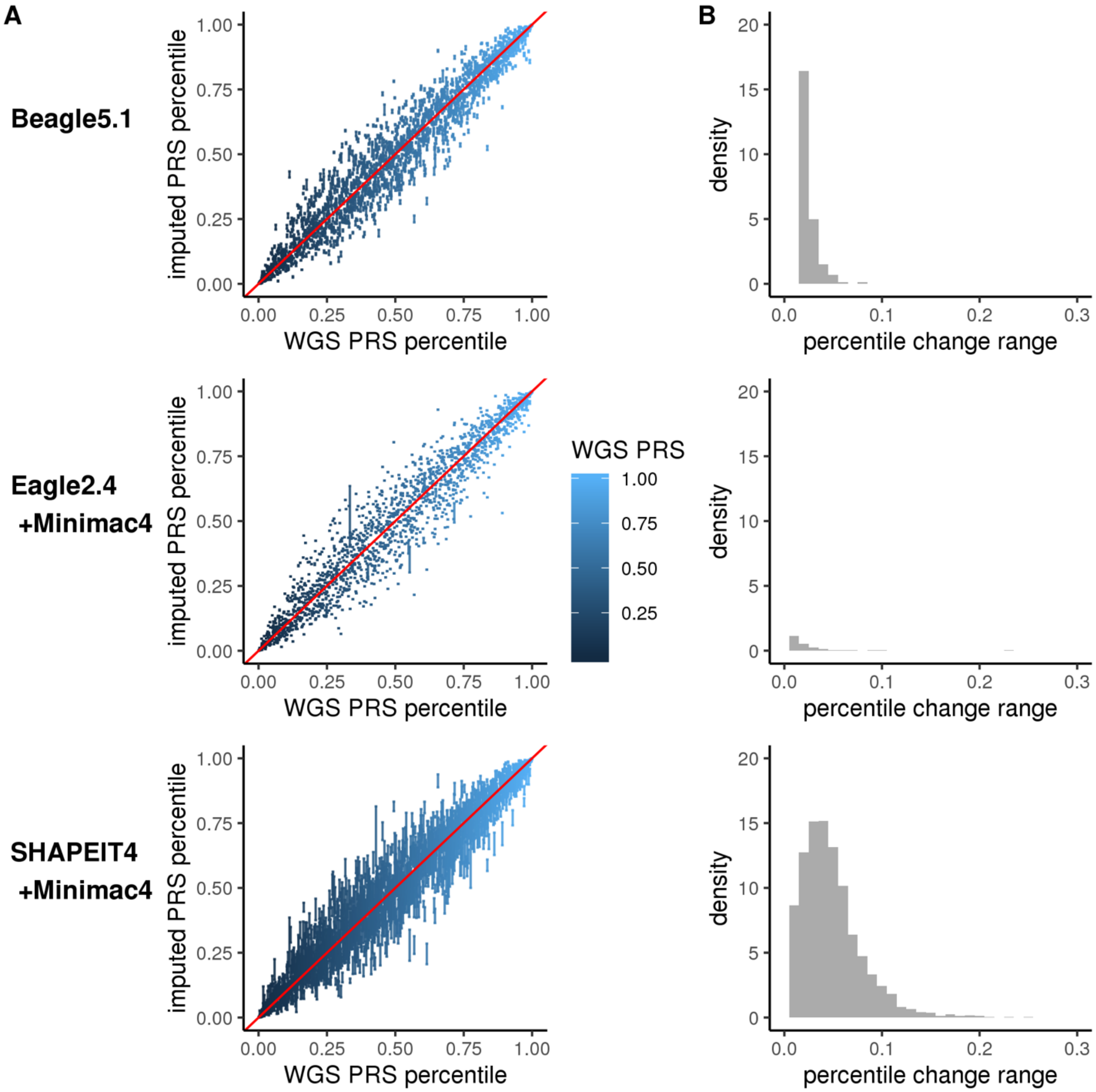

The variability in PRS-GWAS$_{BC}$ (2935) percentile values as determined by three different imputation processes. **A.** Gold standard WGS-based PRS percentile (x-axis) vs six replicates of imputation derived PRS percentiles (y-axis). Point darkness depicts point density for overplotting. **B.** Histogram of the absolute score deviations relative to the WGS-based standard. Note, bin for no change is not shown.

Fig S9. GPS$_{BC}$ Reproducibility.

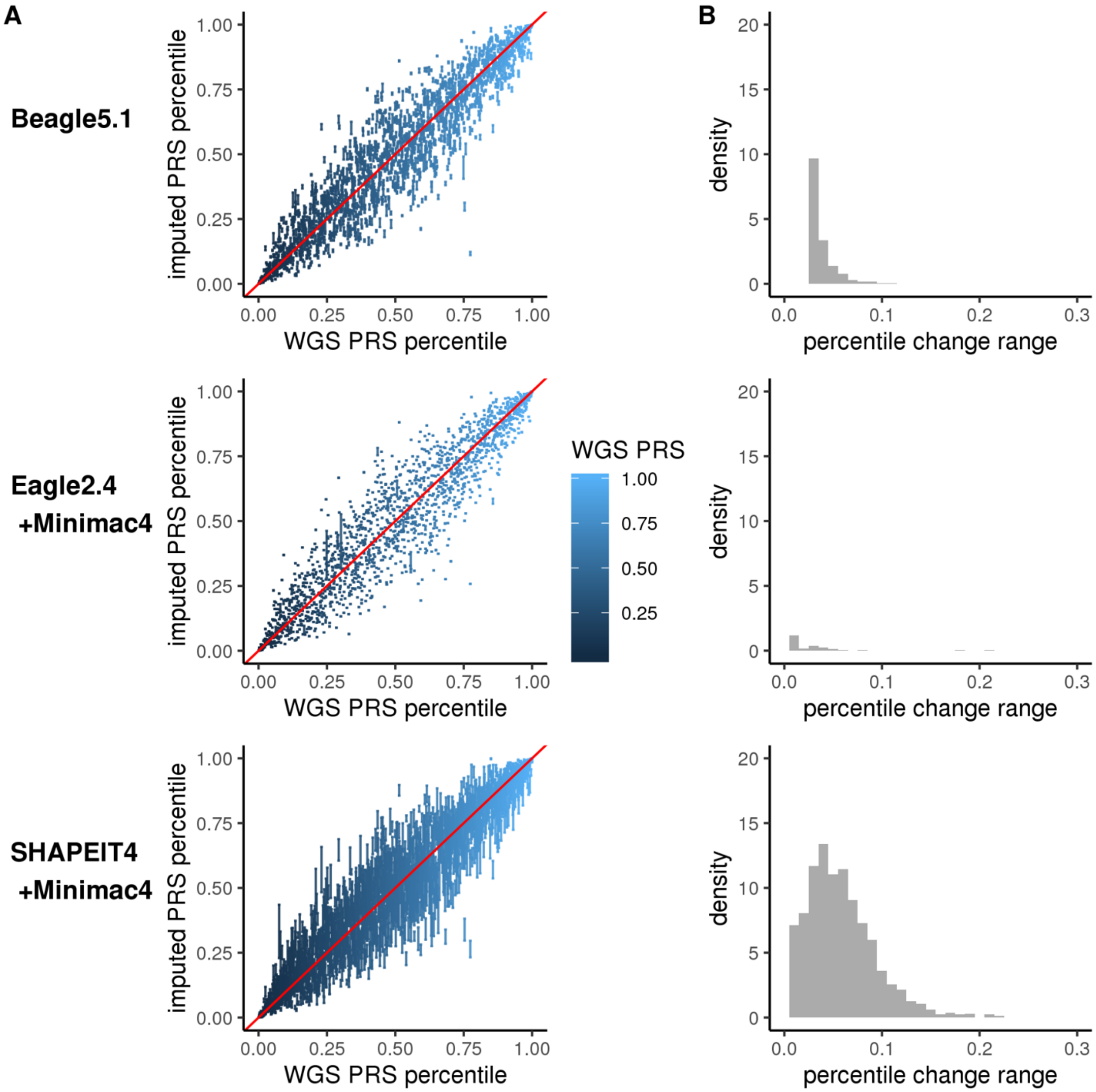

The variability in GPS$_{BC}$ percentile values as determined by three different imputation processes. **A.** Gold standard WGS-based PRS percentile (x-axis) vs six replicates of imputation derived PRS percentiles (y-axis). Point darkness depicts point density for overplotting. **B.** Histogram of the absolute score deviations relative to the WGS-based standard. Note, bin for no change is not shown.

Fig S10. PRS-GWAS$_{Afib}$ Reproducibility.

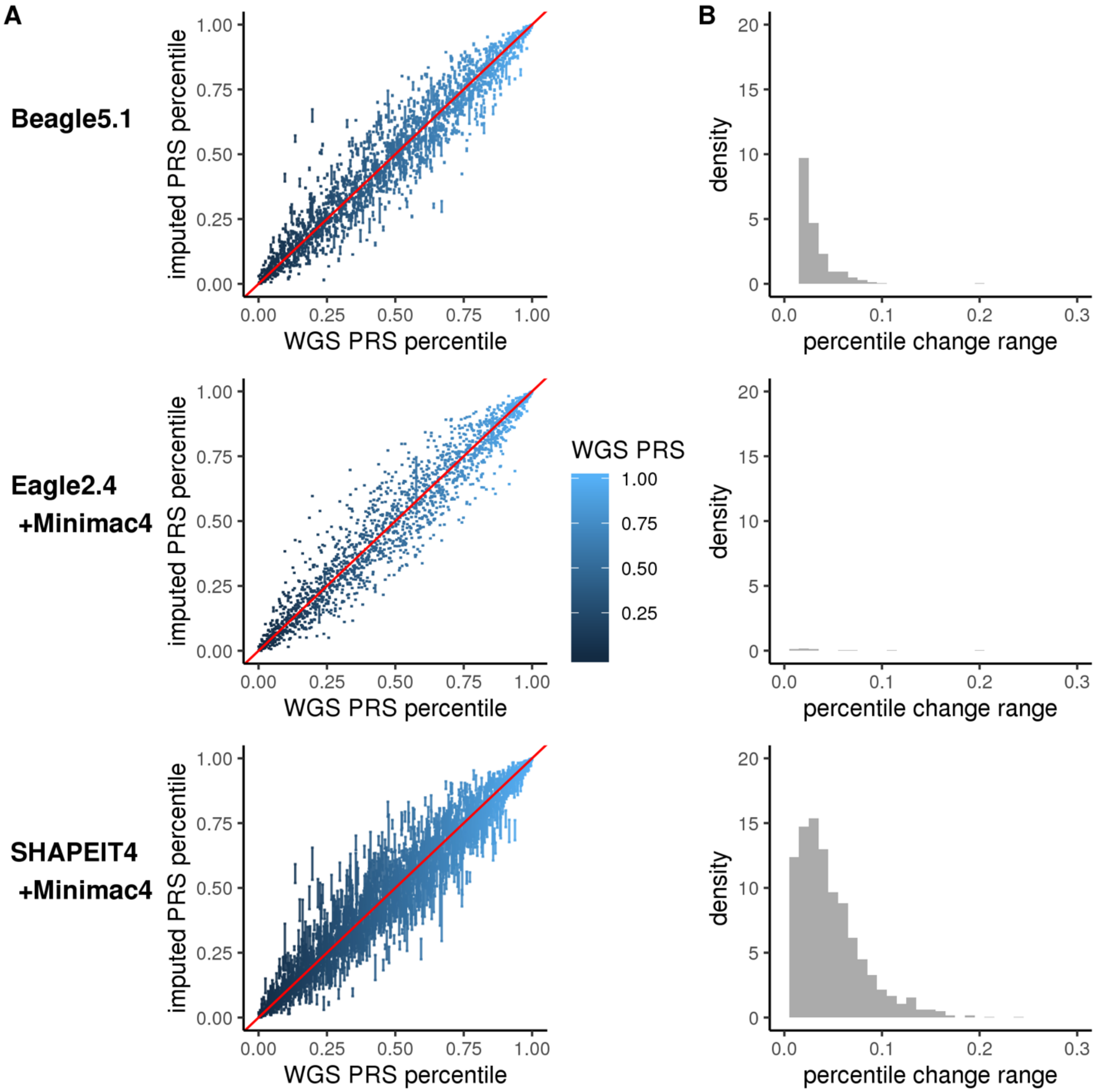

The variability in PRS-GWAS$_{Afib}$ percentile values as determined by three different imputation processes. **A.** Gold standard WGS-based PRS percentile (x-axis) vs six replicates of imputation derived PRS percentiles (y-axis). Point darkness depicts point density for overplotting. **B.** Histogram of the absolute score deviations relative to the WGS-based standard. Note, bin for no change is not shown.

Fig S11. GPS$_{Afib}$ Reproducibility.

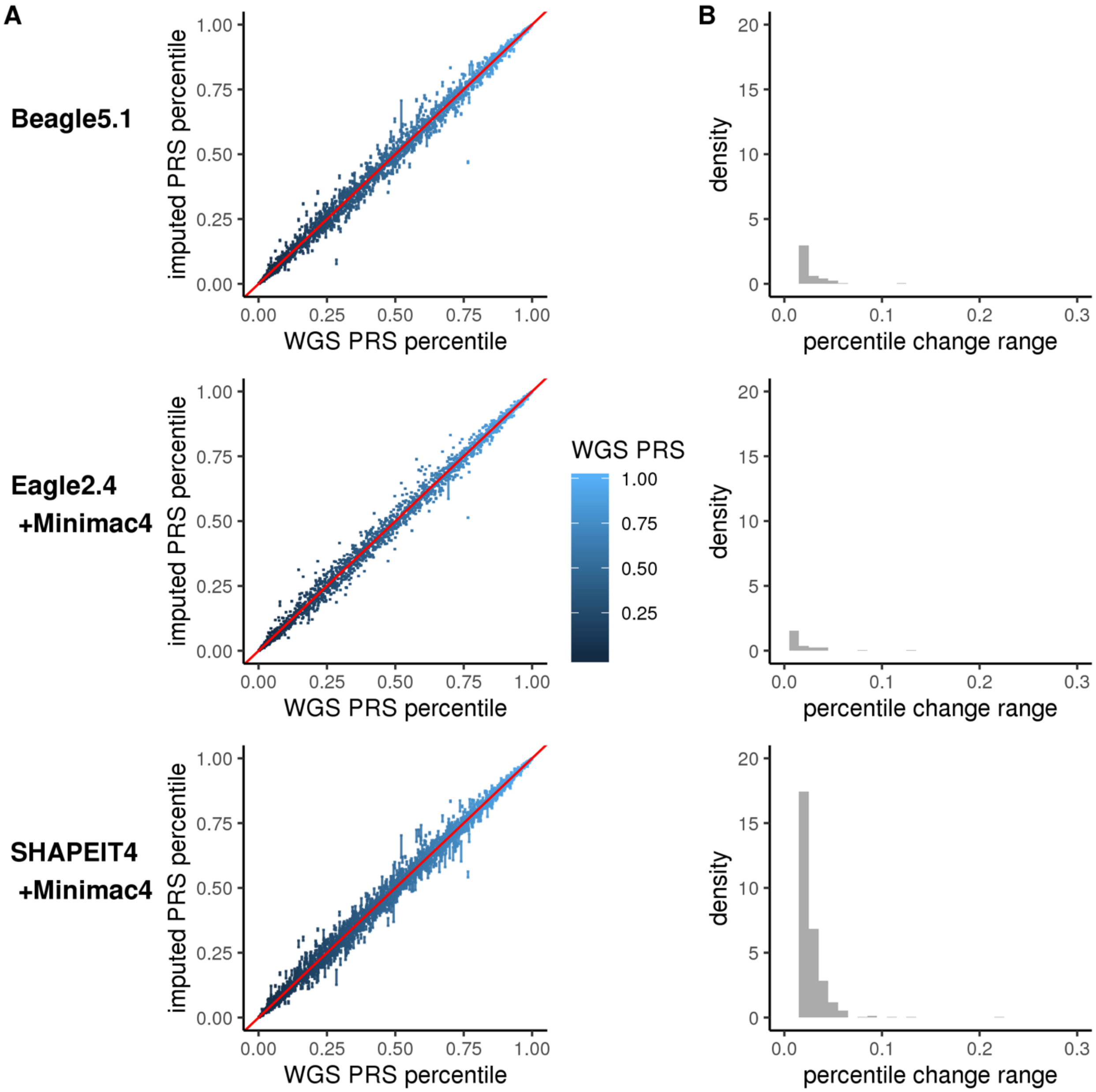

The variability in GPS$_{Afib}$ percentile values as determined by three different imputation processes. **A.** Gold standard WGS-based PRS percentile (x-axis) vs six replicates of imputation derived PRS percentiles (y-axis). Point darkness depicts point density for overplotting. **B.** Histogram of the absolute score deviations relative to the WGS-based standard. Note, bin for no change is not shown.

Fig S12. PRS-GWAS$_{AD}$ Reproducibility.

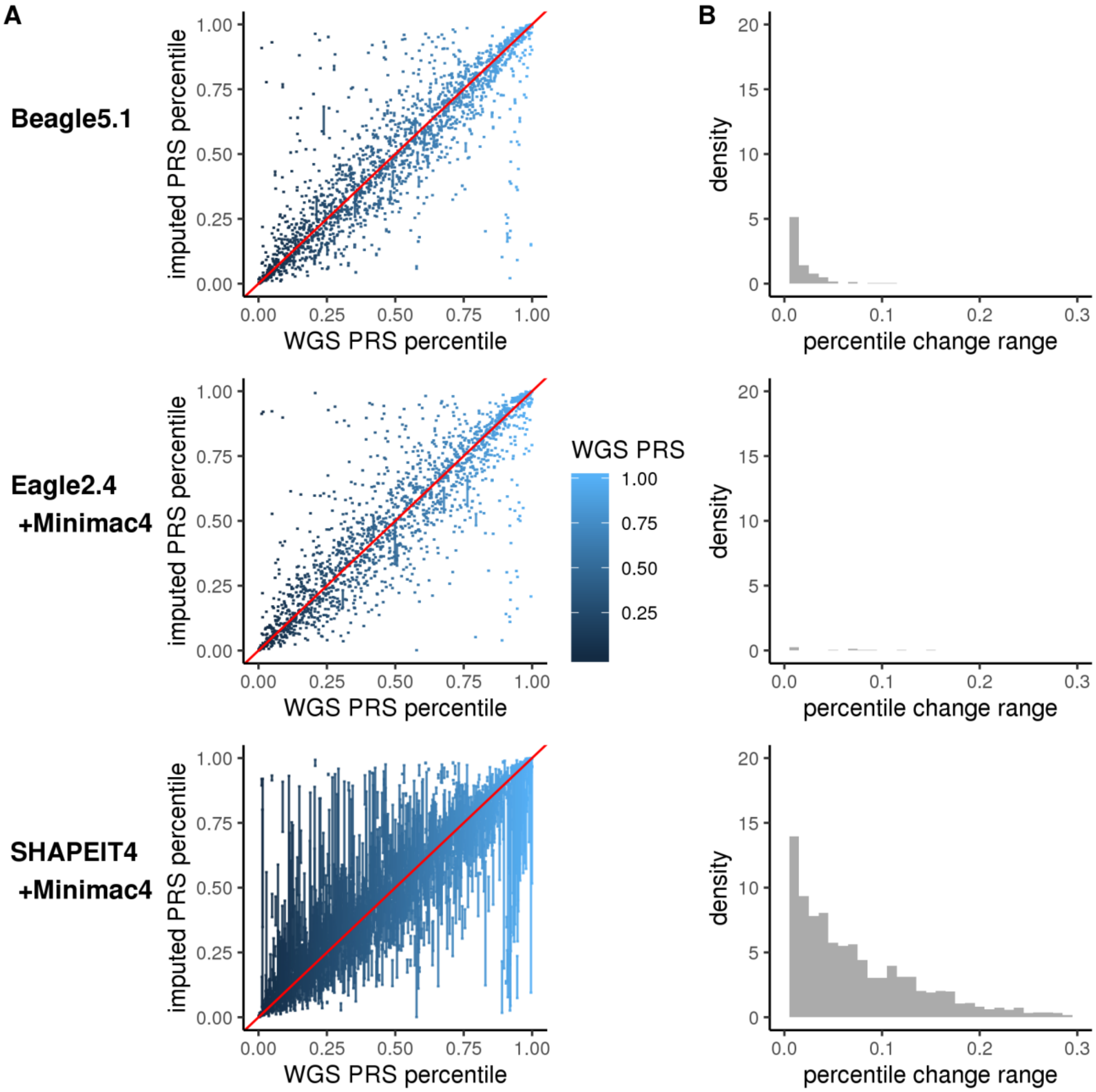

The variability in PRS-GWAS$_{AD}$ percentile values as determined by three different imputation processes. **A.** Gold standard WGS-based PRS percentile (x-axis) vs six replicates of imputation derived PRS percentiles (y-axis). Point darkness depicts point density for overplotting. **B.** Histogram of the absolute score deviations relative to the WGS-based standard. Note, bin for no change is not shown.

Fig S13. PRS-GWAS$_{Glaucoma}$ Reproducibility.

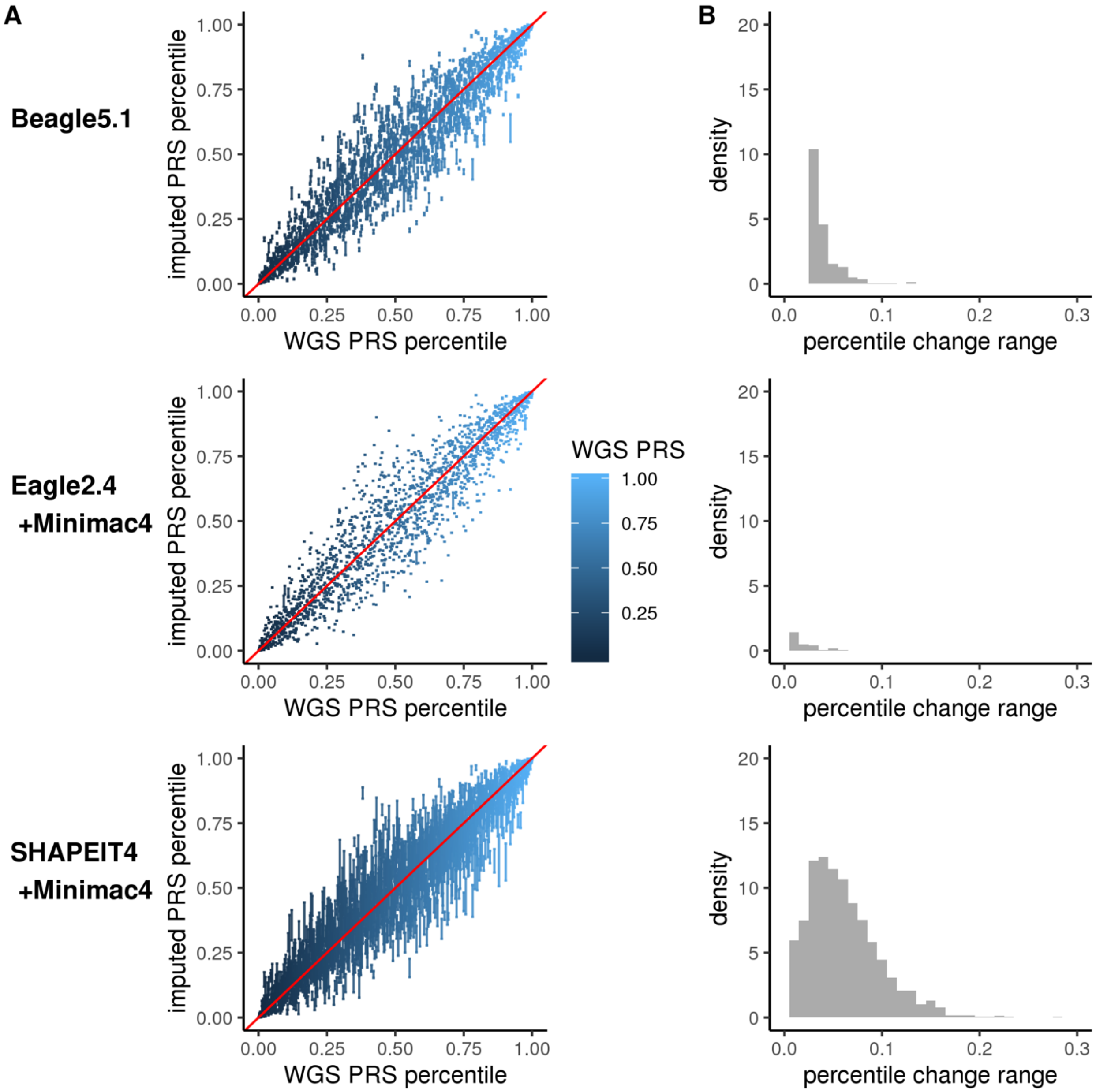

The variability in PRS-GWAS$_{Glaucoma}$ percentile values as determined by three different imputation processes. **A.** Gold standard WGS-based PRS percentile (x-axis) vs six replicates of imputation derived PRS percentiles (y-axis). Point darkness depicts point density for overplotting. **B.** Histogram of the absolute score deviations relative to the WGS-based standard. Note, bin for no change is not shown.

Fig S14. PRS_{CAD} Reproducibility by Ancestry.

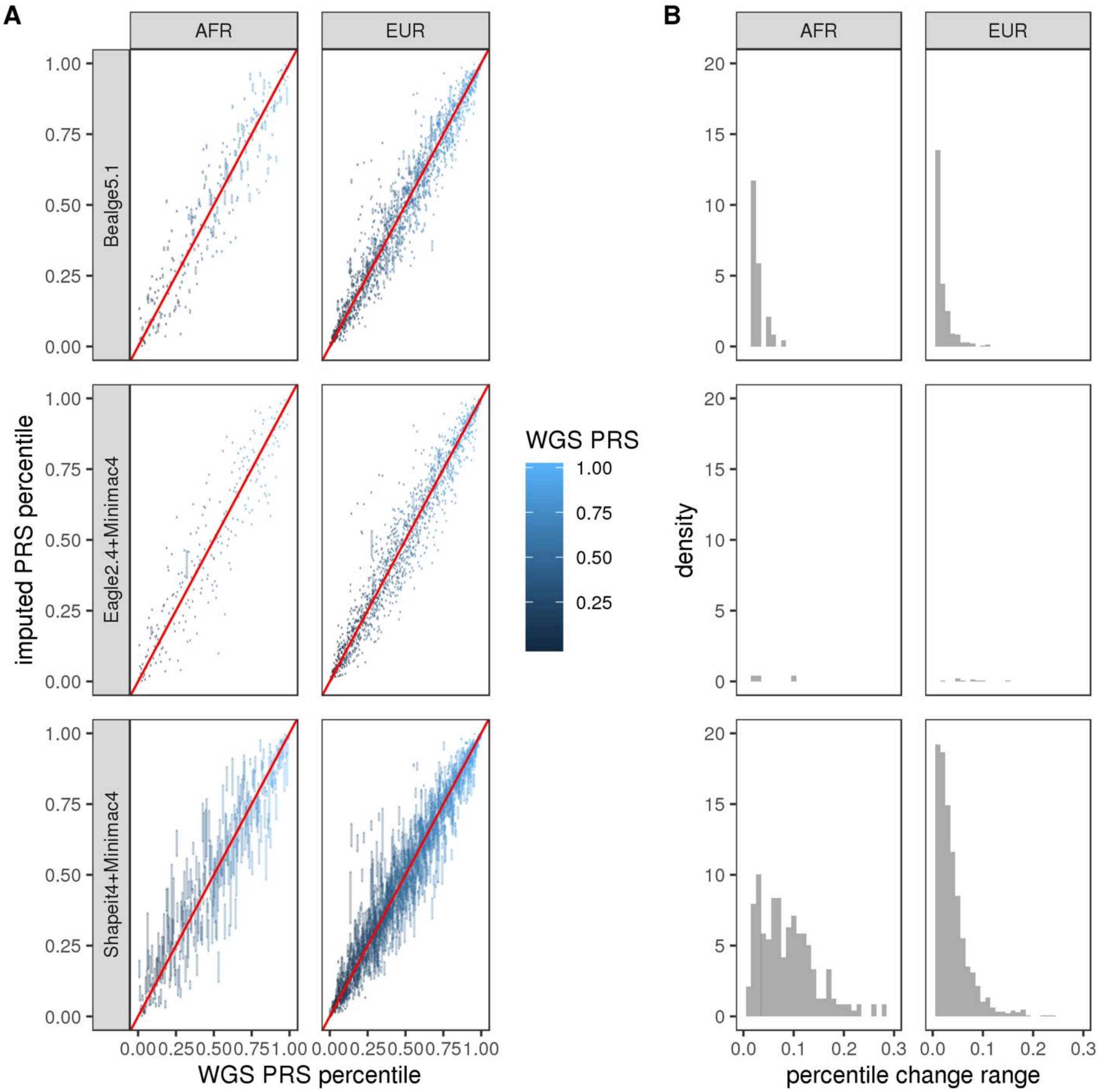

The variability in PRS_{CAD} percentile values as determined by three different imputation processes by 2 ancestries. **A.** Gold standard WGS-based PRS percentile (x-axis) vs six replicates of imputation derived PRS percentiles (y-axis). Point darkness depicts point density for overplotting. **B.** Histogram of the absolute score deviations relative to the WGS-based standard. Note, bin for no change is not shown. AFR: Afrian, EUR: European.

Fig S15. metaGRS$_{CAD}$ Reproducibility by Ancestry.

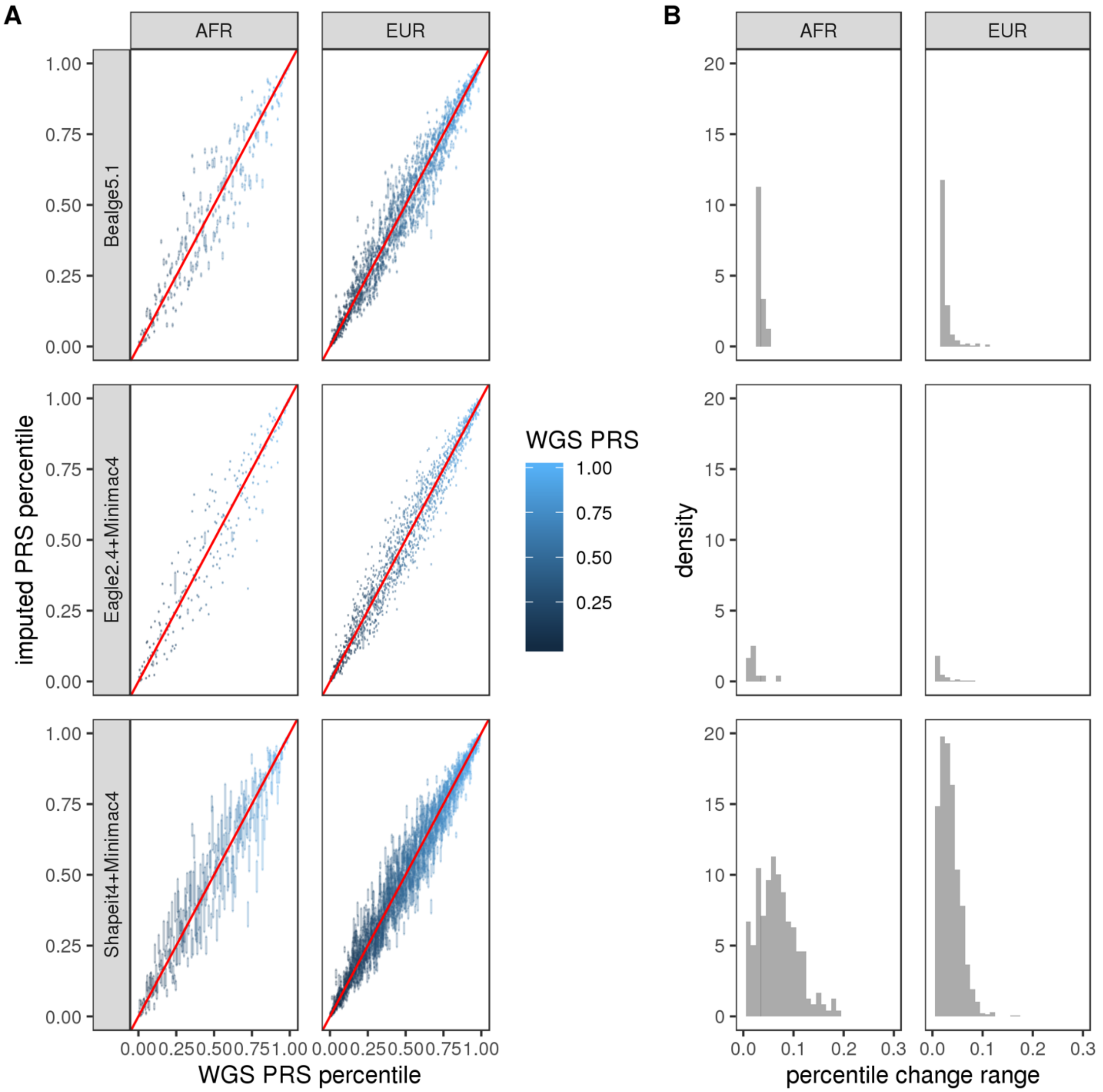

The variability in metaGRS$_{CAD}$ percentile values as determined by three different imputation processes by 2 ancestries. **A.** Gold standard WGS-based PRS percentile (x-axis) vs six replicates of imputation derived PRS percentiles (y-axis). Point darkness depicts point density for overplotting. **B.** Histogram of the absolute score deviations relative to the WGS-based standard. Note, bin for no change is not shown. AFR: Afrian, EUR: European.

Fig S16. GPS$_{CAD}$ Reproducibility by Ancestry.

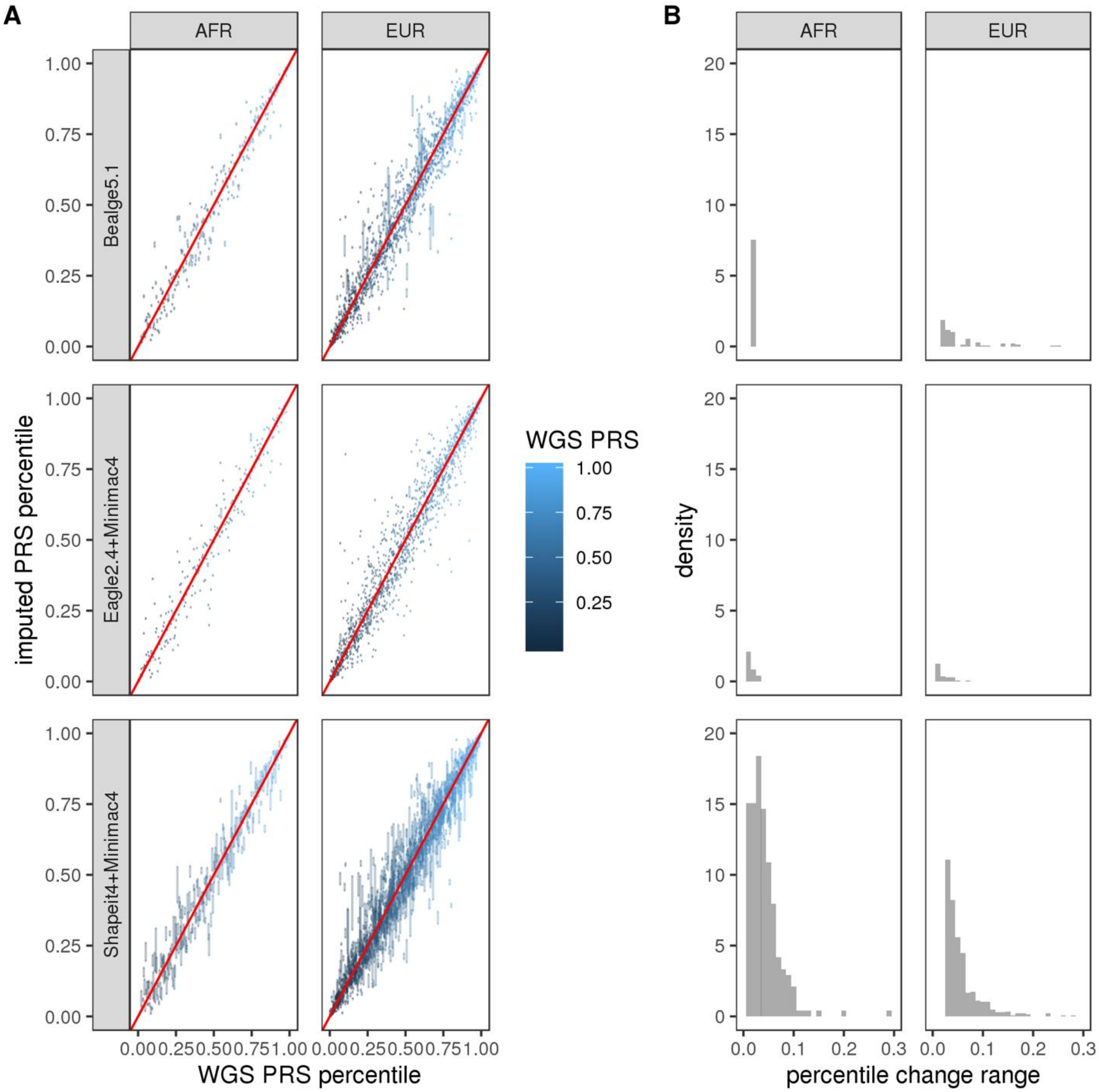

The variability in GPS$_{CAD}$ percentile values as determined by three different imputation processes by 2 ancestries. **A.** Gold standard WGS-based PRS percentile (x-axis) vs six replicates of imputation derived PRS percentiles (y-axis). Point darkness depicts point density for overplotting. **B.** Histogram of the absolute score deviations relative to the WGS-based standard. Note, bin for no change is not shown. AFR: Afrian, EUR: European.

Fig S17. PRS-GWAS$_{T2D}$ (547) Reproducibility by Ancestry.

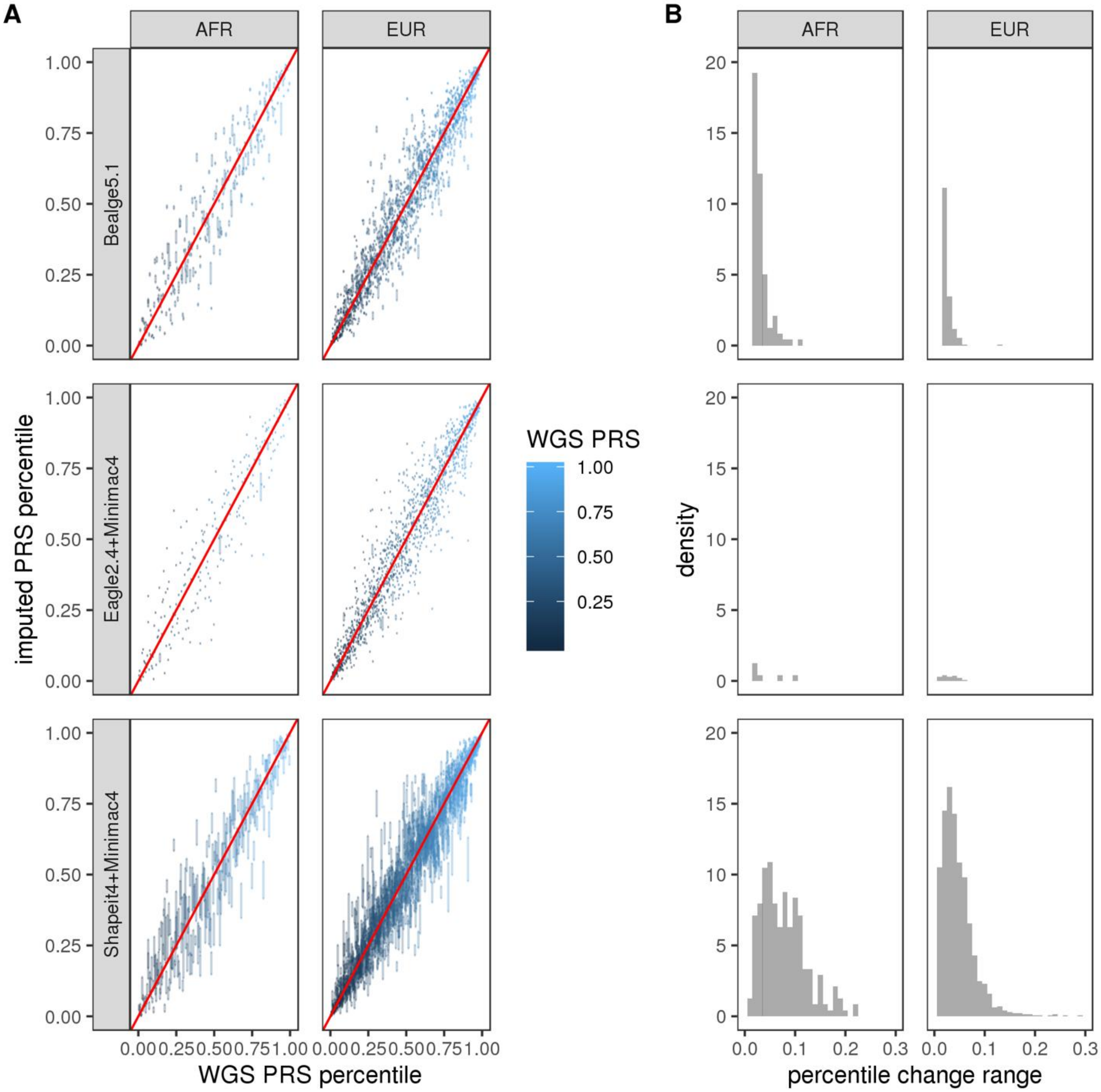

The variability in PRS-GWAS$_{T2D}$ (547) percentile values as determined by three different imputation processes by 2 ancestries. **A.** Gold standard WGS-based PRS percentile (x-axis) vs six replicates of imputation derived PRS percentiles (y-axis). Point darkness depicts point density for overplotting. **B.** Histogram of the absolute score deviations relative to the WGS-based standard. Note, bin for no change is not shown. AFR: Afrian, EUR: European.

Fig S18. PRS-GWAS$_{T2D}$ (397) Reproducibility by Ancestry.

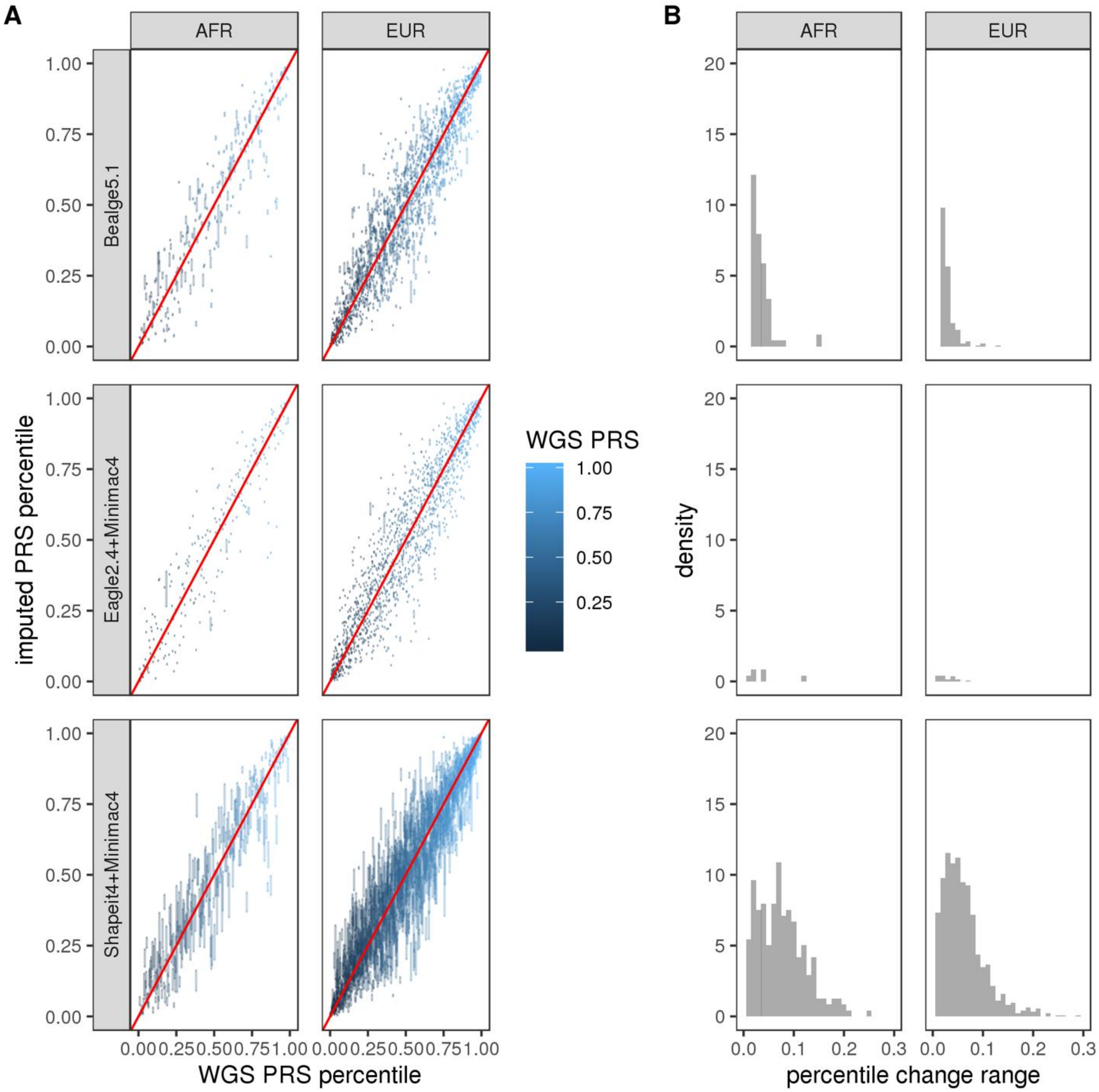

The variability in PRS-GWAS$_{T2D}$ (397) percentile values as determined by three different imputation processes by 2 ancestries. **A.** Gold standard WGS-based PRS percentile (x-axis) vs six replicates of imputation derived PRS percentiles (y-axis). Point darkness depicts point density for overplotting. **B.** Histogram of the absolute score deviations relative to the WGS-based standard. Note, bin for no change is not shown. AFR: Afrian, EUR: European.

Fig S19. PRS-GWAS$_{T2D}$ (170487) Reproducibility by Ancestry.

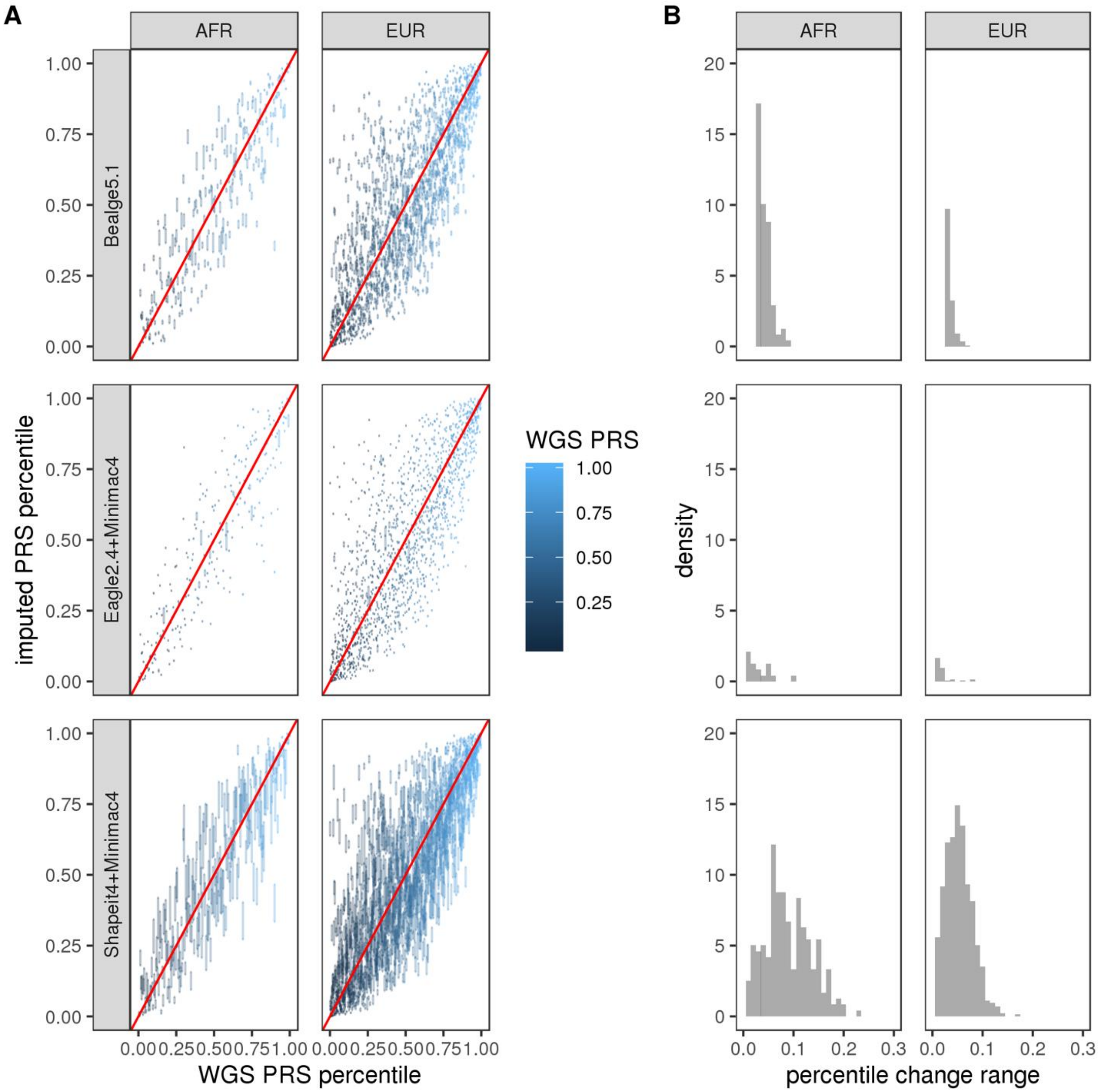

The variability in PRS-GWAS$_{T2D}$ (170487) percentile values as determined by three different imputation processes by 2 ancestries. **A.** Gold standard WGS-based PRS percentile (x-axis) vs six replicates of imputation derived PRS percentiles (y-axis). Point darkness depicts point density for overplotting. **B.** Histogram of the absolute score deviations relative to the WGS-based standard. Note, bin for no change is not shown. AFR: Afrian, EUR: European.

Fig S20. GPS_{T2D} Reproducibility by Ancestry.

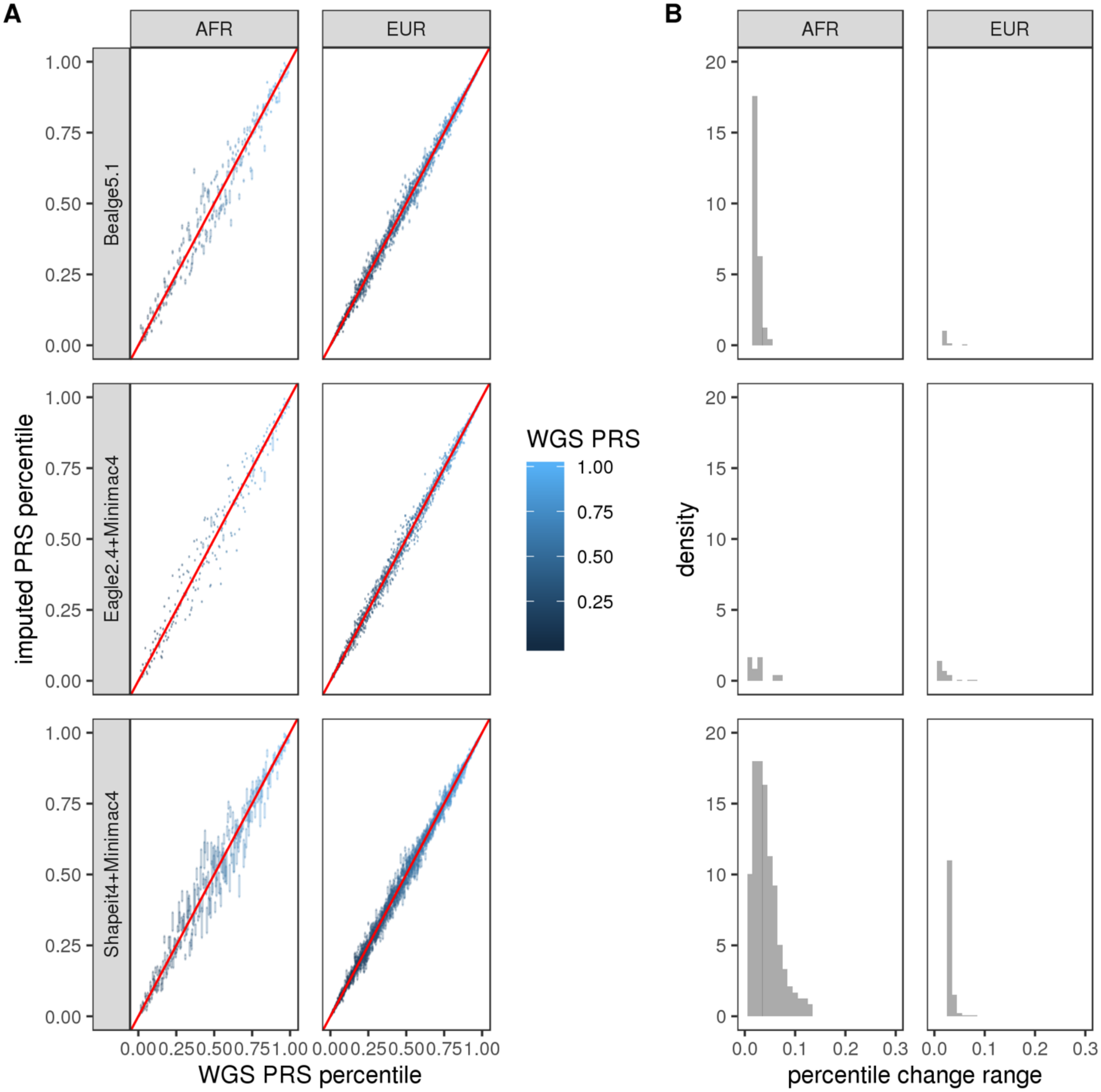

The variability in GPS_{T2D} percentile values as determined by three different imputation processes by 2 ancestries. **A.** Gold standard WGS-based PRS percentile (x-axis) vs six replicates of imputation derived PRS percentiles (y-axis). Point darkness depicts point density for overplotting. **B.** Histogram of the absolute score deviations relative to the WGS-based standard. Note, bin for no change is not shown. AFR: Afrian, EUR: European.

Fig S21. PRS-GWAS$_{BC}$ (239) Reproducibility by Ancestry.

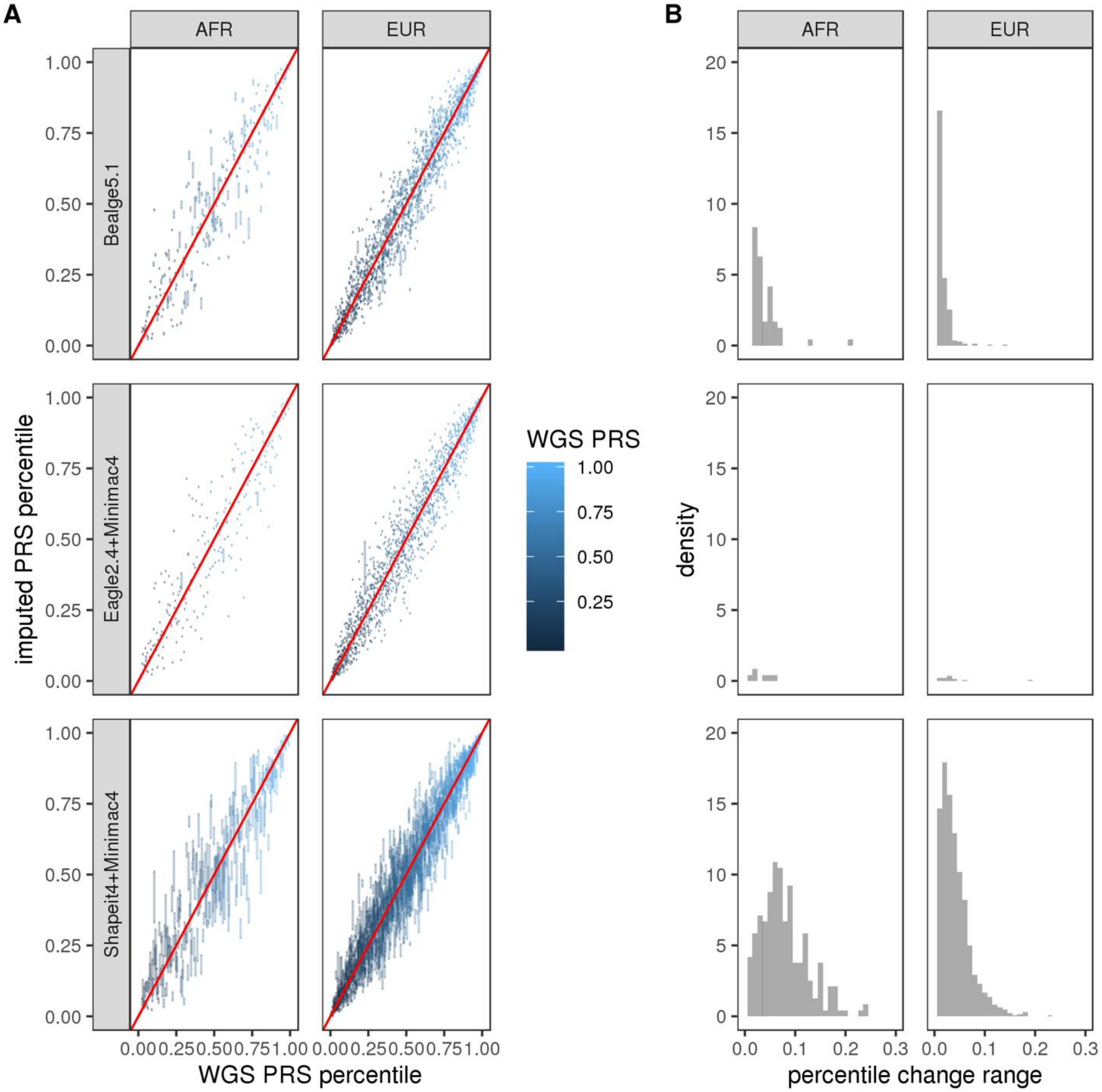

The variability in PRS-GWAS$_{BC}$ (239) percentile values as determined by three different imputation processes by 2 ancestries. **A.** Gold standard WGS-based PRS percentile (x-axis) vs six replicates of imputation derived PRS percentiles (y-axis). Point darkness depicts point density for overplotting. **B.** Histogram of the absolute score deviations relative to the WGS-based standard. Note, bin for no change is not shown. AFR: Afrian, EUR: European.

Fig S22. PRS-GWAS$_{BC}$ (2935) Reproducibility by Ancestry.

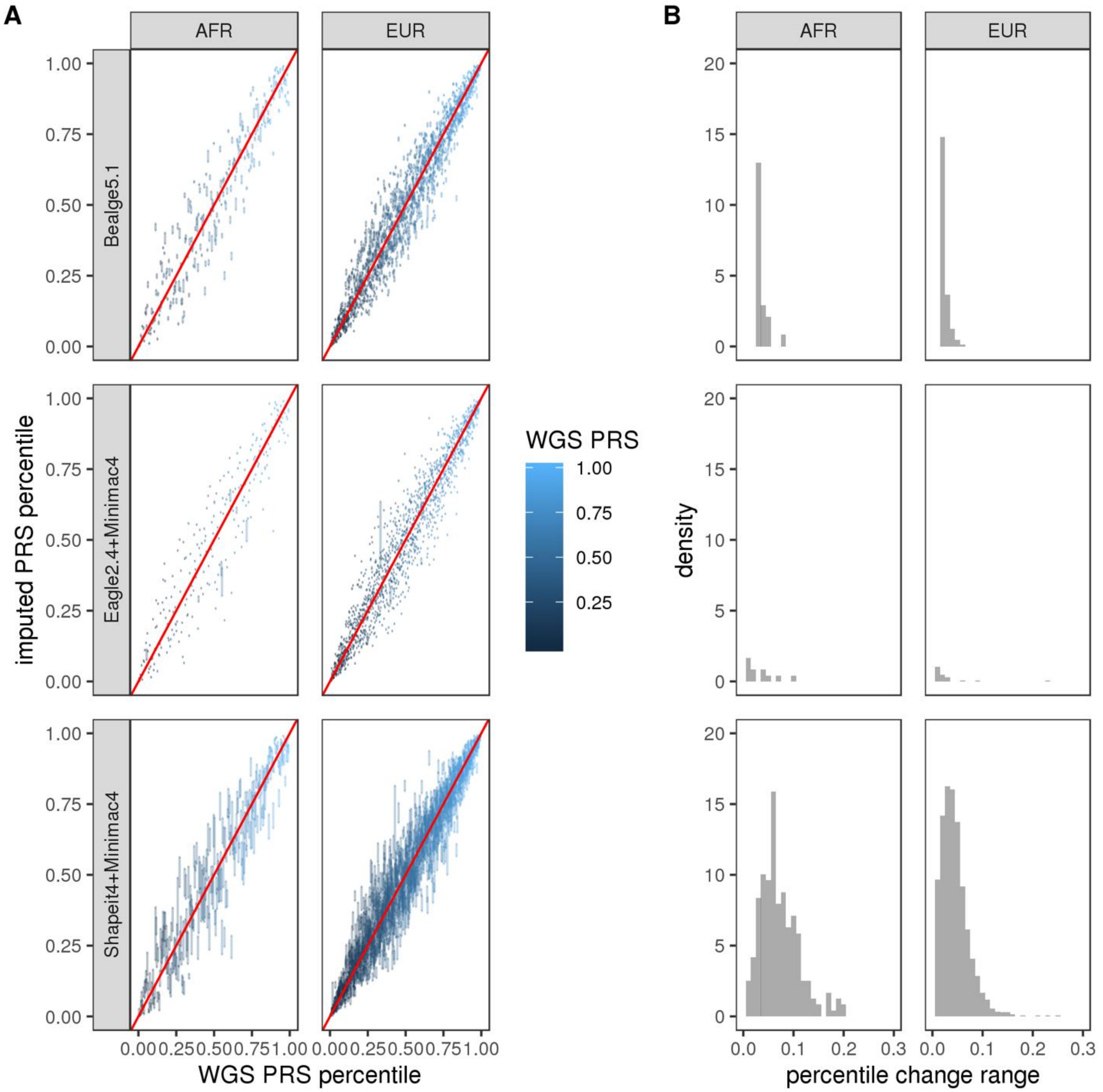

The variability in PRS-GWAS$_{BC}$ (2935) percentile values as determined by three different imputation processes by 2 ancestries. **A.** Gold standard WGS-based PRS percentile (x-axis) vs six replicates of imputation derived PRS percentiles (y-axis). Point darkness depicts point density for overplotting. **B.** Histogram of the absolute score deviations relative to the WGS-based standard. Note, bin for no change is not shown. AFR: Afrian, EUR: European.

Fig S23. GPS$_{BC}$ Reproducibility by Ancestry.

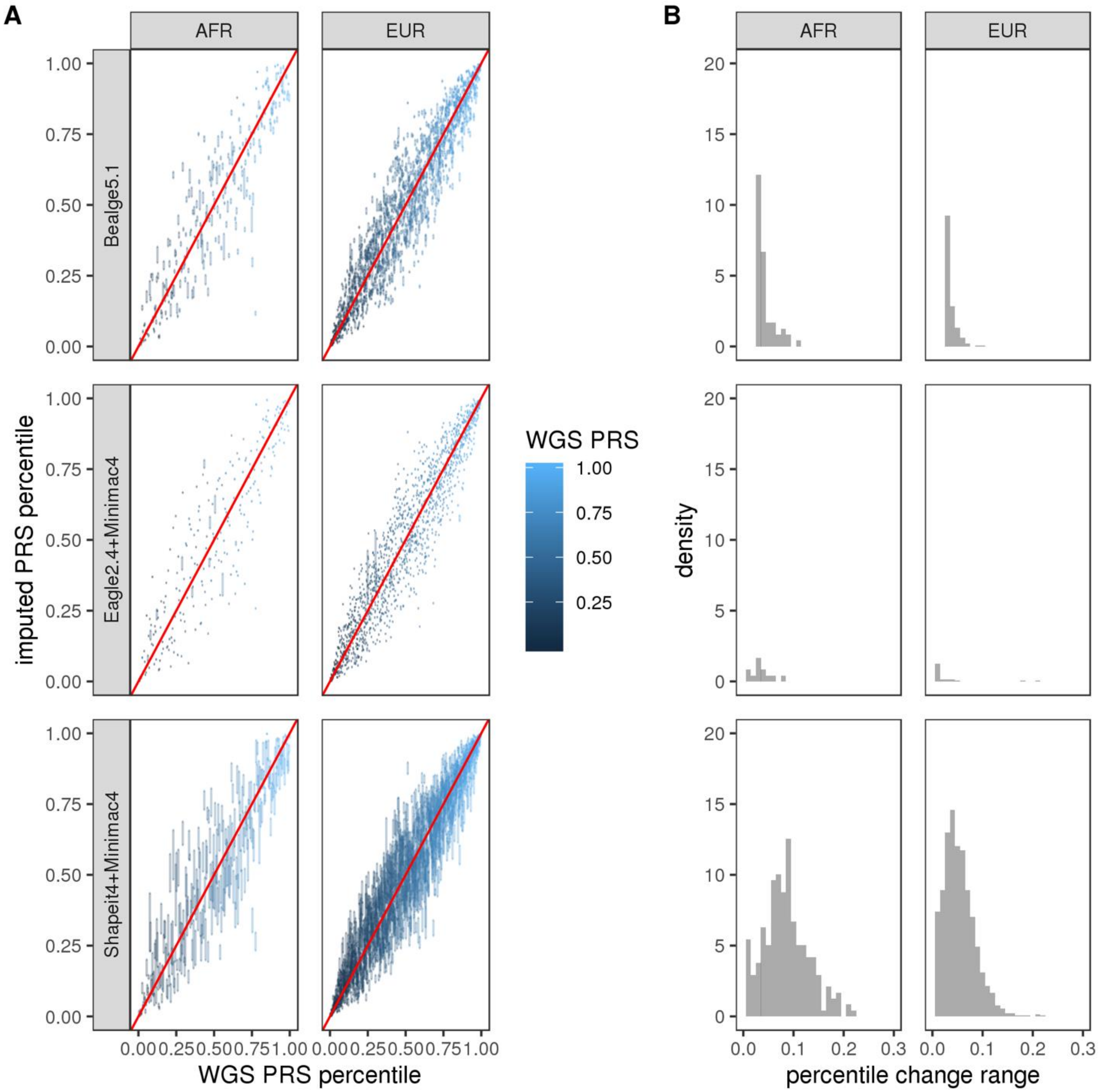

The variability in GPS$_{BC}$ percentile values as determined by three different imputation processes by 2 ancestries. **A.** Gold standard WGS-based PRS percentile (x-axis) vs six replicates of imputation derived PRS percentiles (y-axis). Point darkness depicts point density for overplotting. **B.** Histogram of the absolute score deviations relative to the WGS-based standard. Note, bin for no change is not shown. AFR: Afrian, EUR: European.

Fig S24. PRS-GWAS$_{Afib}$ Reproducibility by Ancestry.

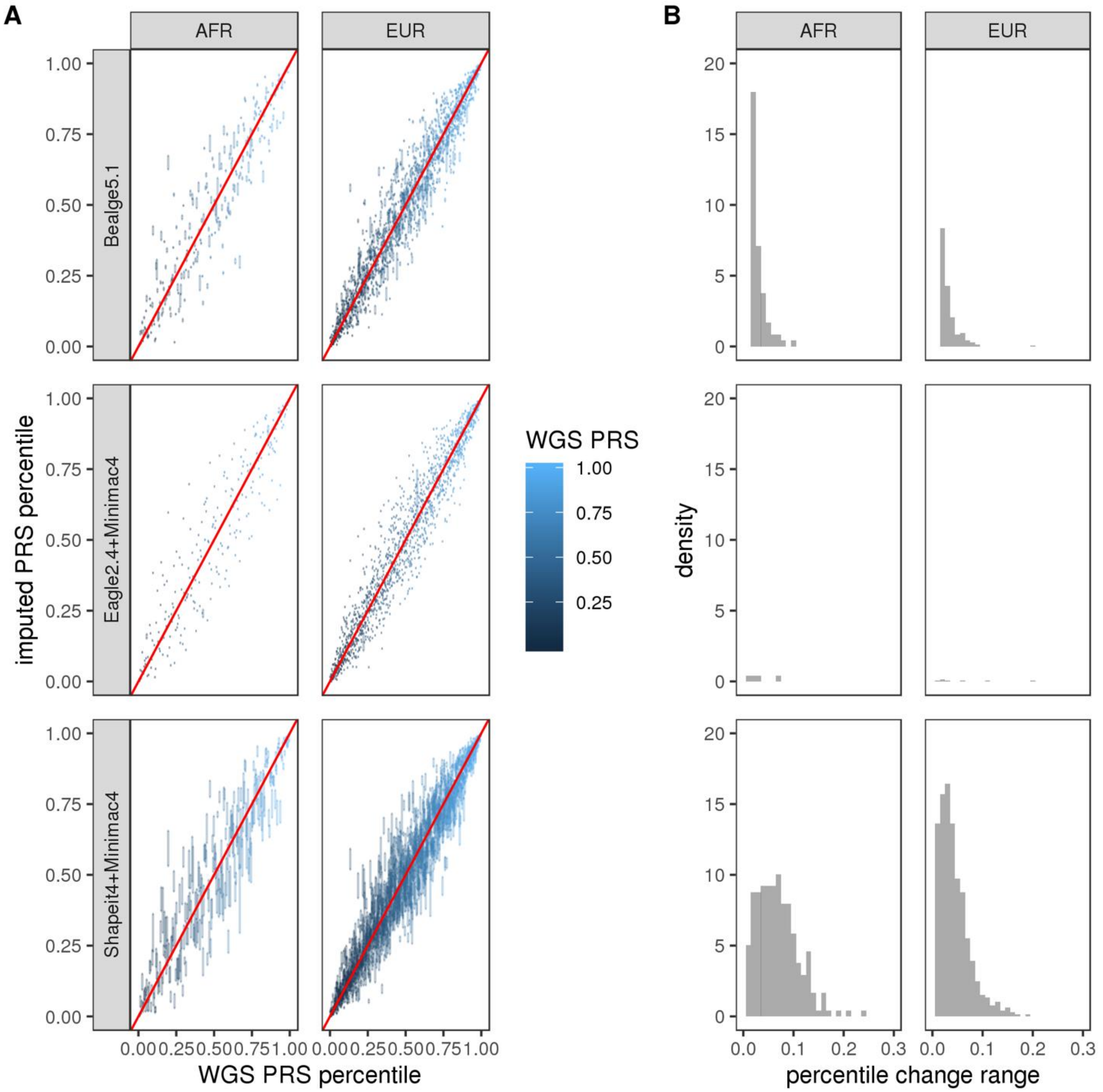

The variability in PRS-GWAS$_{Afib}$ percentile values as determined by three different imputation processes by 2 ancestries. **A.** Gold standard WGS-based PRS percentile (x-axis) vs six replicates of imputation derived PRS percentiles (y-axis). Point darkness depicts point density for overplotting. **B.** Histogram of the absolute score deviations relative to the WGS-based standard. Note, bin for no change is not shown. AFR: Afrian, EUR: European.

Fig S25. GPS_{Afib} Reproducibility by Ancestry.

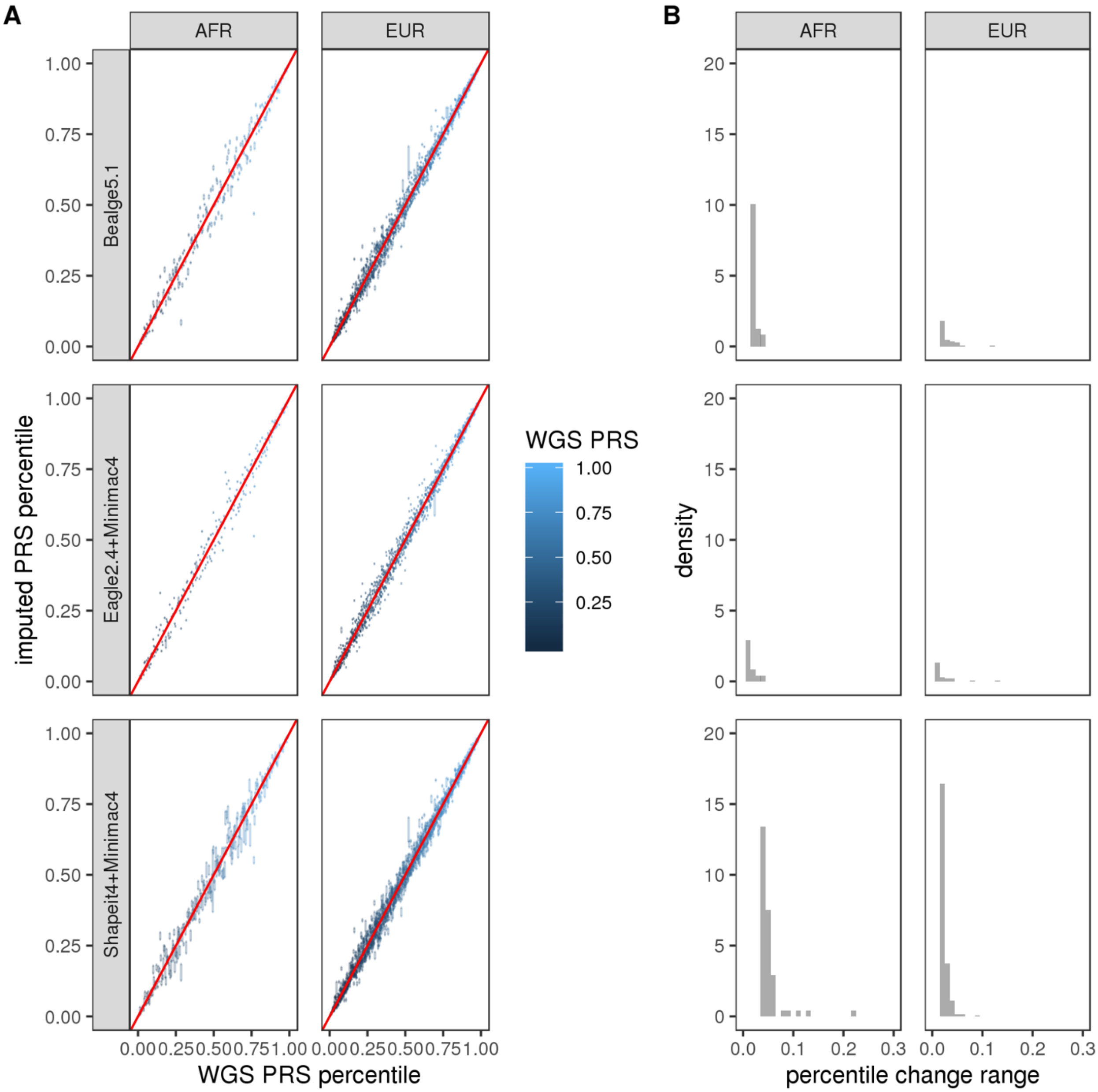

The variability in GPS_{Afib} percentile values as determined by three different imputation processes by 2 ancestries. **A.** Gold standard WGS-based PRS percentile (x-axis) vs six replicates of imputation derived PRS percentiles (y-axis). Point darkness depicts point density for overplotting. **B.** Histogram of the absolute score deviations relative to the WGS-based standard. Note, bin for no change is not shown. AFR: Afrian, EUR: European.

Fig S26. PRS-GWAS$_{AD}$ Reproducibility by Ancestry.

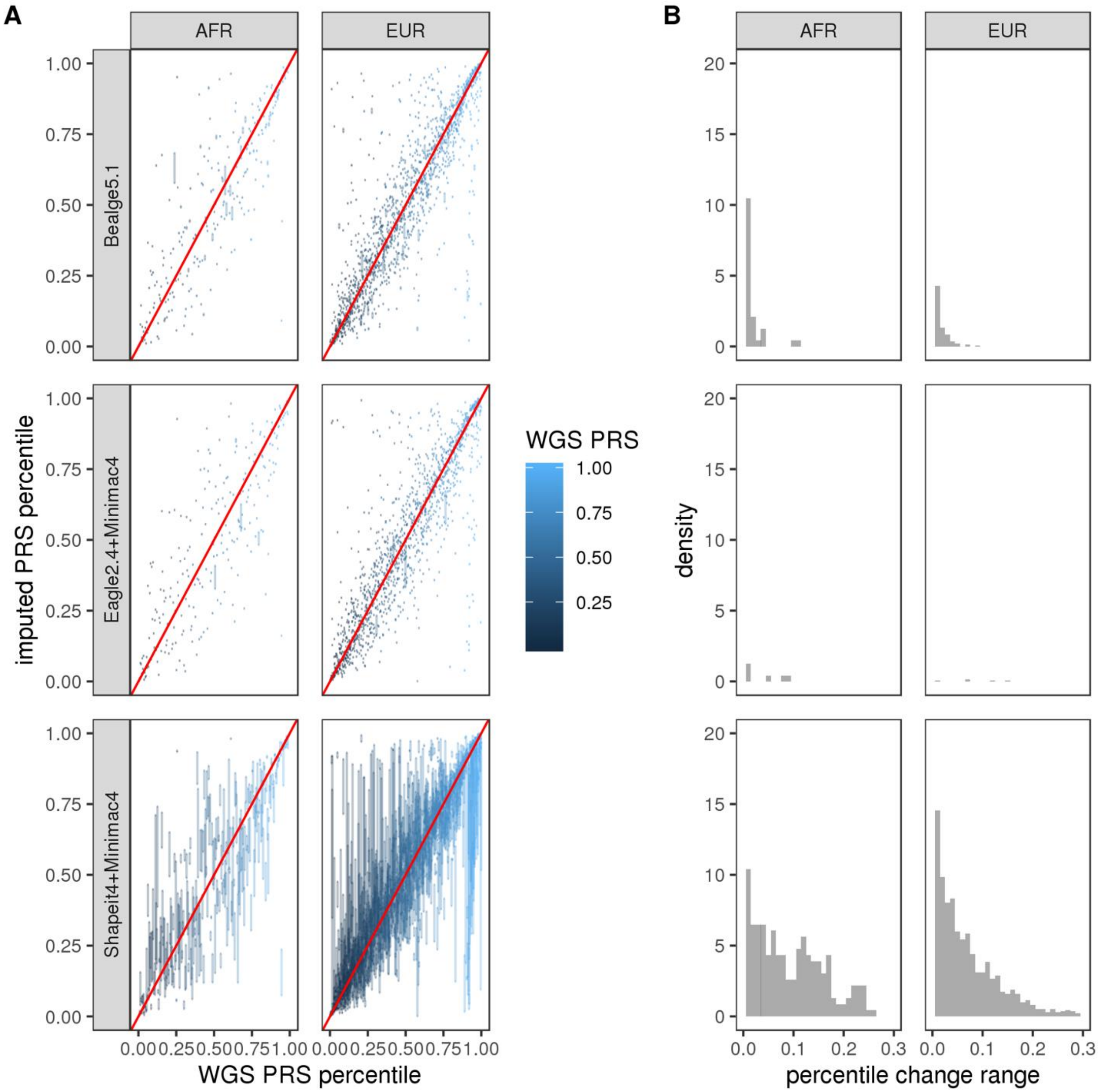

The variability in PRS-GWAS$_{AD}$ percentile values as determined by three different imputation processes by 2 ancestries. **A.** Gold standard WGS-based PRS percentile (x-axis) vs six replicates of imputation derived PRS percentiles (y-axis). Point darkness depicts point density for overplotting. **B.** Histogram of the absolute score deviations relative to the WGS-based standard. Note, bin for no change is not shown. AFR: Afrian, EUR: European.

Fig S27. PRS-GWAS$_{Glaucoma}$ Reproducibility by Ancestry.

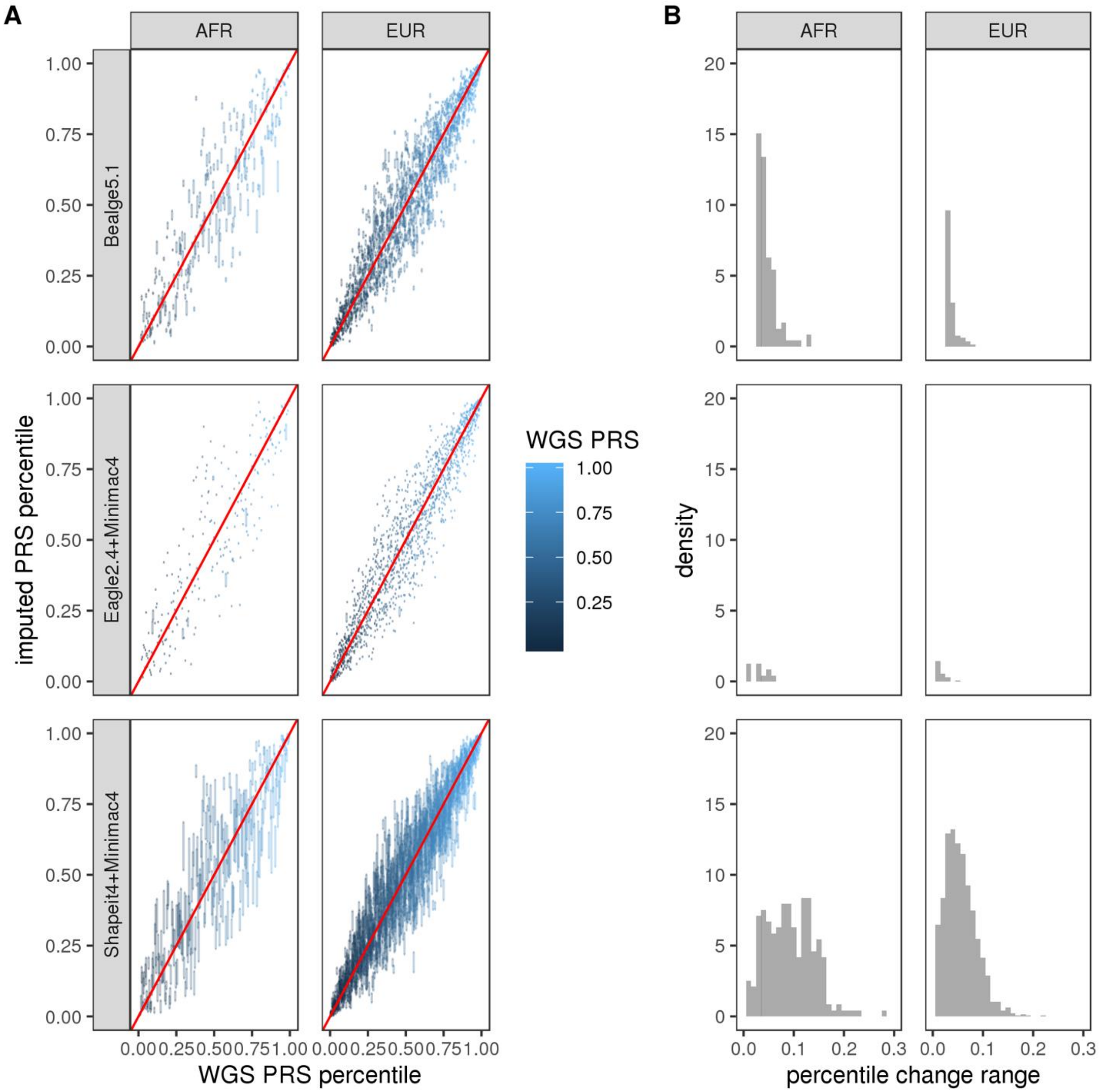

The variability in PRS-GWAS$_{Glaucoma}$ percentile values as determined by three different imputation processes by 2 ancestries. **A.** Gold standard WGS-based PRS percentile (x-axis) vs six replicates of imputation derived PRS percentiles (y-axis). Point darkness depicts point density for overplotting. **B.** Histogram of the absolute score deviations relative to the WGS-based standard. Note, bin for no change is not shown. AFR: Afrian, EUR: European.

Fig S28. metaGRS$_{CAD}$ Variability as a Function of PRS Bin.

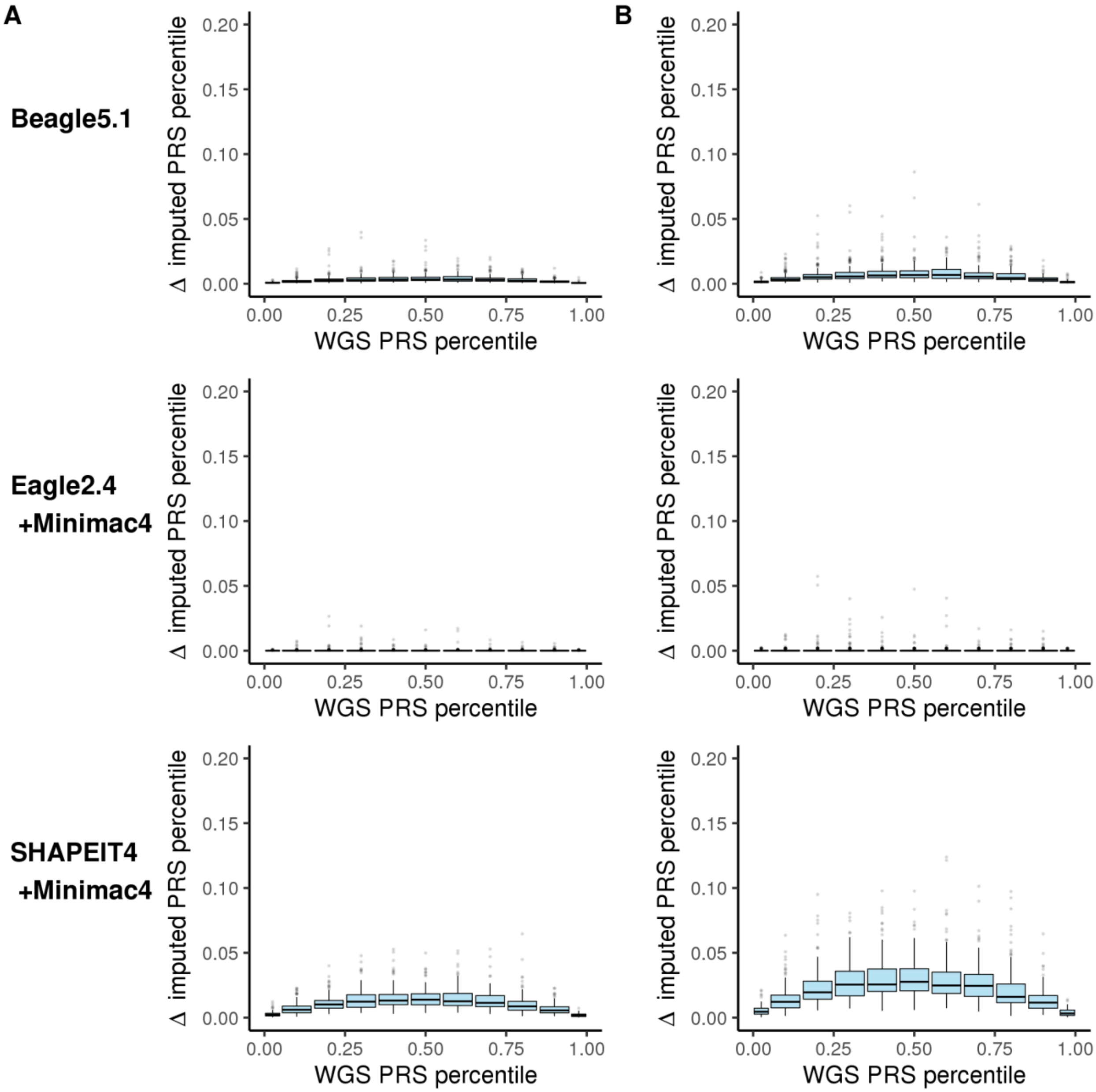

The degree of variability in PRS percentile as a function of the expected WGS-based PRS tier across three different imputation processes. **A.** Average absolute deviation per individual relative to their WGS-based gold standard. **B.** Maximum absolute deviation per individual relative to the their WGS-based gold standard. Box plots depict the interquartile range as is standard.

Fig S29. GPS_{CAD} Variability as a Function of PRS Bin.

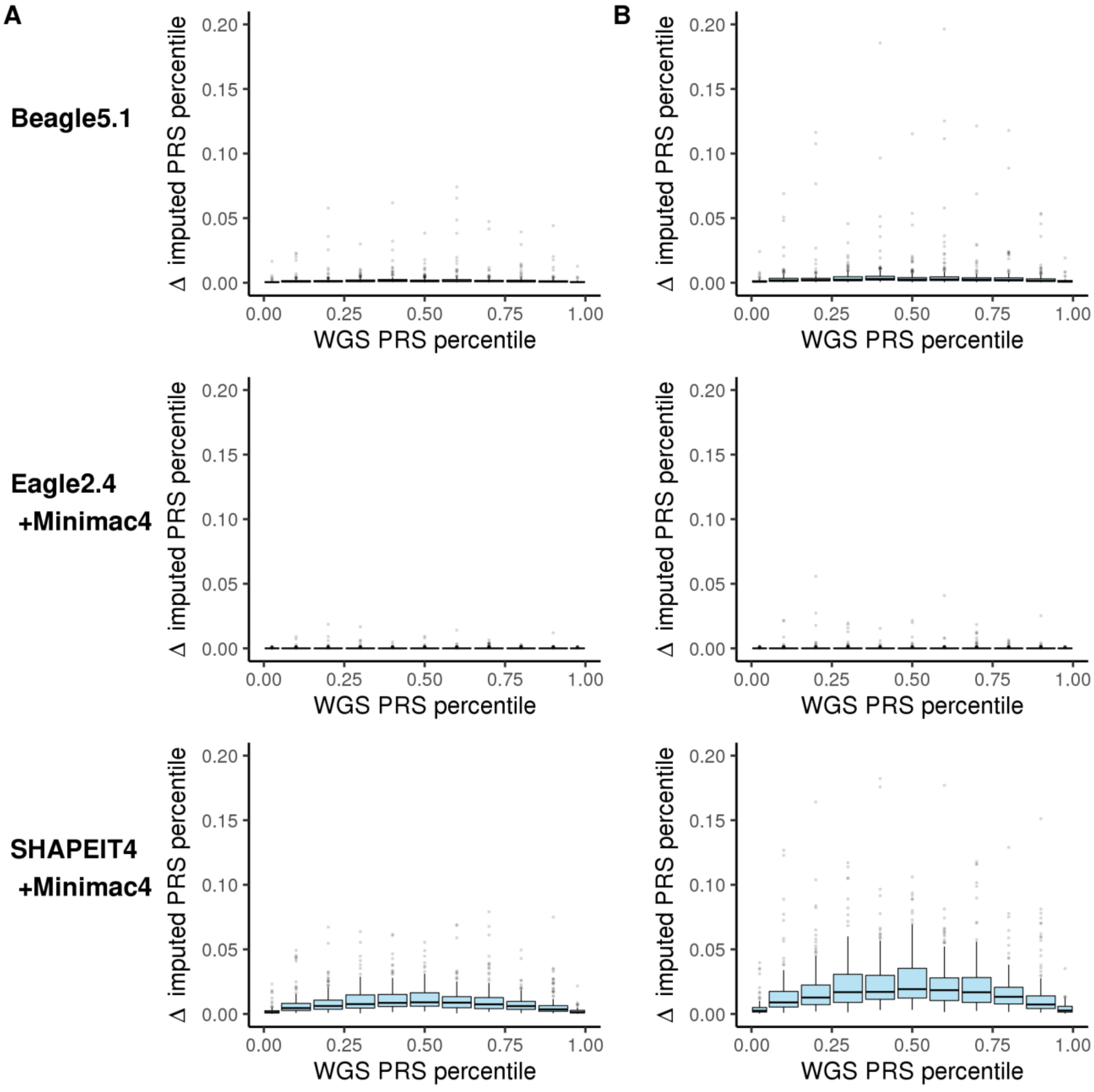

The degree of variability in PRS percentile as a function of the expected WGS-based PRS tier across three different imputation processes. **A.** Average absolute deviation per individual relative to their WGS-based gold standard. **B.** Maximum absolute deviation per individual relative to the their WGS-based gold standard. Box plots depict the interquartile range as is standard.

Fig S30. PRS-GWAS$_{T2D}$ (547) Variability as a Function of PRS Bin.

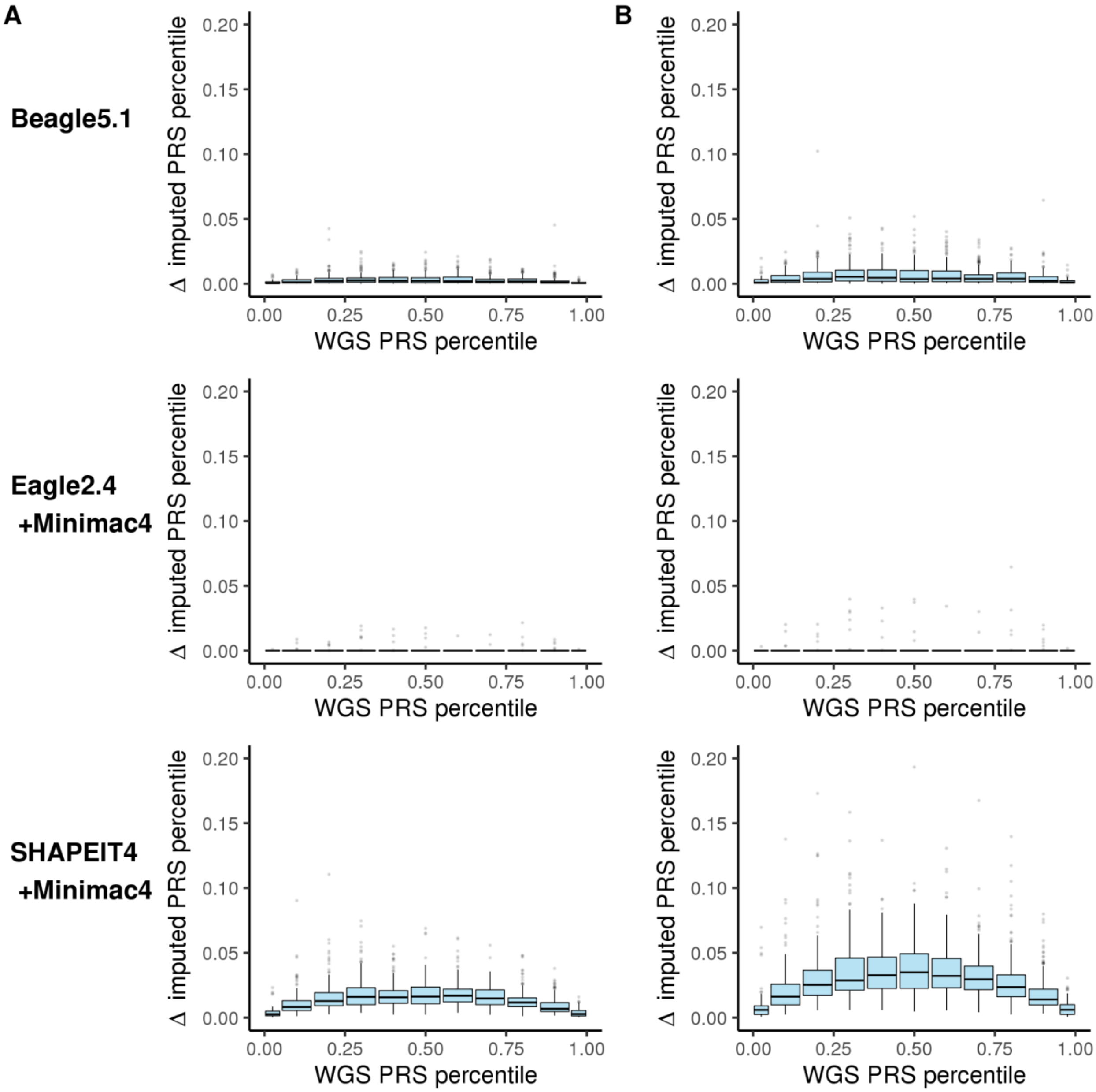

The degree of variability in PRS percentile as a function of the expected WGS-based PRS tier across three different imputation processes. **A.** Average absolute deviation per individual relative to their WGS-based gold standard. **B.** Maximum absolute deviation per individual relative to the their WGS-based gold standard. Box plots depict the interquartile range as is standard.

Fig S31. PRS-GWAS$_{T2D}$ (397) Variability as a Function of PRS Bin.

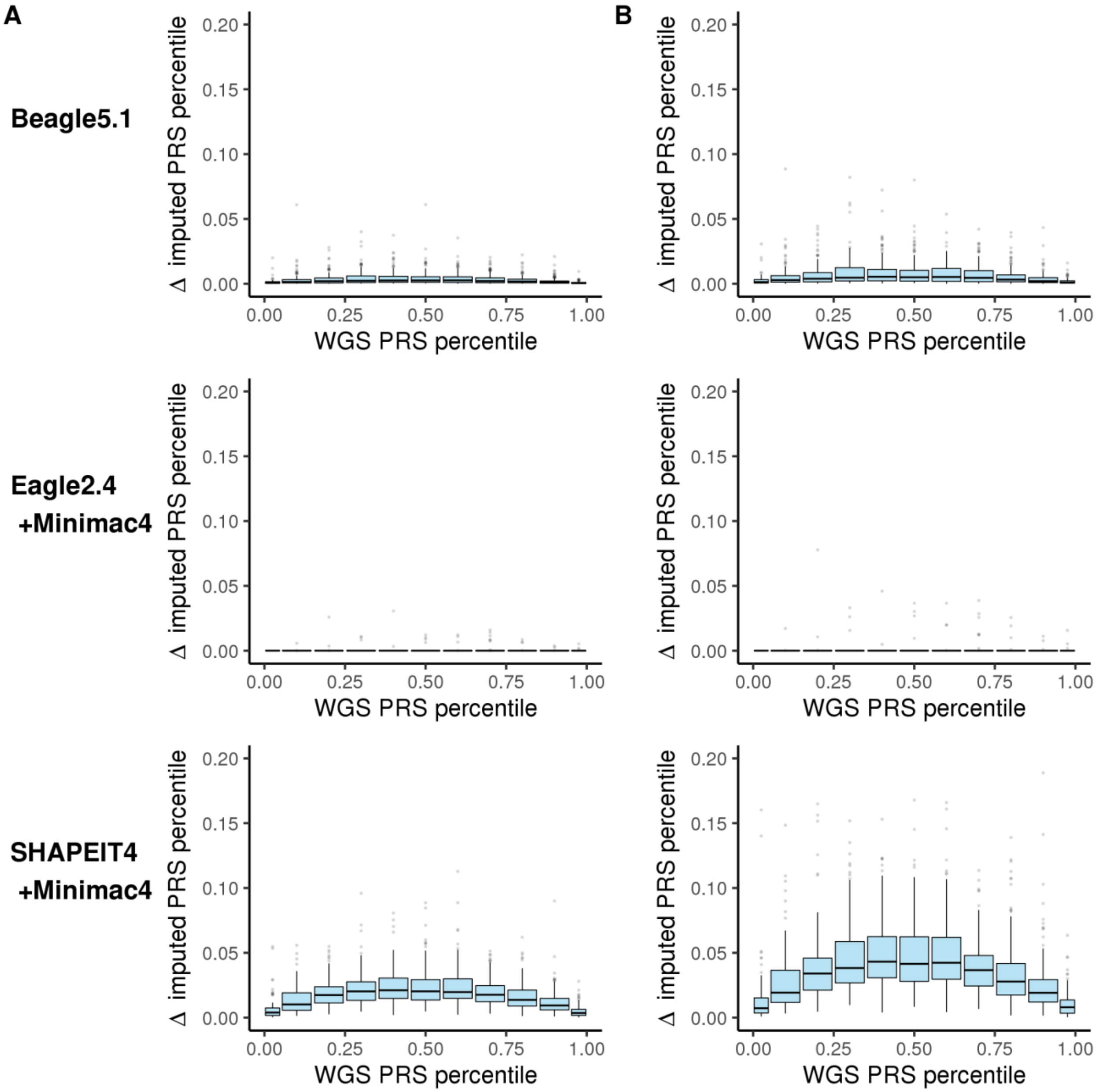

The degree of variability in PRS percentile as a function of the expected WGS-based PRS tier across three different imputation processes. **A.** Average absolute deviation per individual relative to their WGS-based gold standard. **B.** Maximum absolute deviation per individual relative to the their WGS-based gold standard. Box plots depict the interquartile range as is standard.

Fig S32. PRS-GWAS$_{T2D}$ (170487) Variability as a Function of PRS Bin.

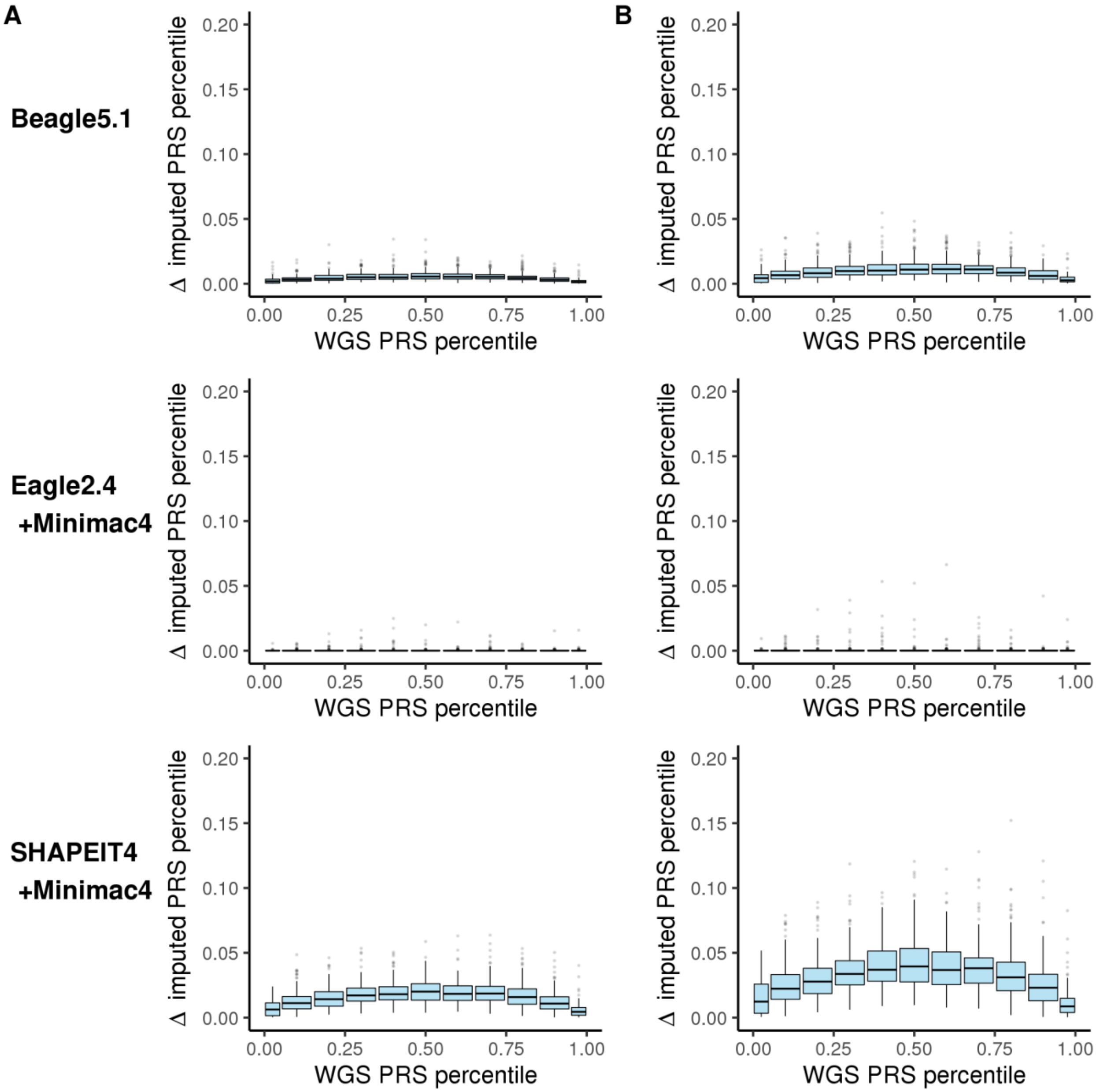

The degree of variability in PRS percentile as a function of the expected WGS-based PRS tier across three different imputation processes. **A.** Average absolute deviation per individual relative to their WGS-based gold standard. **B.** Maximum absolute deviation per individual relative to the their WGS-based gold standard. Box plots depict the interquartile range as is standard.

Fig S33. GPS$_{T2D}$ Variability as a Function of PRS Bin.

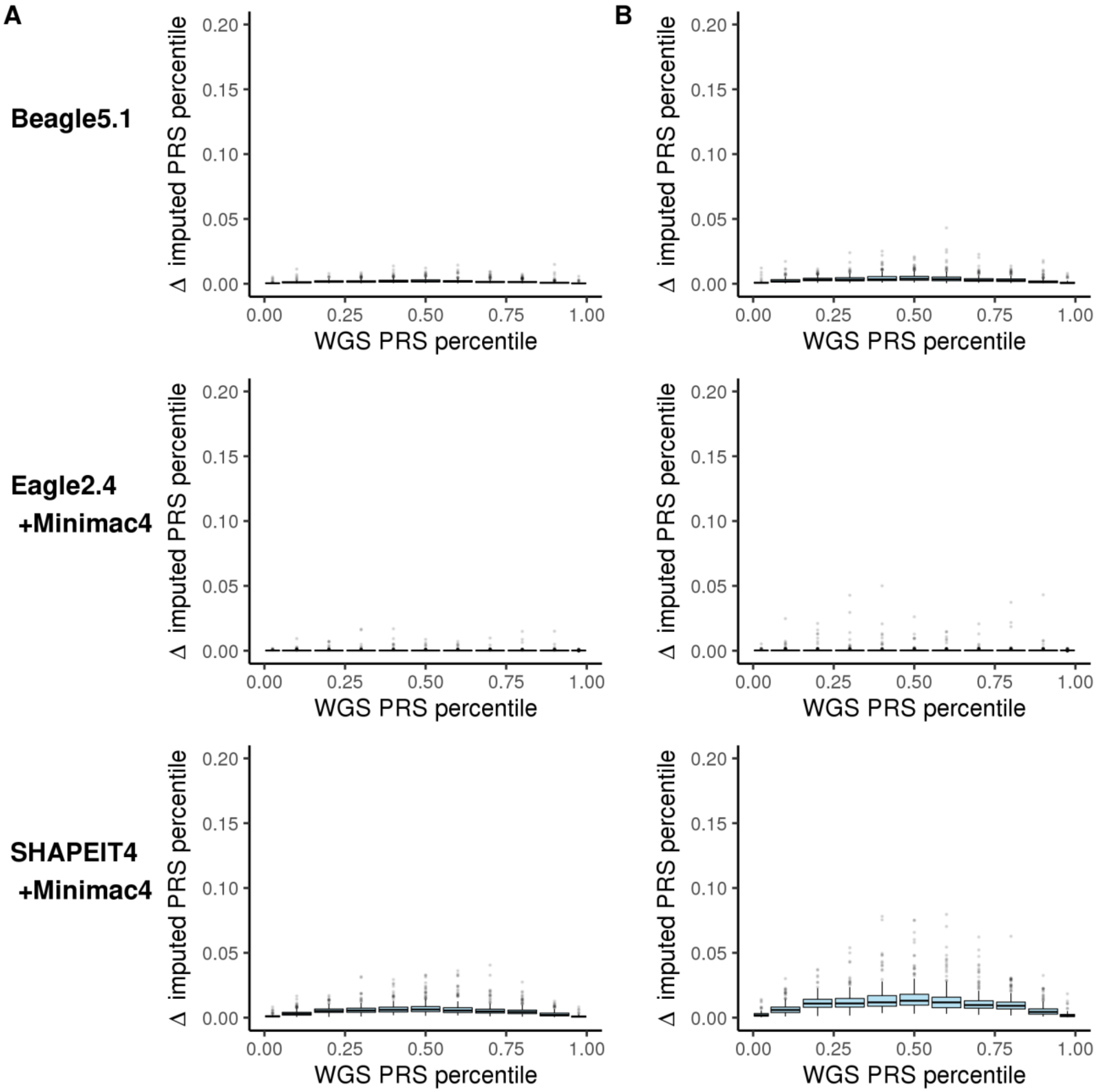

The degree of variability in PRS percentile as a function of the expected WGS-based PRS tier across three different imputation processes. **A.** Average absolute deviation per individual relative to their WGS-based gold standard. **B.** Maximum absolute deviation per individual relative to the their WGS-based gold standard. Box plots depict the interquartile range as is standard.

Fig S34. PRS-GWAS$_{BC}$ (239) Variability as a Function of PRS Bin.

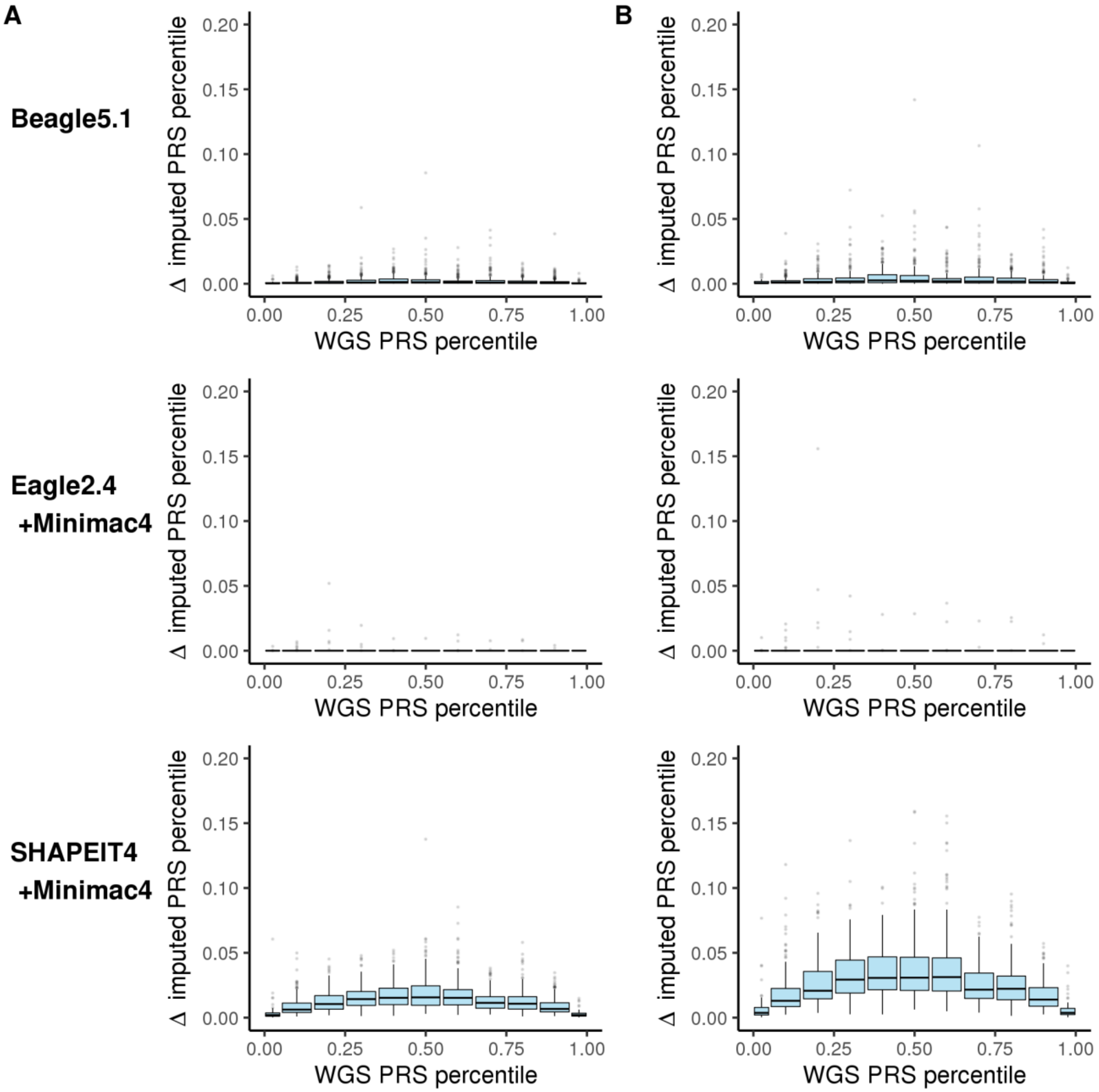

The degree of variability in PRS percentile as a function of the expected WGS-based PRS tier across three different imputation processes. **A.** Average absolute deviation per individual relative to their WGS-based gold standard. **B.** Maximum absolute deviation per individual relative to the their WGS-based gold standard. Box plots depict the interquartile range as is standard.

Fig S35. PRS-GWAS$_{BC}$ (2935) Variability as a Function of PRS Bin.

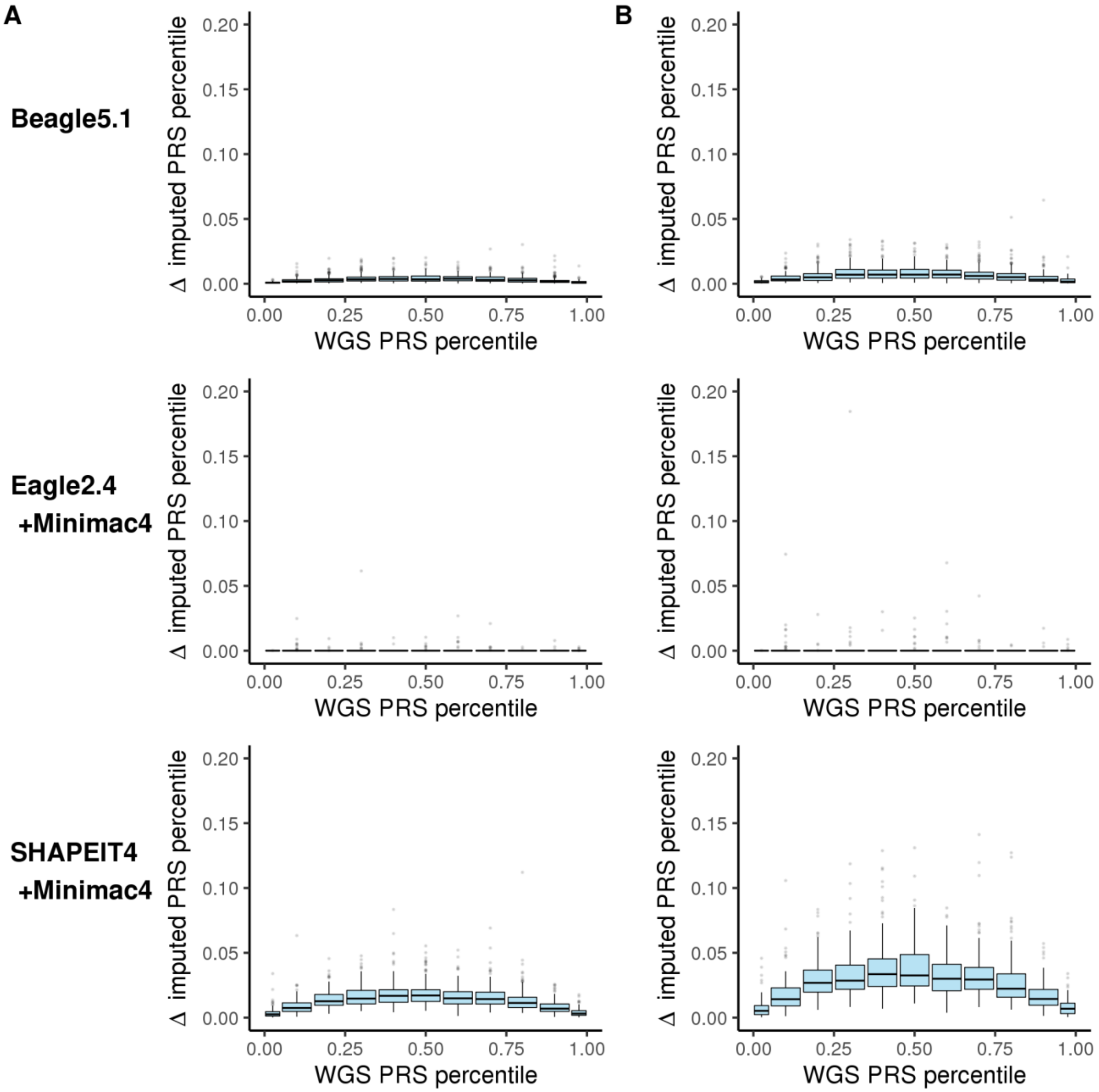

The degree of variability in PRS percentile as a function of the expected WGS-based PRS tier across three different imputation processes. **A.** Average absolute deviation per individual relative to their WGS-based gold standard. **B.** Maximum absolute deviation per individual relative to the their WGS-based gold standard. Box plots depict the interquartile range as is standard.

Fig S36. GPS_{BC} Variability as a Function of PRS Bin.

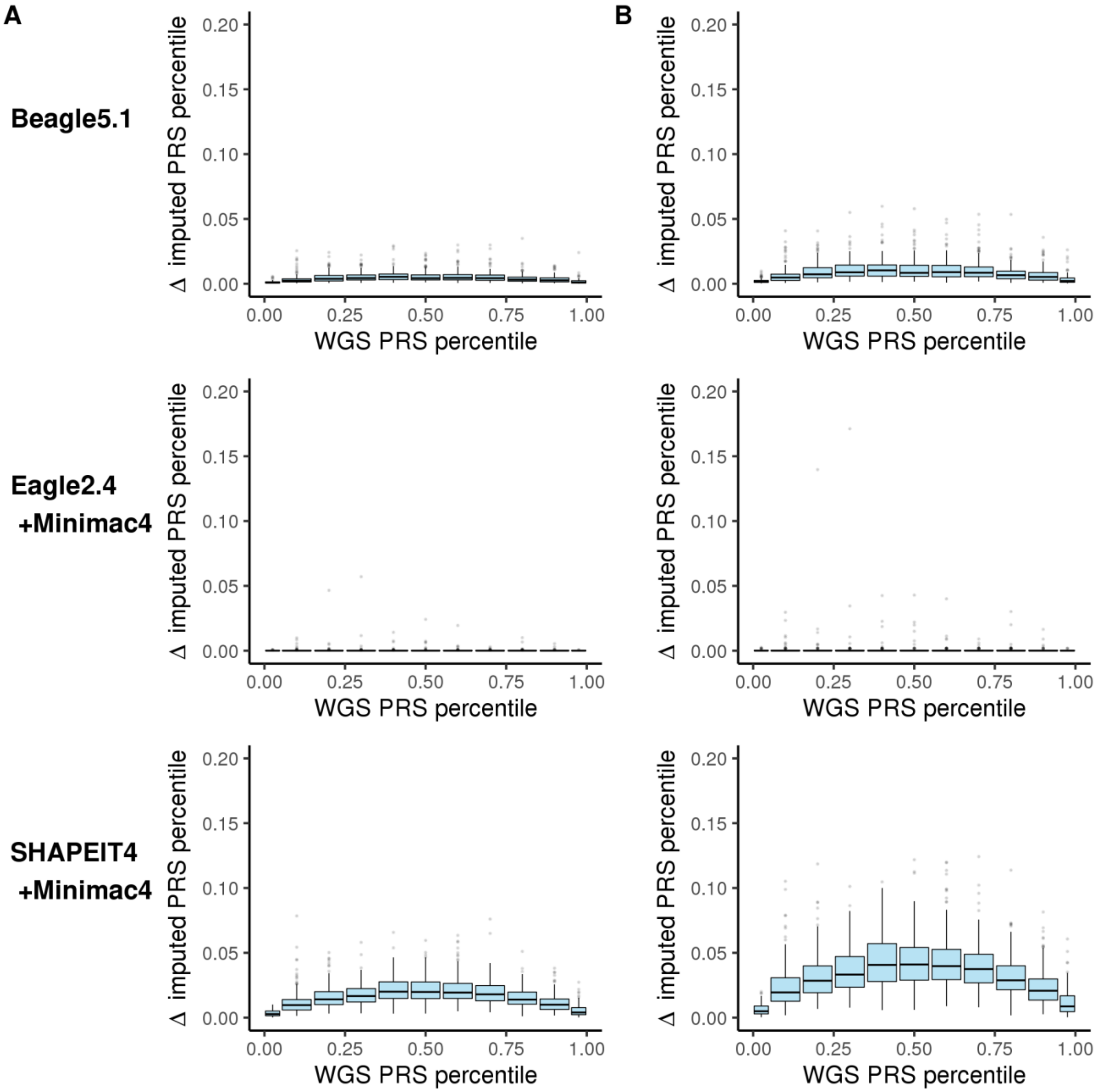

The degree of variability in PRS percentile as a function of the expected WGS-based PRS tier across three different imputation processes. **A.** Average absolute deviation per individual relative to their WGS-based gold standard. **B.** Maximum absolute deviation per individual relative to the their WGS-based gold standard. Box plots depict the interquartile range as is standard.

Fig S37. PRS-GWAS$_{Afib}$ Variability as a Function of PRS Bin.

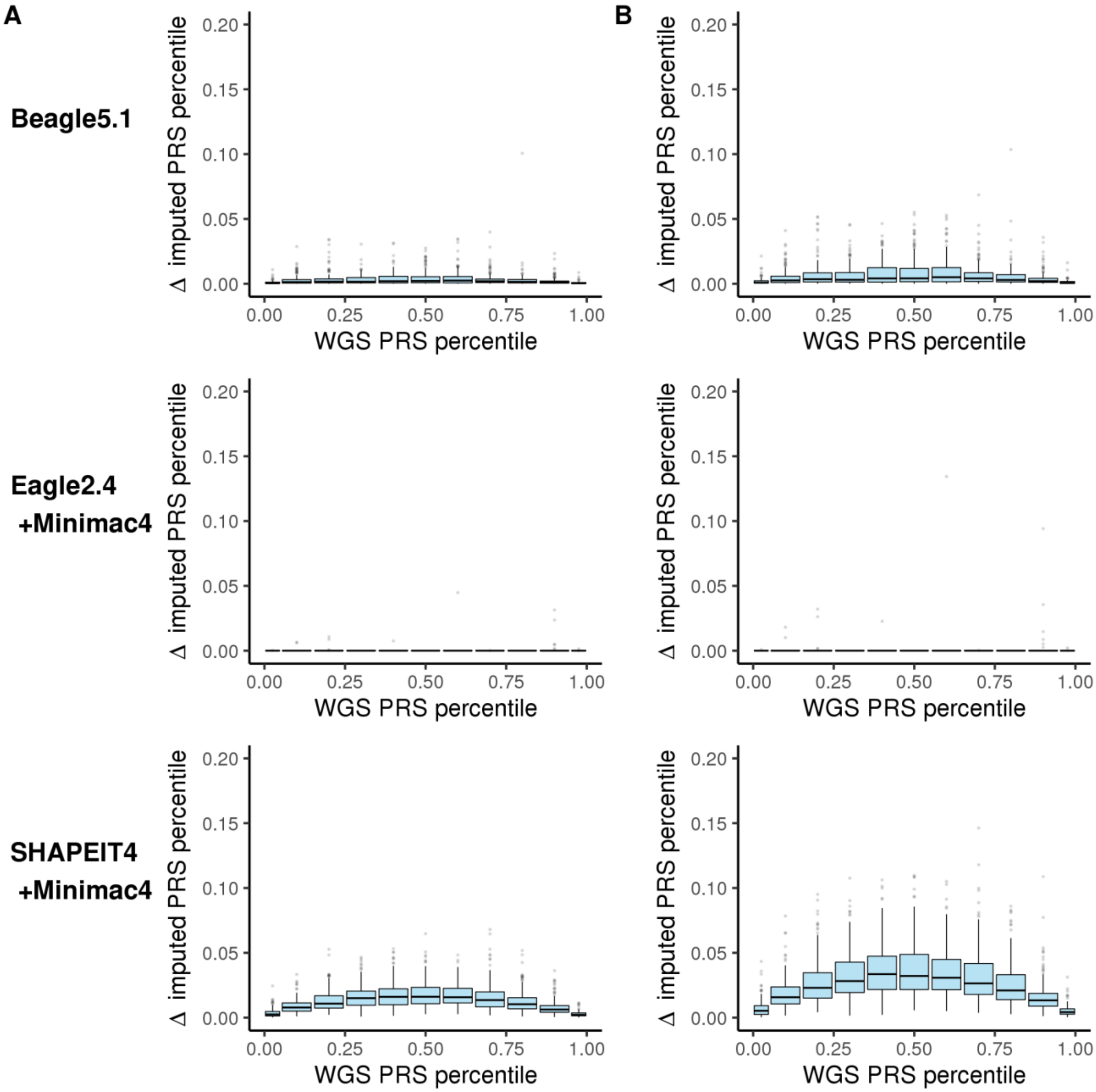

The degree of variability in PRS percentile as a function of the expected WGS-based PRS tier across three different imputation processes. **A.** Average absolute deviation per individual relative to their WGS-based gold standard. **B.** Maximum absolute deviation per individual relative to the their WGS-based gold standard. Box plots depict the interquartile range as is standard.

Fig S38. GPS$_{Afib}$ Variability as a Function of PRS Bin.

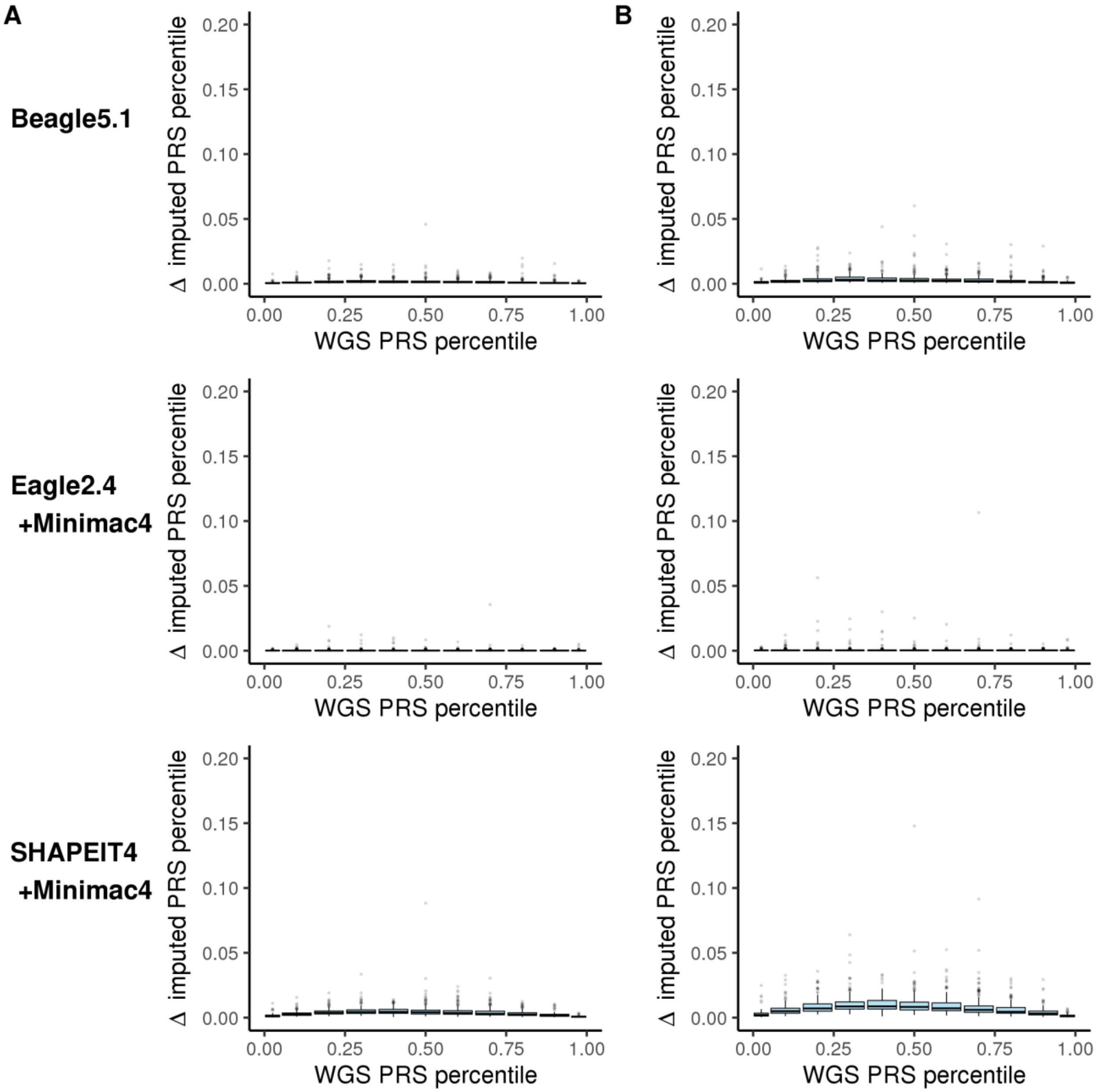

The degree of variability in PRS percentile as a function of the expected WGS-based PRS tier across three different imputation processes. **A.** Average absolute deviation per individual relative to their WGS-based gold standard. **B.** Maximum absolute deviation per individual relative to the their WGS-based gold standard. Box plots depict the interquartile range as is standard.

Fig S39. PRS-GWAS$_{AD}$ Variability as a Function of PRS Bin.

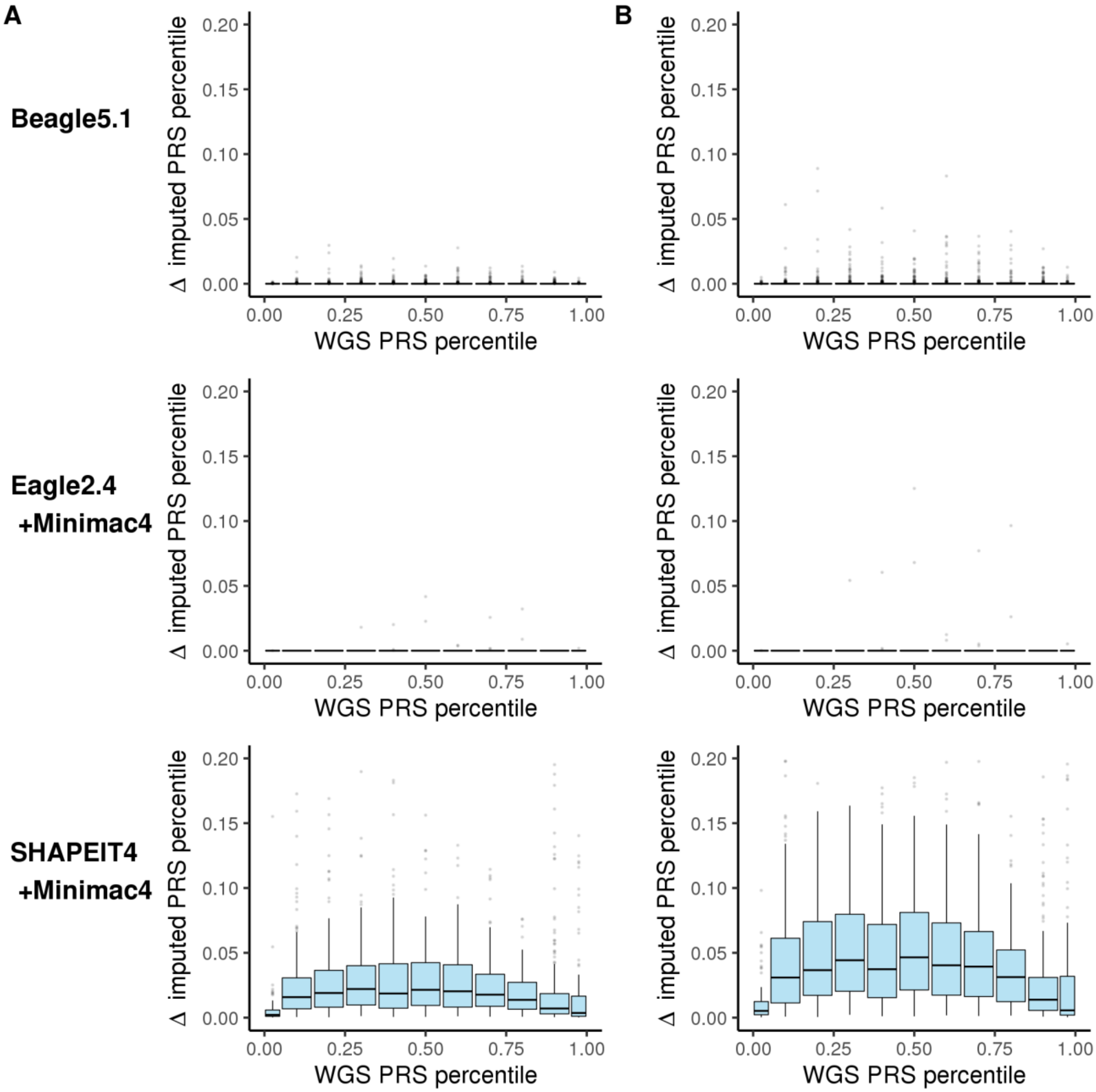

The degree of variability in PRS percentile as a function of the expected WGS-based PRS tier across three different imputation processes. **A.** Average absolute deviation per individual relative to their WGS-based gold standard. **B.** Maximum absolute deviation per individual relative to the their WGS-based gold standard. Box plots depict the interquartile range as is standard.

Fig S40. PRS-GWAS$_{Glaucoma}$ Variability as a Function of PRS Bin.

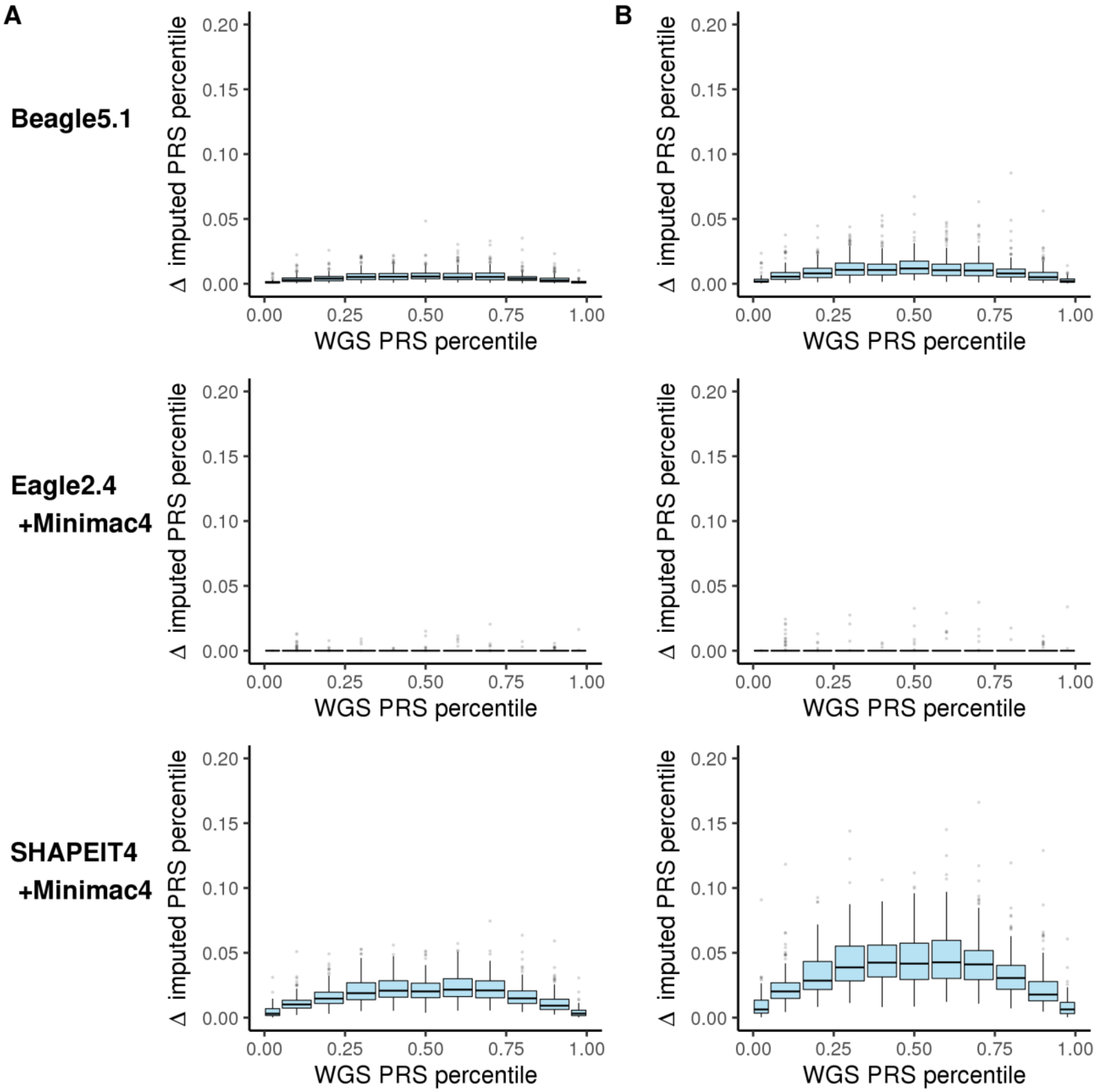

The degree of variability in PRS percentile as a function of the expected WGS-based PRS tier across three different imputation processes. **A.** Average absolute deviation per individual relative to their WGS-based gold standard. **B.** Maximum absolute deviation per individual relative to the their WGS-based gold standard. Box plots depict the interquartile range as is standard.

Fig S41. PRS$_{CAD}$ Variability as a Function of PRS Bin by Ancestry.

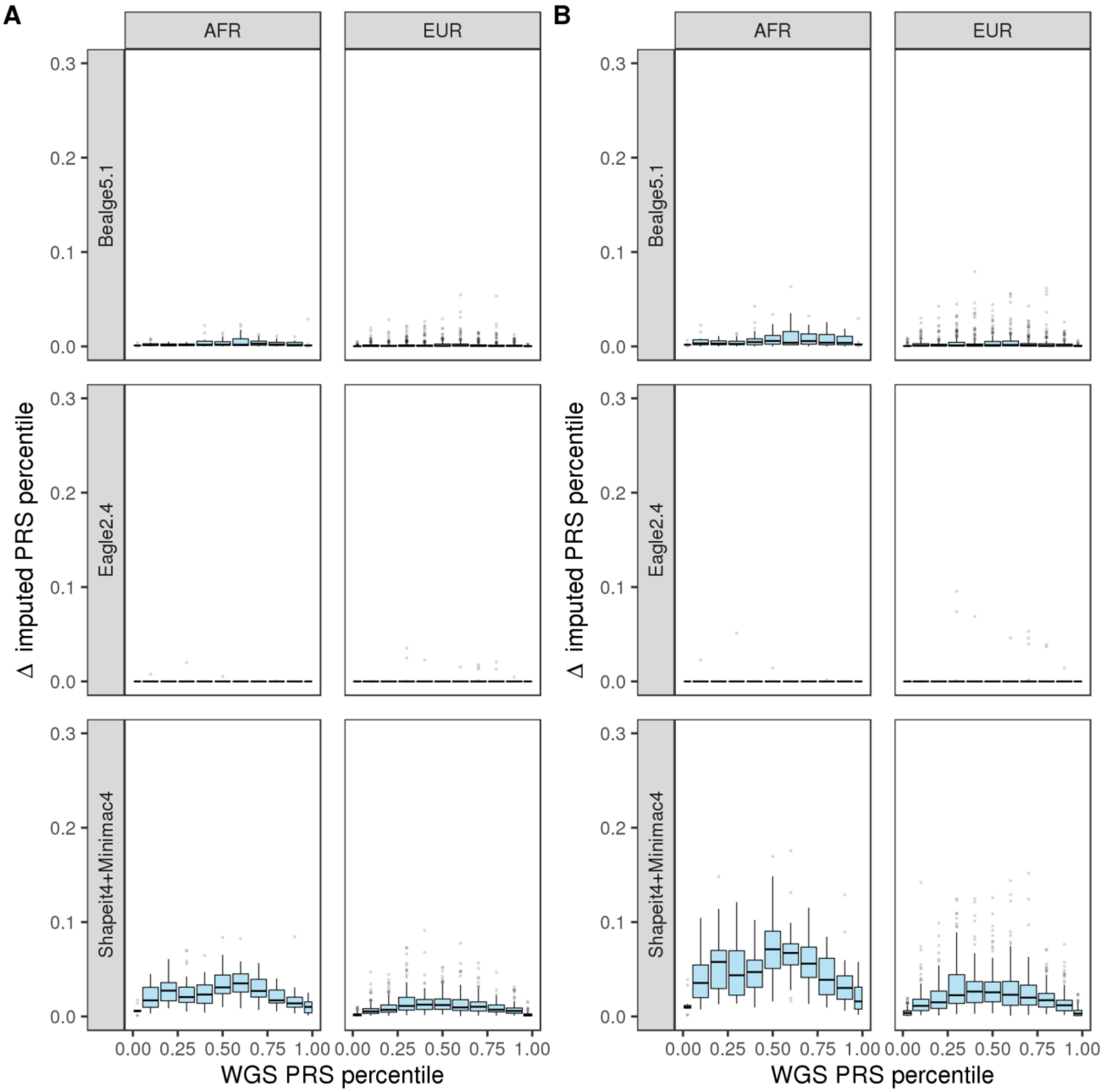

The degree of variability in PRS percentile as a function of the expected WGS-based PRS tier across three different imputation processes by ancestry. **A.** Average absolute deviation per individual relative to their WGS-based gold standard. **B.** Maximum absolute deviation per individual relative to the their WGS-based gold standard. Box plots depict the interquartile range as is standard. AFR: African, EUR: European.

Fig S42. metaGRS$_{CAD}$ Variability as a Function of PRS Bin by Ancestry.

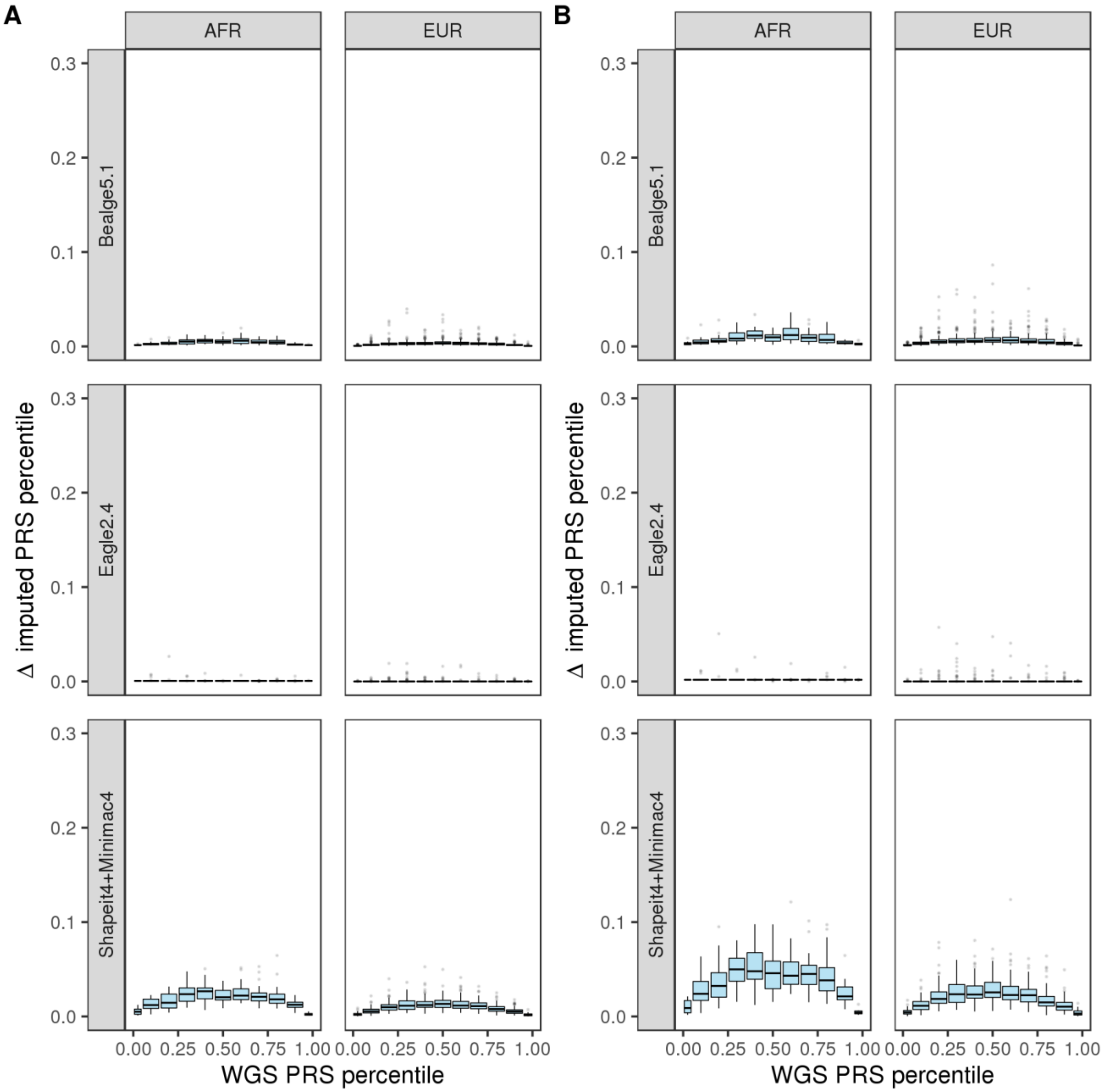

The degree of variability in PRS percentile as a function of the expected WGS-based PRS tier across three different imputation processes by ancestry. **A.** Average absolute deviation per individual relative to their WGS-based gold standard. **B.** Maximum absolute deviation per individual relative to the their WGS-based gold standard. Box plots depict the interquartile range as is standard. AFR: African, EUR: European.

Fig S43. GPS_{CAD} Variability as a Function of PRS Bin by Ancestry.

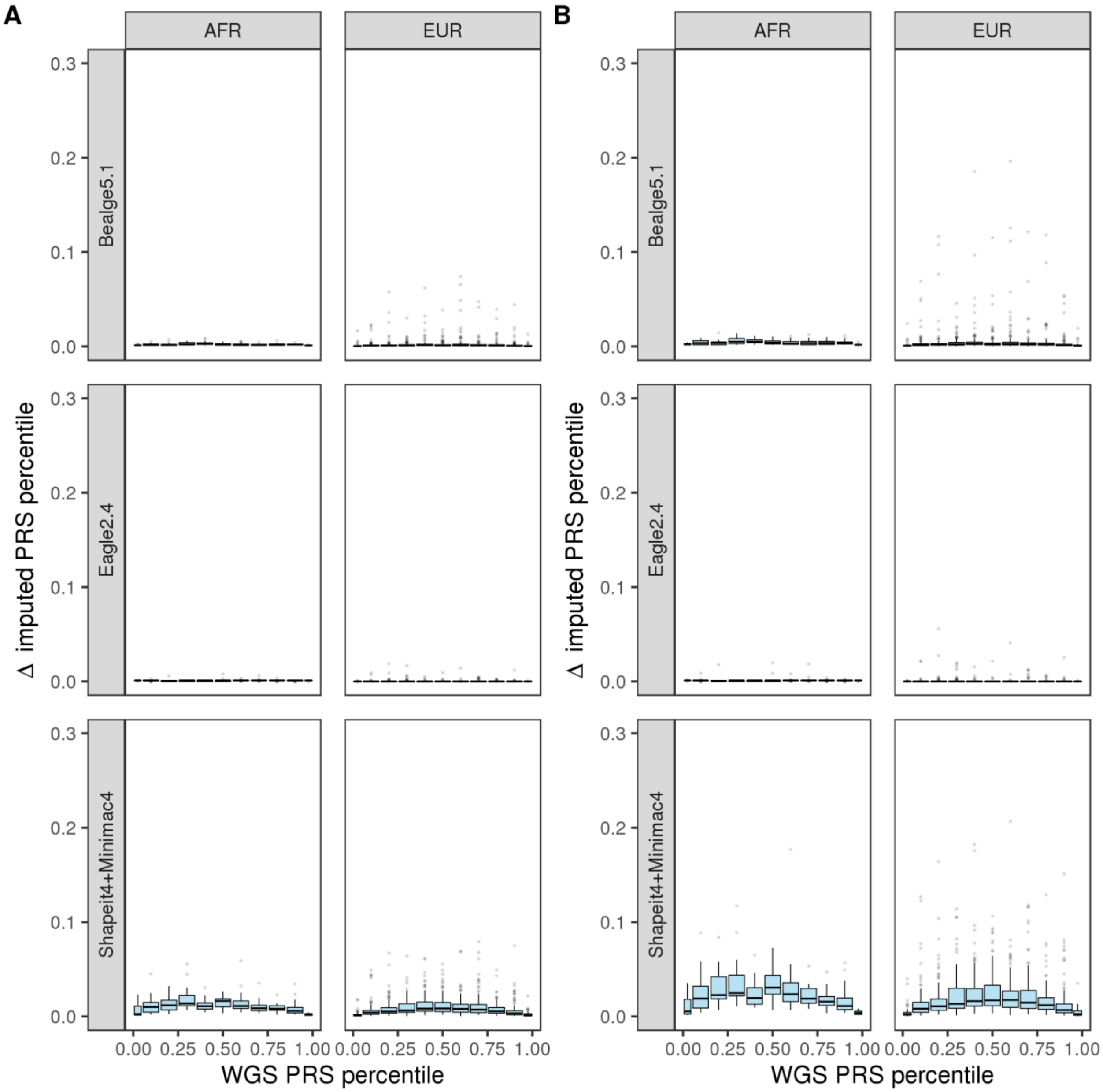

The degree of variability in PRS percentile as a function of the expected WGS-based PRS tier across three different imputation processes by ancestry. **A.** Average absolute deviation per individual relative to their WGS-based gold standard. **B.** Maximum absolute deviation per individual relative to the their WGS-based gold standard. Box plots depict the interquartile range as is standard. AFR: African, EUR: European.

Fig S44. PRS-GWAS$_{T2D}$ (547) Variability as a Function of PRS Bin by Ancestry.

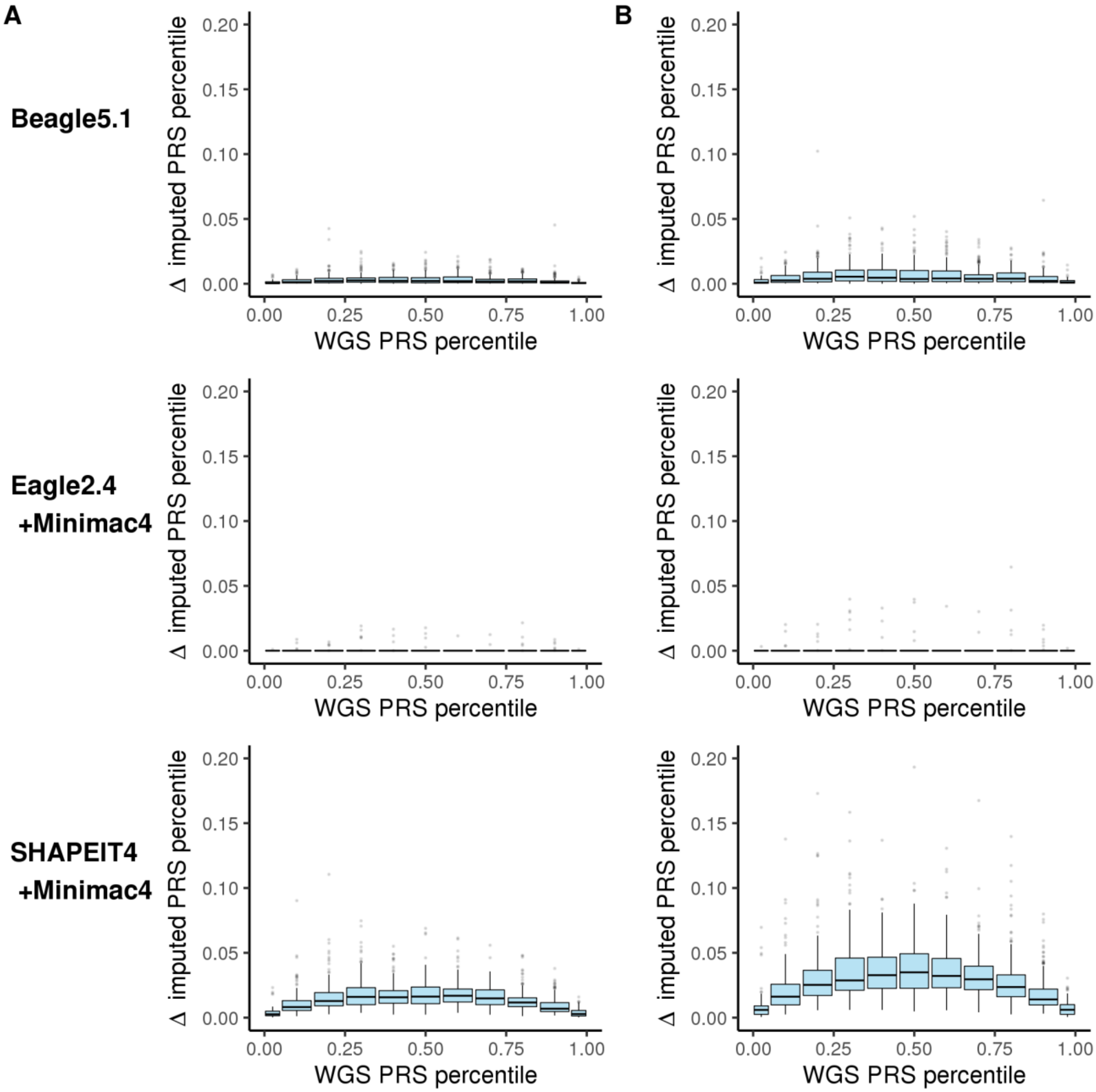

The degree of variability in PRS percentile as a function of the expected WGS-based PRS tier across three different imputation processes by ancestry. **A.** Average absolute deviation per individual relative to their WGS-based gold standard. **B.** Maximum absolute deviation per individual relative to the their WGS-based gold standard. Box plots depict the interquartile range as is standard. AFR: African, EUR: European.

Fig S45. PRS-$GWAS_{T2D}$ (397) Variability as a Function of PRS Bin by Ancestry.

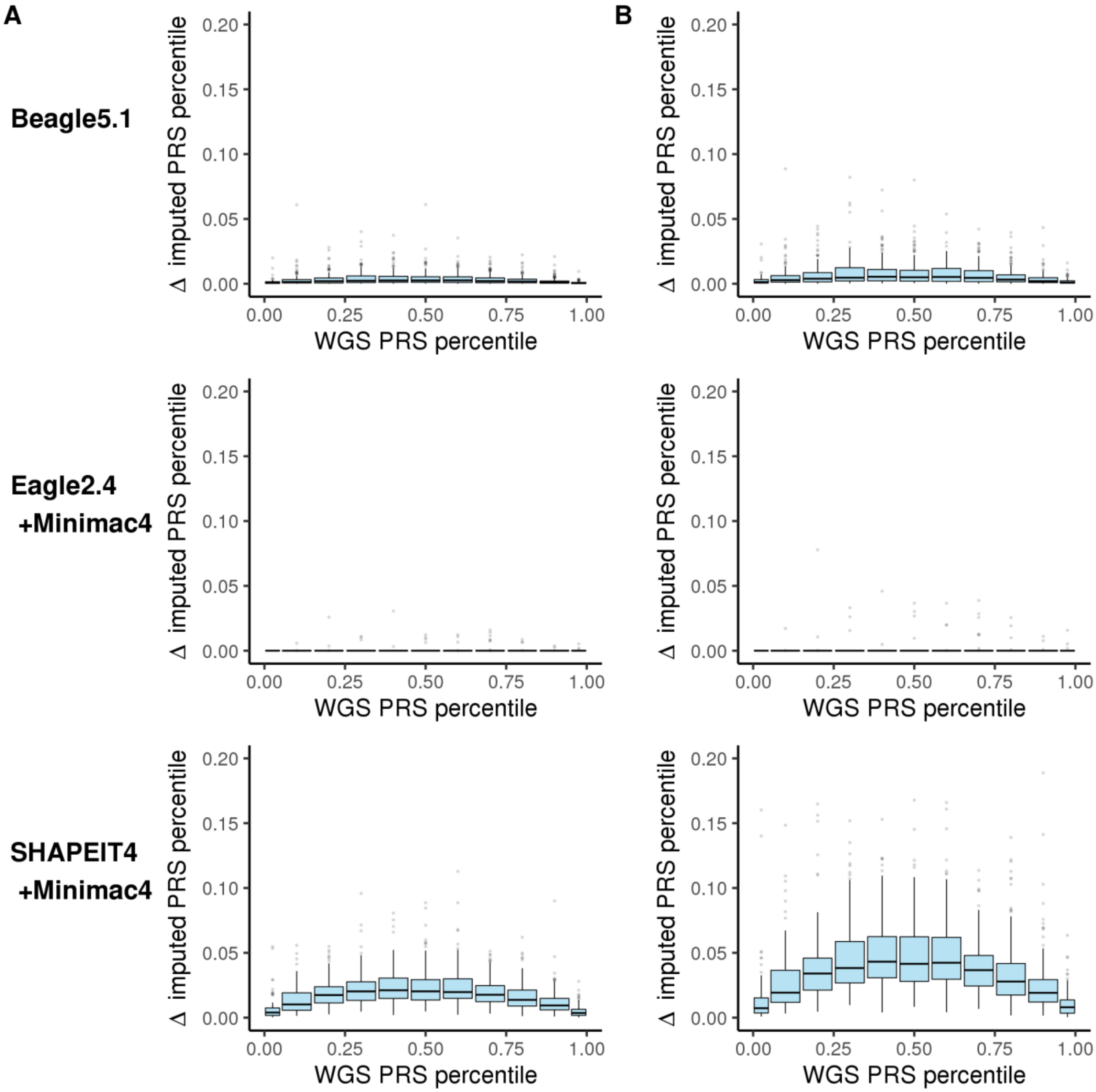

The degree of variability in PRS percentile as a function of the expected WGS-based PRS tier across three different imputation processes by ancestry. **A.** Average absolute deviation per individual relative to their WGS-based gold standard. **B.** Maximum absolute deviation per individual relative to the their WGS-based gold standard. Box plots depict the interquartile range as is standard. AFR: African, EUR: European.

Fig S46. PRS-GWAS$_{T2D}$ (170487) Variability as a Function of PRS Bin by Ancestry.

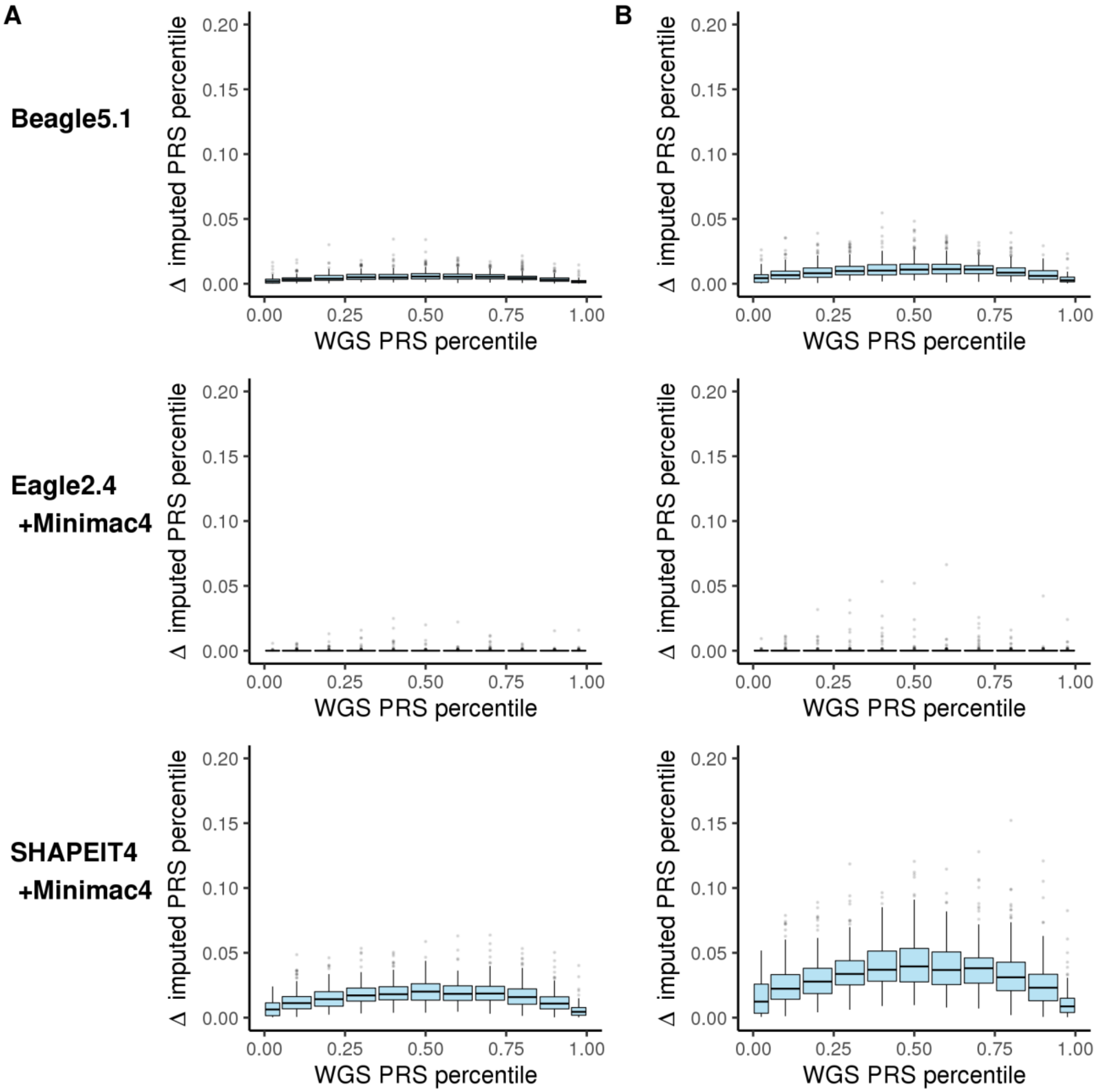

The degree of variability in PRS percentile as a function of the expected WGS-based PRS tier across three different imputation processes by ancestry. **A.** Average absolute deviation per individual relative to their WGS-based gold standard. **B.** Maximum absolute deviation per individual relative to the their WGS-based gold standard. Box plots depict the interquartile range as is standard. AFR: African, EUR: European.

Fig S47. GPS_{T2D} Variability as a Function of PRS Bin by Ancestry.

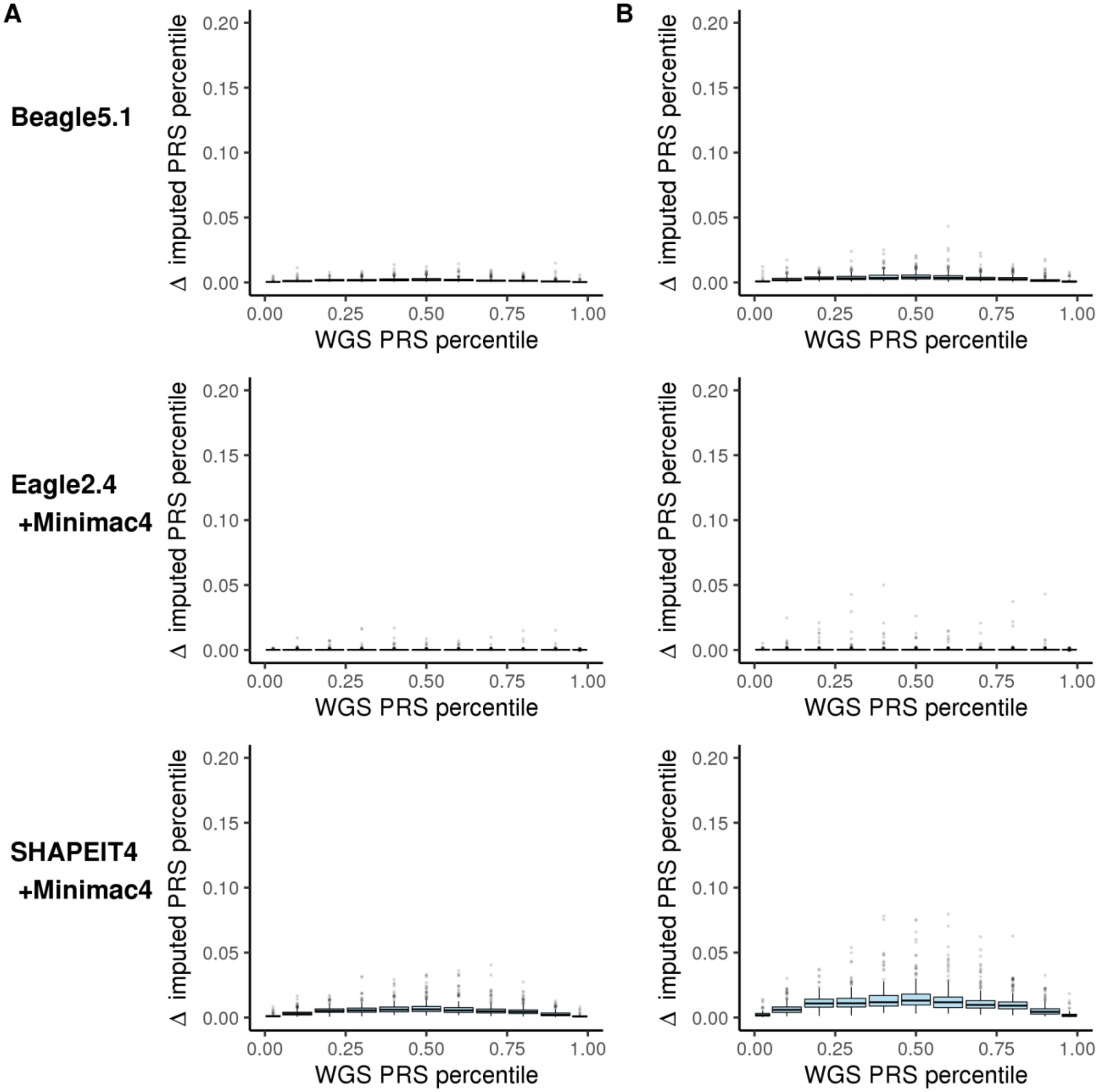

The degree of variability in PRS percentile as a function of the expected WGS-based PRS tier across three different imputation processes by ancestry. **A.** Average absolute deviation per individual relative to their WGS-based gold standard. **B.** Maximum absolute deviation per individual relative to the their WGS-based gold standard. Box plots depict the interquartile range as is standard. AFR: African, EUR: European.

Fig S48. PRS-GWAS$_{BC}$ (239) Variability as a Function of PRS Bin by Ancestry.

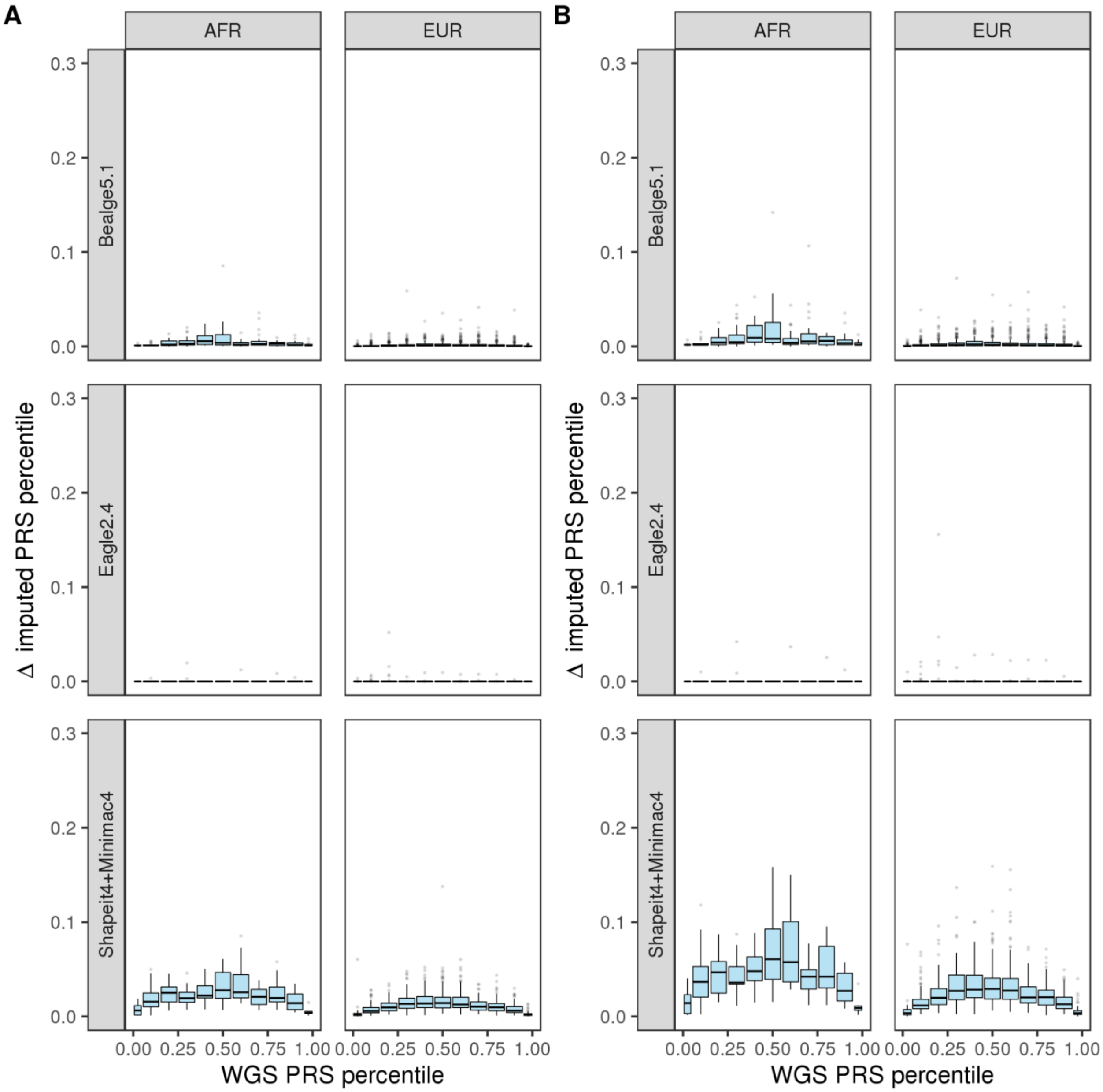

The degree of variability in PRS percentile as a function of the expected WGS-based PRS tier across three different imputation processes by ancestry. **A.** Average absolute deviation per individual relative to their WGS-based gold standard. **B.** Maximum absolute deviation per individual relative to the their WGS-based gold standard. Box plots depict the interquartile range as is standard. AFR: African, EUR: European.

Fig S49. PRS-GWAS$_{BC}$ (2935) Variability as a Function of PRS Bin by Ancestry.

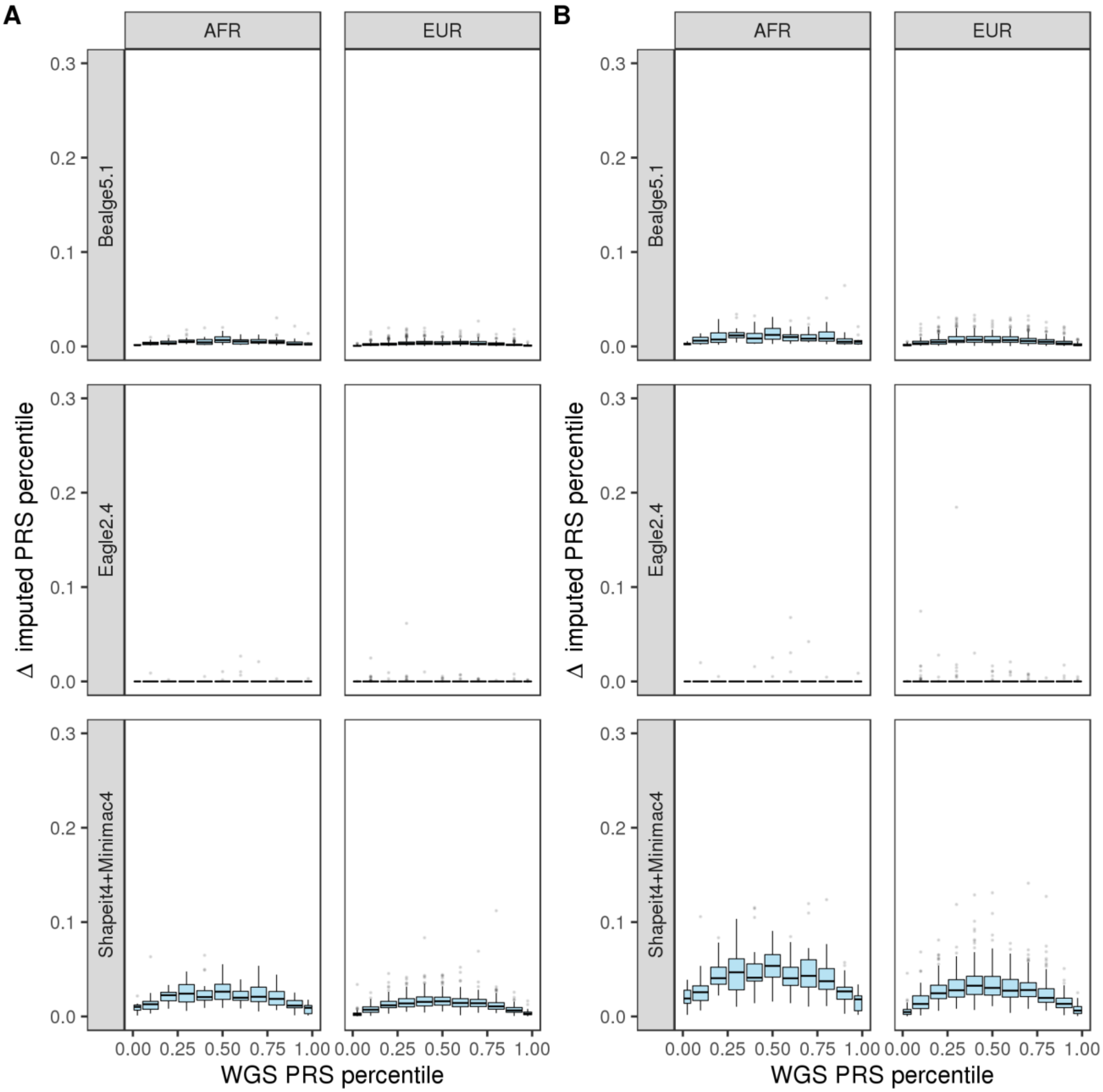

The degree of variability in PRS percentile as a function of the expected WGS-based PRS tier across three different imputation processes by ancestry. **A.** Average absolute deviation per individual relative to their WGS-based gold standard. **B.** Maximum absolute deviation per individual relative to the their WGS-based gold standard. Box plots depict the interquartile range as is standard. AFR: African, EUR: European.

Fig S50. GPS$_{BC}$ Variability as a Function of PRS Bin by Ancestry.

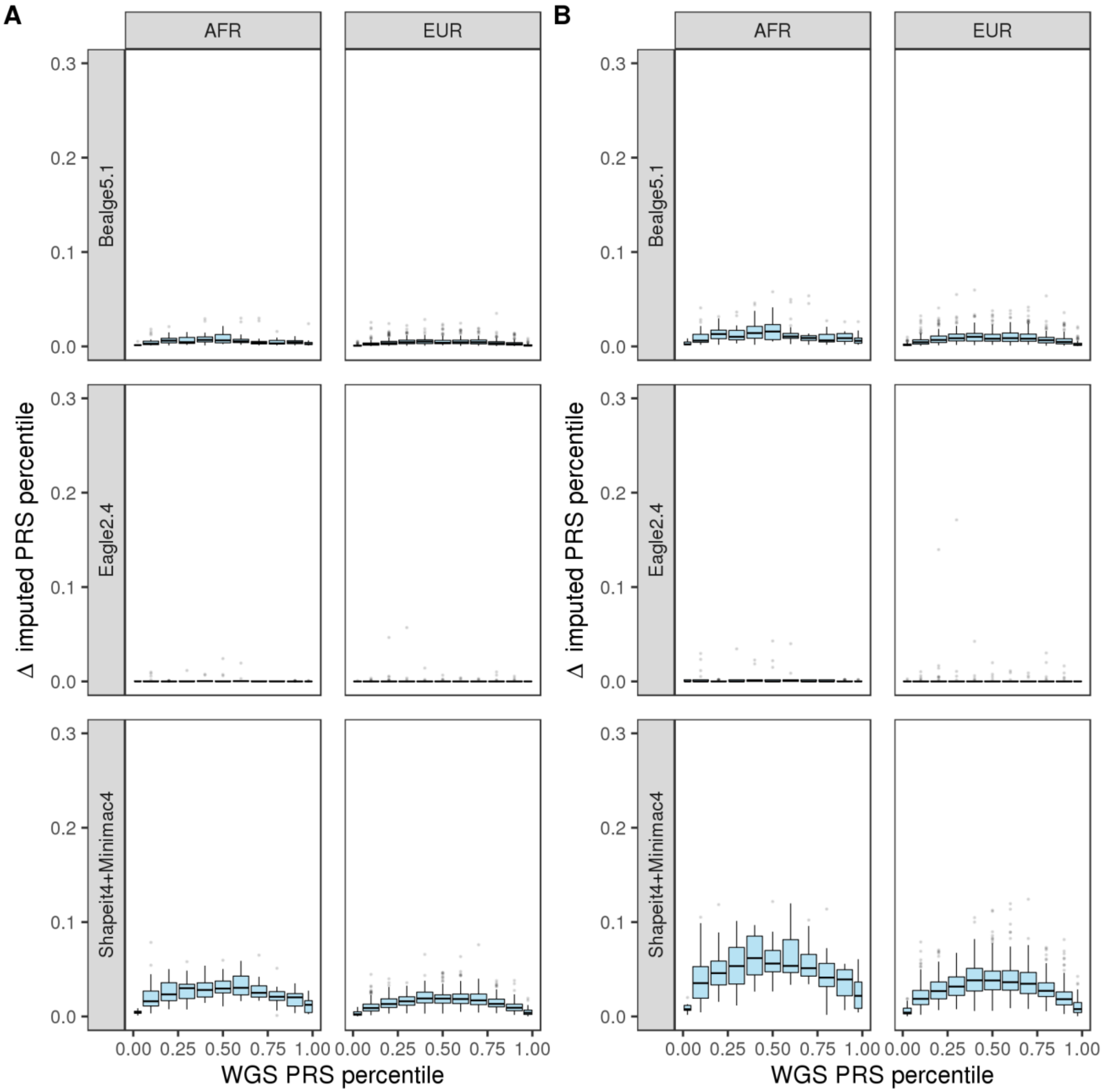

The degree of variability in PRS percentile as a function of the expected WGS-based PRS tier across three different imputation processes by ancestry. **A.** Average absolute deviation per individual relative to their WGS-based gold standard. **B.** Maximum absolute deviation per individual relative to the their WGS-based gold standard. Box plots depict the interquartile range as is standard. AFR: African, EUR: European.

Fig S51. PRS-GWAS$_{Afib}$ Variability as a Function of PRS Bin by Ancestry.

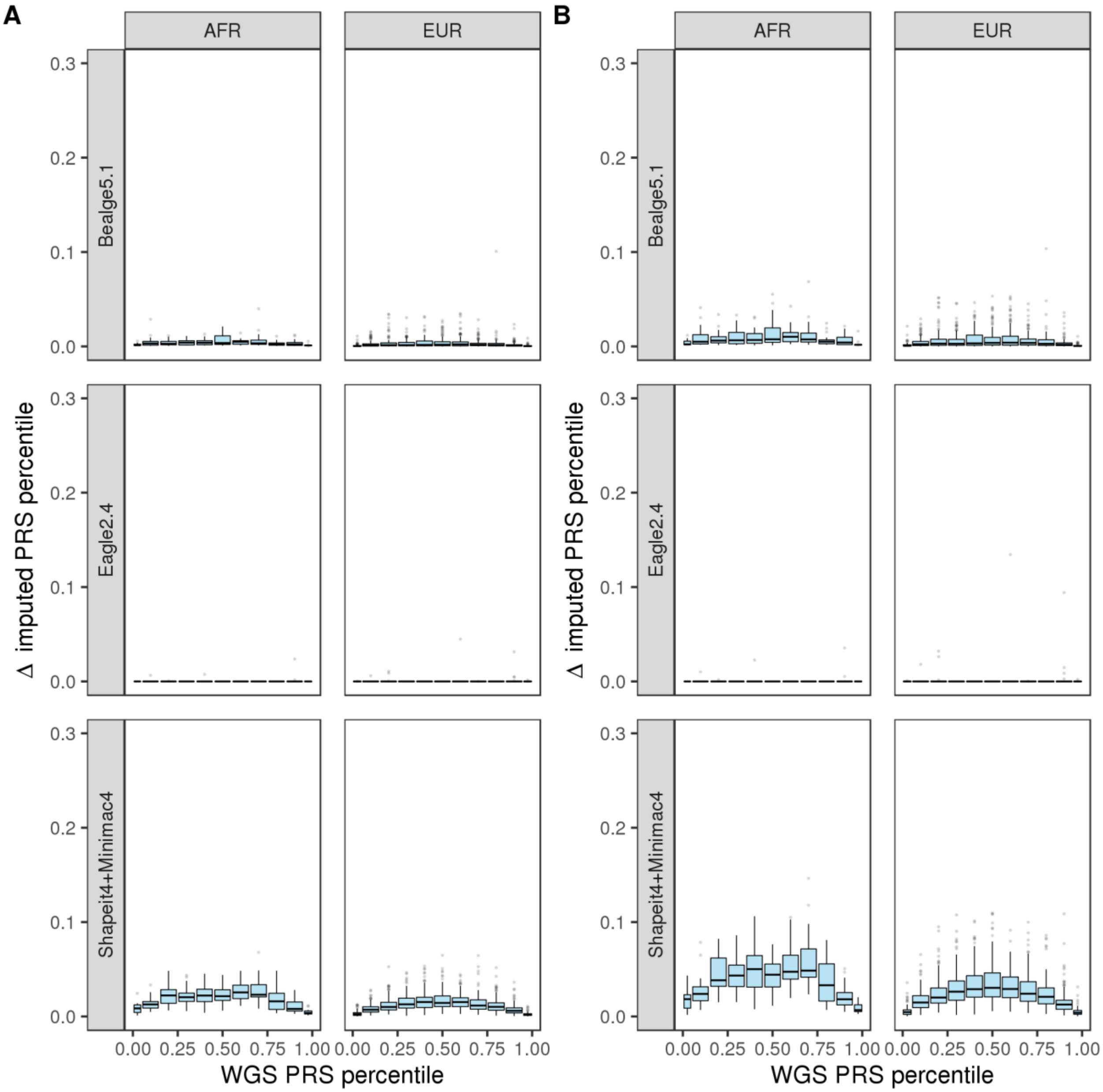

The degree of variability in PRS percentile as a function of the expected WGS-based PRS tier across three different imputation processes by ancestry. **A.** Average absolute deviation per individual relative to their WGS-based gold standard. **B.** Maximum absolute deviation per individual relative to the their WGS-based gold standard. Box plots depict the interquartile range as is standard. AFR: African, EUR: European.

Fig S52. GPS_{Afib} Variability as a Function of PRS Bin by Ancestry.

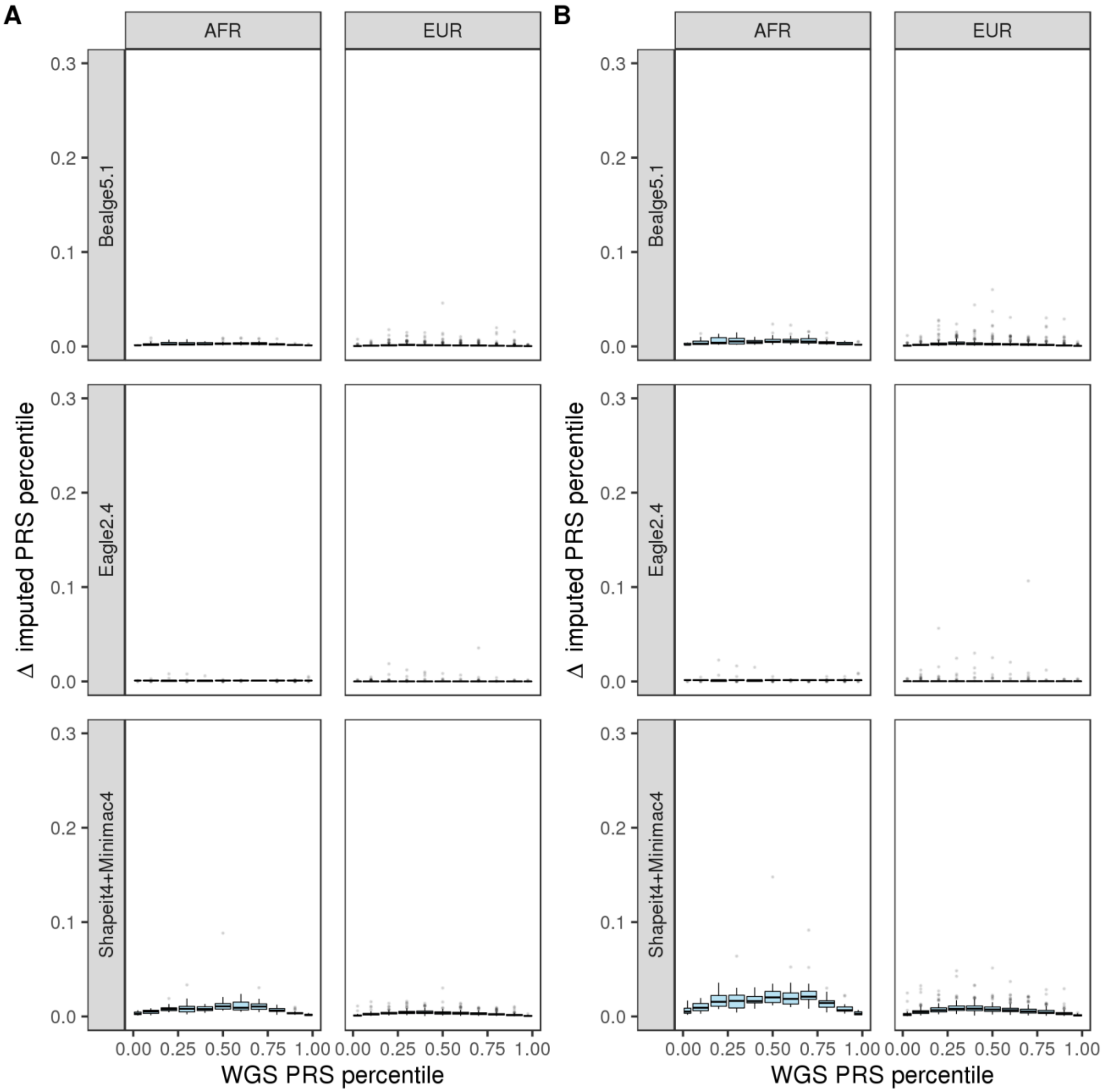

The degree of variability in PRS percentile as a function of the expected WGS-based PRS tier across three different imputation processes by ancestry. **A.** Average absolute deviation per individual relative to their WGS-based gold standard. **B.** Maximum absolute deviation per individual relative to the their WGS-based gold standard. Box plots depict the interquartile range as is standard. AFR: African, EUR: European.

Fig S53. PRS-GWAS$_{AD}$ Variability as a Function of PRS Bin by Ancestry.

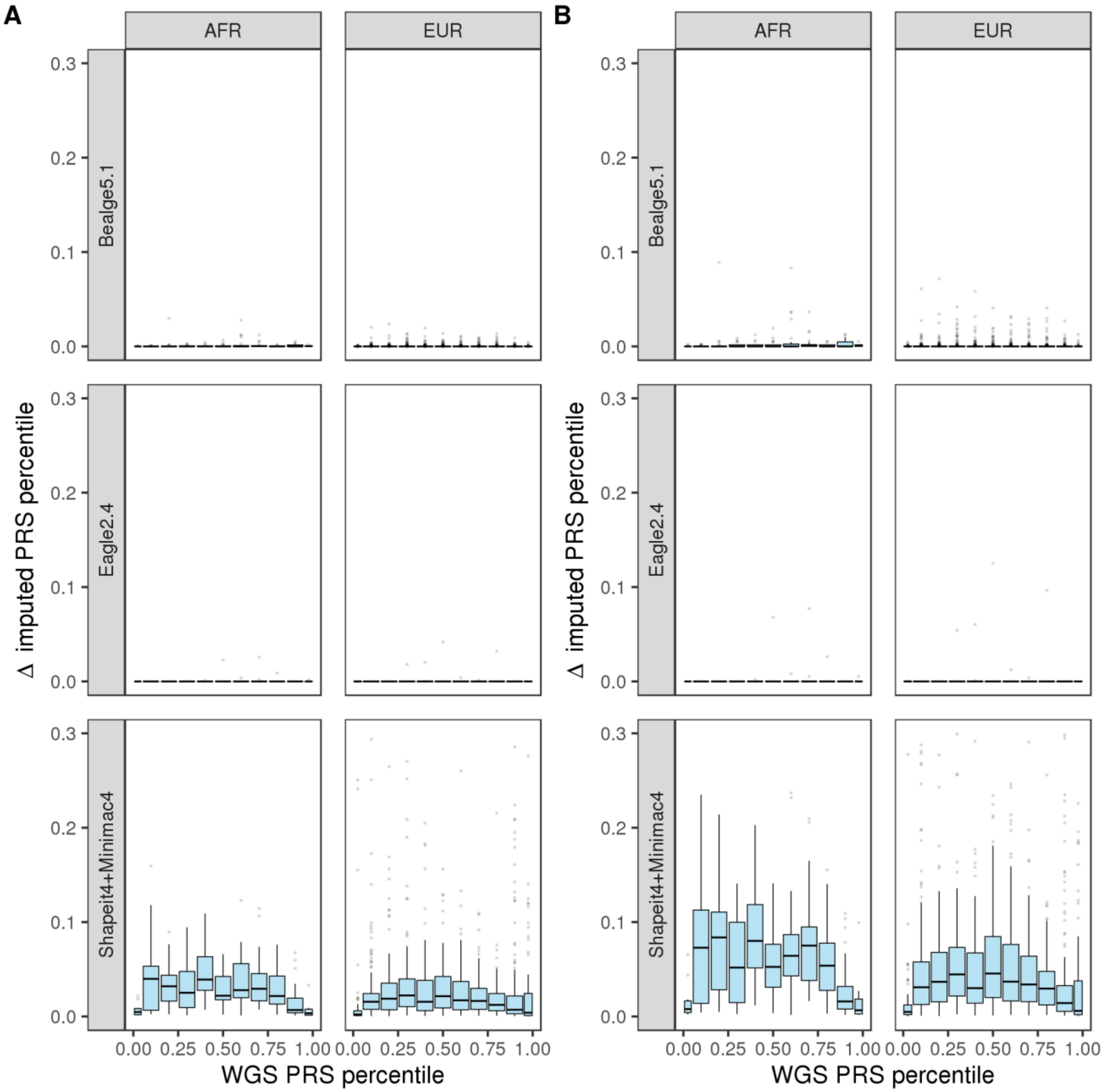

The degree of variability in PRS percentile as a function of the expected WGS-based PRS tier across three different imputation processes by ancestry. **A.** Average absolute deviation per individual relative to their WGS-based gold standard. **B.** Maximum absolute deviation per individual relative to the their WGS-based gold standard. Box plots depict the interquartile range as is standard. AFR: African, EUR: European.

Fig S54. PRS-GWAS$_{Glaucoma}$ Variability as a Function of PRS Bin by Ancestry.

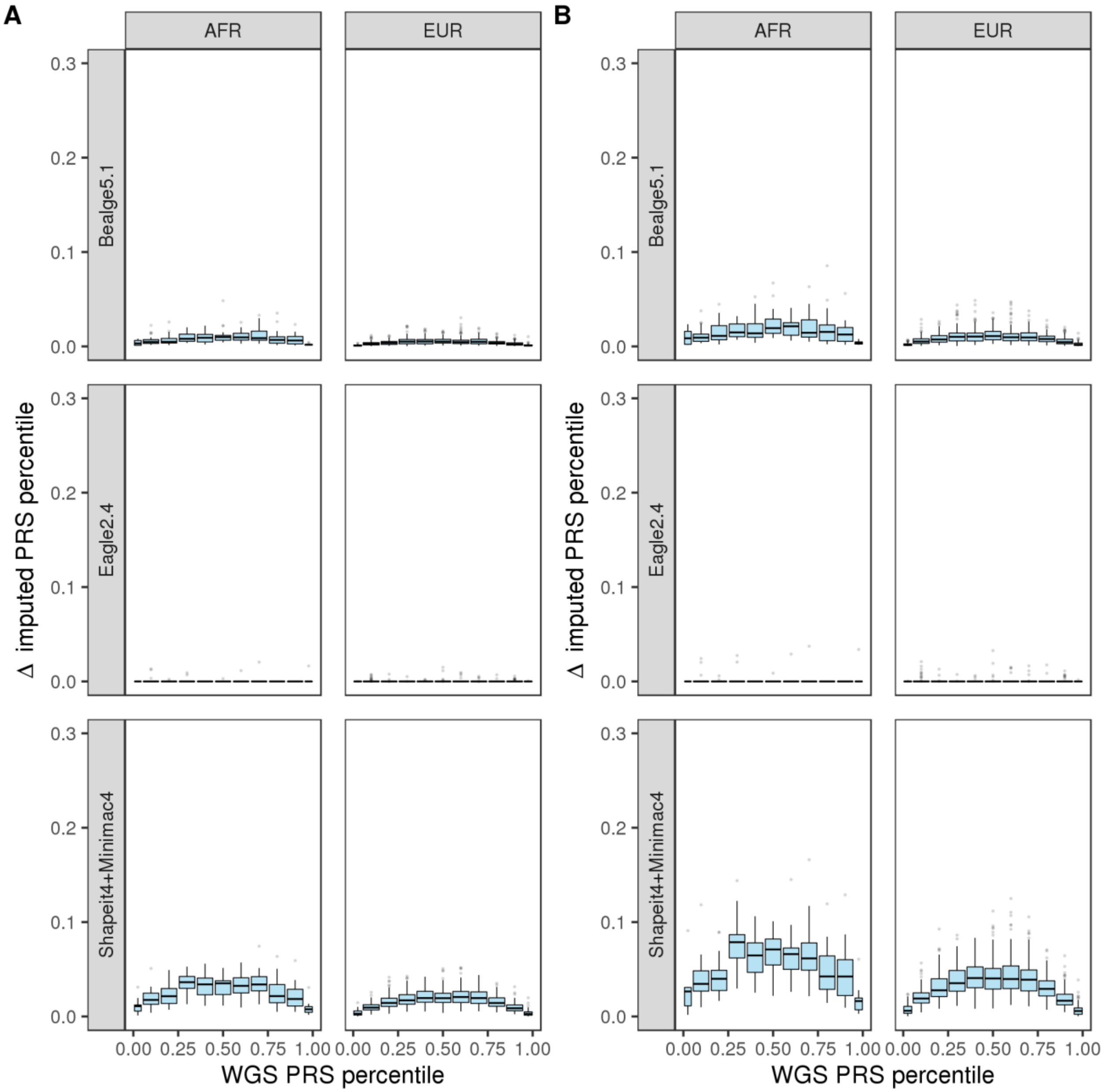

The degree of variability in PRS percentile as a function of the expected WGS-based PRS tier across three different imputation processes by ancestry. **A.** Average absolute deviation per individual relative to their WGS-based gold standard. **B.** Maximum absolute deviation per individual relative to the their WGS-based gold standard. Box plots depict the interquartile range as is standard. AFR: African, EUR: European.

Fig S55. SNP level variability by Score Impact Across Diseases and Score Derivation Methods.

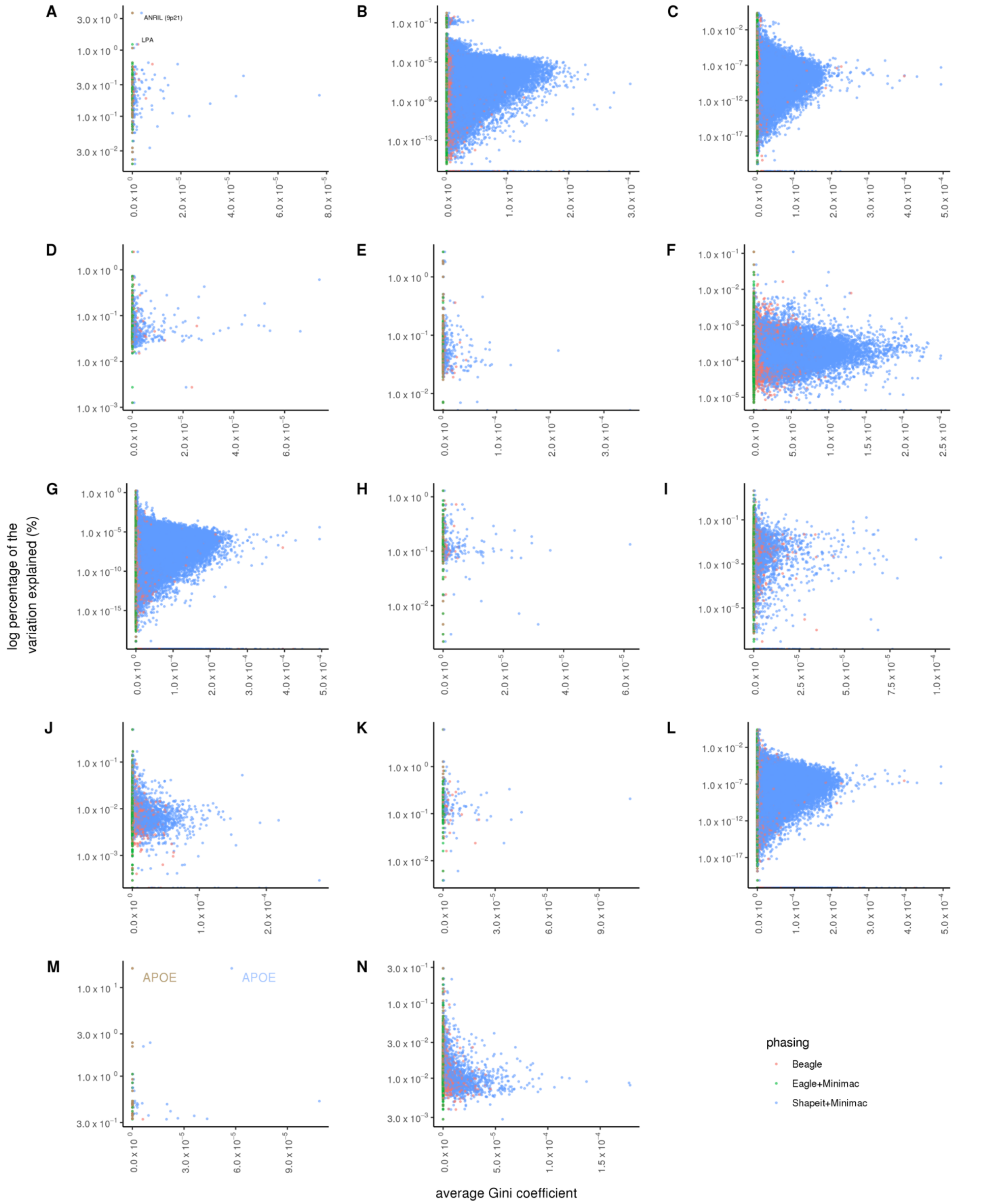

Scatterplot of variation explained per SNP for **A.** PRS$_{CAD}$ (161) **B.** metaGRS$_{CAD}$ (1736608) **C.** GPS$_{CAD}$ (6238460) **D.** PRS-GWAS$_{T2D}$ (547). **E.** PRS-GWAS$_{T2D}$ (397). **F.** PRS-GWAS$_{T2D}$ (170487). **G.** GPS$_{T2D}$ (6482889). **H.** PRS-GWAS$_{BC}$ (239). **I.** PRS-GWAS$_{BC}$ (2941). **J.** GPS$_{BC}$ (4457) **K.** PRS-GWAS$_{Afib}$ (166). **L.** GPS$_{Afib}$ (6302924). **M.** PRS-GWAS$_{AD}$ (29). **N.** PRS-GWAS$_{Glaucoma}$ (2657).
